# Calcium waste as a catalyst in the transesterification for demanding esters: scalability perspective

**DOI:** 10.3762/bjoc.21.114

**Published:** 2025-07-28

**Authors:** Anton N Potorochenko, Konstantin S Rodygin

**Affiliations:** 1 Institute of Chemistry, Saint Petersburg State University, 7/9 Universitetskaya nab., St. Petersburg, 199034, Russia

**Keywords:** biodiesel, calcium oxide, carbide slag, ester, transesterification

## Abstract

Esters are valuable compounds in fine organic synthesis and industry. The significant growth in the demand for esters requires the development of scalable production methods. Heterogeneous CaO-based catalysts for the production of esters by transesterification are promising catalytic systems for the production of these desired compounds. In this work, the application of calcium carbide slag, a byproduct of acetylene production, was investigated. The catalyst was obtained by calcination of calcium carbide slag at 600 °C (CS_600_) and characterized by XRD and FTIR analysis. The transesterification reactions were carried out with primary alcohols, producing fatty acid alkyl esters in 51–99% yields, depending on the alcohols’ nature and catalyst amount (1–10 wt %). The CS_600_ catalyst demonstrated efficiency in the transesterification of low-molecular-weight esters, medium-chain triglycerides (C_9_–C_12_), and lactones, resulting in the corresponding methyl esters in 66–99% yields in the presence of low catalyst amounts of 1–5 wt %.

## Introduction

The ester moiety in molecules is one of the most important functional groups, which is widespread in nature, products of fine organic synthesis, and large-scale chemical manufacturing [1–7]. Nowadays, esters have found indispensable applications as solvents [8–11], biolubricants [12–15], food additives [16–19], polyester plastics and materials [20–25], in the pharmaceutical industry [26–30], in perfumes and flavoring [31–34], and cosmetic industries [16–17,35–36]. Fatty acid methyl esters (biodiesel) are of particular interest due to their current use as fuel in vehicles and promising applications [37–43].

The esters are demanded in large amounts according to available methods for their manufacturing. The transesterification approach is an efficient way, which requires the use of a catalyst [44–48] and of course, there are many catalysts providing the desired transesterification products. However, the availability of the catalysts is limited, and the scope of industrially compatible catalysts is very narrow. The absence of an available large-tonnage catalyst is the principal limitation to the industrial production of commercially demanded esters.

Calcium carbide slag, a waste product from acetylene production via hydrolysis of calcium carbide [49], was successfully applied as a transesterification catalyst for biodiesel production [50–57]. The amount of carbide slag generated is significant because of the many applications in chemistry [58–61] and industry as a large-tonnage product. Carbide slag was successfully utilized in various applications [62–71]; however, the amount of slag is much higher than its actual consumption.

In this work, the application of a catalyst derived from carbide slag in the transesterification of soybean oil with various alcohols (11 examples) was investigated. The carbide slag was calcined at 600 °C before use and the catalyst CS_600_ was characterized using XRD and FTIR analysis, confirming the presence of CaO as the main phase. The primary alcohols successfully reacted in the transesterification reaction to give the corresponding fatty acid alkyl ester mixtures in yields ranging from 51% to 99%, depending on the alcohol and catalyst loading (1–10 wt %). Alcohols with additional functional groups were converted to the respective esters suitable for further modifications. The CS_600_ catalyst proved promising for the transesterification of low-molecular-weight esters, medium-chain triglycerides (C_9_–C_12_), and lactones with yields ranging from 66% to 99% at catalyst loadings of 1–5 wt % (9 examples). For the first time, transesterification of various esters was carried out using a catalyst from carbide slag waste. The use of the catalyst demonstrated its compatibility with fine organic synthesis.

## Results and Discussion

**Figure 1:**
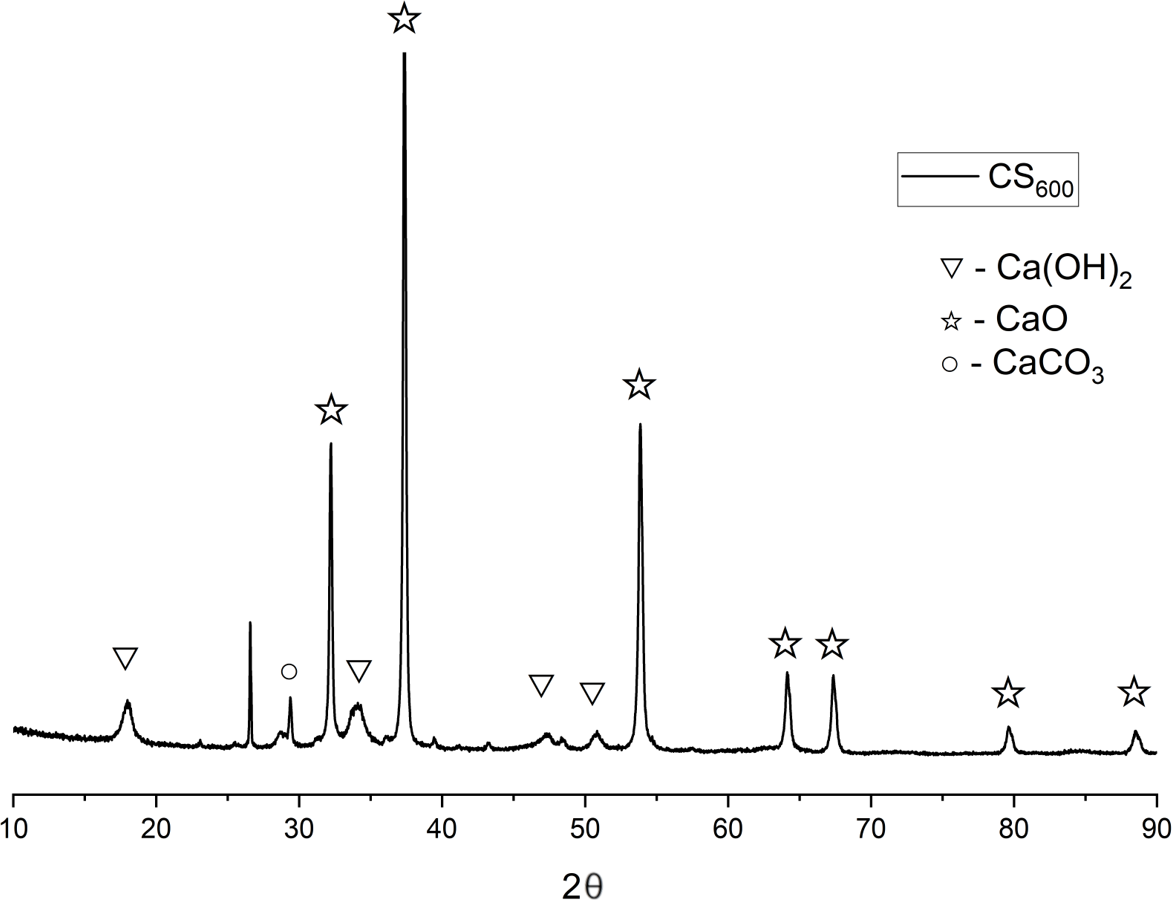
XRD pattern of CS_600_.

The carbide slag was prepared by hydrolysis of commercially available calcium carbide (Sigma-Aldrich) and oven dried at 80 °C for 3 hours. The active catalyst CS_600_ was prepared by calcining the carbide slag at 600 °C for 2 hours. The composition of the CS_600_ catalyst was analyzed by XRD and FTIR analysis and the results were consistent with our published work [72]. The main phase was CaO (Figure 1) with reflections at 2θ = 32.2, 37.3, 53.8, 64.1, 67.4, 79.6, 88.5 (XRD and FTIR data is given in Supporting Information File 1).

The catalyst CS_600_ was used in the transesterification of soybean oil with methanol and the yield of fatty acid methyl ester mixture was 99% (Table 1, entry 1). Soybean oil was chosen as a model triglyceride composed of fatty acid residues as a mixture of palmitic acid (14.7%), stearic acid (3.6%), elaidic acid (24.5%), and linoleic acid (57.2%). Additional control experiments were performed with the original carbide slag (CS_25_) and commercial CaO, Ca(OH)_2_ and CaCO_3_ to confirm the main catalyst phase (CaO) as catalytically active species in the reaction and the results of the control experiments are summarized in Table 1. Commercial CaO catalyzed the transesterification with an 81% yield of fatty acid methyl esters (Table 1, entry 2). Ca(OH)_2_ showed weak transesterification activity and the yield of fatty acid methyl ester mixture was 26% (Table 1, entry 3). Similar results were obtained for the uncalcined carbide slag (CS_25_), the main phase of which is Ca(OH)_2_; the yield of fatty acid methyl esters was 28% (Table 1, entry 4). When using CaCO_3_, no formation of a mixture of fatty acid methyl esters was detected (Table 1, entry 5). Thus, the main phase of CaO in the CS_600_ catalyst was responsible for the catalytic activity, and minor components did not affect the reaction or were inert.

**Table 1:** Control experiments on the example of transesterification of soybean oil with methanol.^a^

Entry	Catalyst	Yield of fatty acid methyl ester mixture, %

1	СS_600_	99
2	CaO (commercial)	89
3	Ca(OH)_2_ (commercial)	26
4	CS_25_	28
5	CaCO_3_ (commercial)	NF^b^

^a^Reaction conditions: oil to alcohol molar ratio = 1:12; 1 wt % of catalyst, 65 °C, 2 h. ^b^NF: not formed.

Since the transesterification with a catalyst derived from carbide slag (CS_600_) and commercial (or obtained from another source) calcium oxide proceeds by the same mechanism with the formation of a new ester and an alcohol as byproduct, we evaluated the E-factor of the CaO catalyst production stage and compared it with the approach of obtaining CaO from a CaCO_3_ source. Thus, according to the literature, when producing 1 ton of CaO catalyst from CaCO_3_, nearly 0.89 tons of CO_2_ are emitted (Equation 1) [73]. The E-factor, defined as the ratio of the total weight of waste to the total weight of the product, is 0.89 for this process.


[1] $${\text{CaCO}}_{3}\to {\text{CaO + CO}}_{2}\text{; E-factor}=0.89$$

[2] $${\text{Ca(OH)}}_{\text{2}}\to \text{CaO}+{\text{H}}_{2}\text{O; E-factor = }0.32$$

On the other hand, to obtain a ton of CaO from carbide slag, 0.32 tons of water is released. The E-factor for this approach is 0.32, which is approximately 2.8 times less than the approach of obtaining CaO from a CaCO_3_ source (Equation 2). Thus, the proposed approach to obtaining a catalyst for transesterification reactions from carbide slag allows for waste utilization and is a greener alternative. Carbide slag is a ready-to-use source of CaO catalyst and does not require mining or additional processing.

**Scheme 1:**
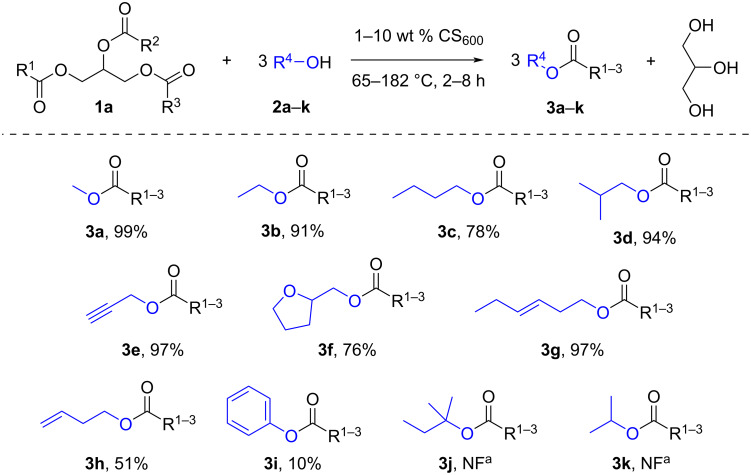
The transesterification of soybean oil with various alcohols in the presence of CS_600_ catalyst. Reaction conditions: oil to alcohol molar ratio = 1:12; **3a**: 1 wt % CS_600_, 65 °C, 2 h; **3b**: 5 wt % CS_600_, 78 °C, 3 h; **3c**: 5 wt % CS_600_, 120 °C, 5 h; **3d**: 10 wt % CS_600_, 108 °C, 4 h; **3e**: 1 wt % CS_600_, 115 °C, 4 h; **3f**: 10 wt % CS_600_, 178 °C, 5 h; **3g**: 5 wt % CS_600_, 157 °C, 4 h; **3h**: 1 wt % CS_600_, 115 °C, 4 h; **3i**: 10 wt % CS_600_, 182 °C, 8 h; **3j**: 10 wt % CS_600_, 105 °C, 4 h; **3k**: 10 wt % CS_600_, 83 °C, 4 h. ^a^NF: not formed.

Next, the CS_600_ catalyst was investigated in the transesterification of soybean oil with various alcohols. The overall results of the transesterification are presented in Scheme 1. All the reactions were carried out at the boiling point of the corresponding alcohol, using different CS_600_ loadings and reaction times.

The transesterification of soybean oil using 1 wt % CS_600_ proceeded in excellent yields with methanol and propargyl alcohol. The transesterification reaction with propargyl alcohol required an increase in reaction time to 4 hours, compared to 2 hours for methanol, for a more complete transesterification reaction. The corresponding esters **3a** and **3e** were obtained in 99% and 97% yields. For ethanol and *n*-butanol, the required amount of the catalyst was 5 wt %; the yields of esters **3b** and **3c** were 91% and 78% with reaction times of 3 and 5 hours, respectively. Similar results were obtained in the transesterification with tetrahydrofurfuryl alcohol: the yield of esters **3f** was 76% at a catalyst loading of 10 wt % for 5 hours. In the case of isobutanol, the best catalyst loading was 10 wt %, yielding a mixture of **3d** esters in 94% yield after 4 hours. An excellent reaction yield of 97% (**3g**) was obtained after 4 hours of reaction with *trans*-3-hexen-1-ol at a catalyst loading of 5 wt %. The yield of esters (**3h**) using 3-buten-1-ol at 1 wt % catalyst loading was moderate (51%) after 4 hours. Despite the weakly acidic properties, phenol was involved in the transesterification reaction, resulting in a mixture of esters (**3i**) in 10% yield, when the catalyst loading was 10 wt % and the reaction time was increased to 8 h. Increasing the reaction time to 24 h did not improve the reaction yield. The secondary (isopropanol, **2k**) and tertiary (*tert*-amyl alcohol, **2j**) alcohols were inert in the reaction; the formation of the corresponding ester mixtures **3j** and **3k** was not detected even in trace amounts. Alcohols **2j** and **2k** were additionally tested in the transesterification of low-molecular-weight esters using compounds **4a** and **4g** as examples. However, no transesterified products were observed. Thus, under these conditions, the inertness of alcohols **2j** and **2k** in the transesterification of both soybean oil and esters with less-hindered structures was probably due to their poor nucleophilic properties.

**Scheme 2:**
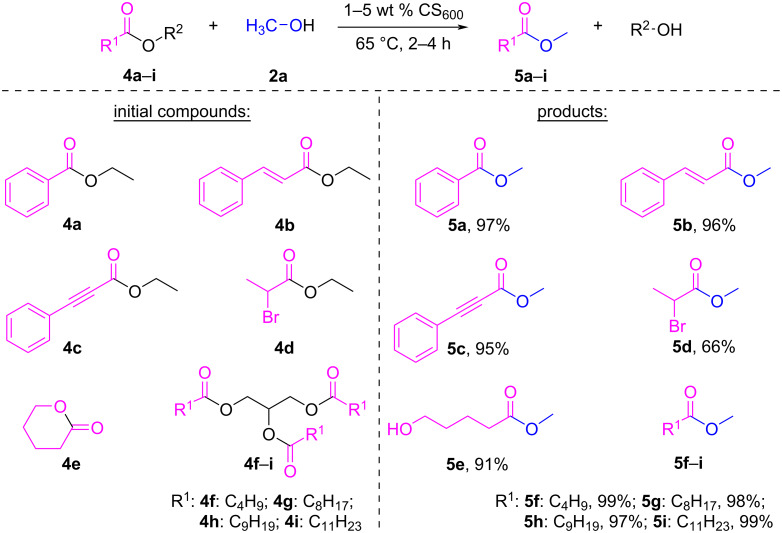
The transesterification of various esters with methanol in the presence of CS_600_ as catalyst. Reaction conditions: ester to alcohol molar ratio = 1:12, 65 °C; **5a**: 2 wt % CS_600_, 4 h; **5b**: 2 wt % CS_600_, 2 h; **5d**: 5 wt % CS_600_, 2 h; **5e**: 1 wt % CS_600_, 3 h; **5c** and **5f**–**i**: 1 wt % CS_600_, 2 h.

The CS_600_ catalyst was investigated in the transesterification of low-molecular-weight esters, lactones and triglycerides with medium hydrocarbon radical lengths (C_5_, C_9–10_ and C_12_) and the results are summarized in Scheme 2. All reactions were carried out at the boiling point of methanol (65 °C), with different CS_600_ loading and reaction times.

The transesterification of ethyl esters to the corresponding methyl esters proceeded in excellent yields for ethyl benzoate (**4a**), ethyl cinnamate (**4b**), and ethyl 3-phenylpropiolate (**4c**) at a CS_600_ catalyst loading of 2 wt % for 2 hours. The corresponding methyl esters **5a**, **5b**, and **5c** were obtained in 97%, 96%, and 95% yields. Low intensity signals of the initial ethyl esters were observed in the NMR spectra of the reaction mixtures. Remarkably, ethyl 2-bromopropanoate (**4d**) reacted less successfully in the transesterification and the yield of methyl ester **5d** within 2 h was 66%, even when the catalyst loading was increased to 5 wt %. The transesterification of δ-valerolactone (**4e**) successfully led to the opening of the ring structure of the lactone and the formation of methyl 5-hydroxypentanoate (**5e**) in 91% yield at a catalyst loading of 1 wt % and for 3 h. The transesterification of triglycerides obtained from the esterification of linear saturated C_5_, C_9_, C_10_, and C_12_ acids with glycerol proceeded in excellent yields in the range of 97–99% for 2 hours of reaction and required a low catalyst loading of 1 wt %. Methyl pentanoate (**5f**), methyl nonanoate (**5g**), methyl decanoate (**5h**), and methyl dodecanoate (**5i**) were obtained in 99%, 98%, 97%, and 99% yields, respectively.

**Scheme 3:**
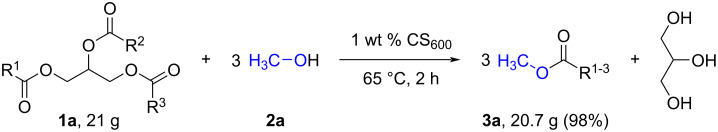
Gram-scale batch process for the transesterification of soybean oil with methanol. Reaction conditions: oil to alcohol molar ratio = 1:12, 1 wt % CS_600_, 65 °C, 2 h.

The transesterification of soybean oil (**1a**) with methanol (**2a**) was carried out as a gram-scale batch reaction using 21 g of soybean oil, 210 mg of catalyst (1 wt %), and 11.7 mL of methanol (Scheme 3). The temperature and reaction time conditions used were the same as in the previous experiments. A mixture of methyl esters of fatty acids **3a** was obtained in 98% yield (20.7 g). These results showed that the reaction could be easily scaled up in batch mode without changing the initial conditions.

**Figure 2:**
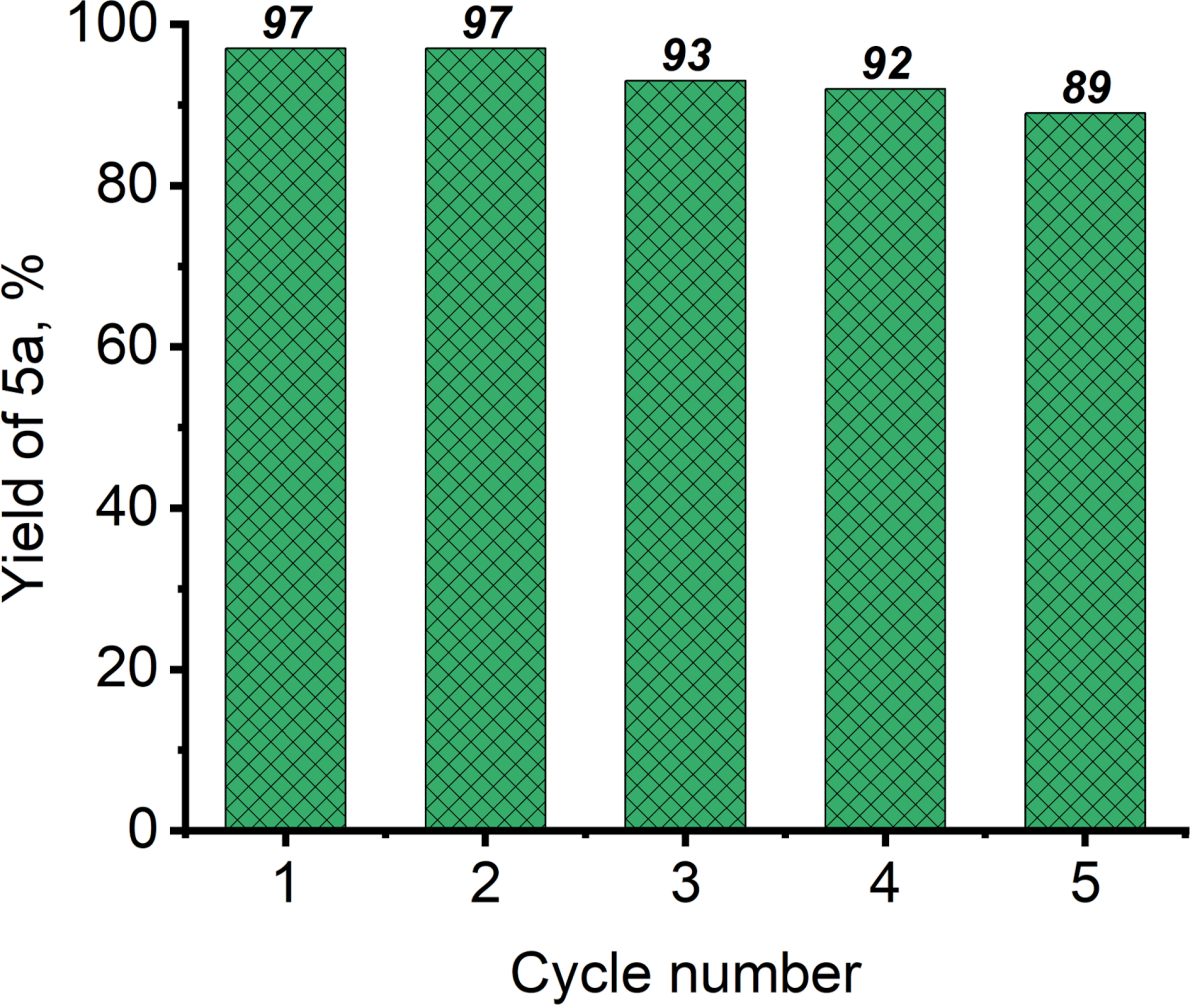
CS_600_ reusability test. Reaction conditions: 2 wt % CS_600_, MeOH/**4a** ratio is 12:1, 65 °C, 4 h.

The reusability of the CS_600_ catalyst was investigated in five reaction cycles using the transesterification of **4a** with methanol (**2a**). The detailed procedure and reagent loading are given in Supporting Information File 1 (section 2.7). After each reaction cycle, the catalyst was separated by centrifugation, then washed with methanol and hexane to remove organic impurities, dried at 80 °C for 30 min, then calcined at 600 °C for 2 h, and reused. The dependence of the yield of product **5a** on the reaction cycles is shown in Figure 2. The yield of compound **5a** in the 1st and 2nd reaction cycle was 97%, by the 3rd cycle it decreased to 93%. After the 5th reaction cycle, the yield of **5a** was 89%.

**Figure 3:**
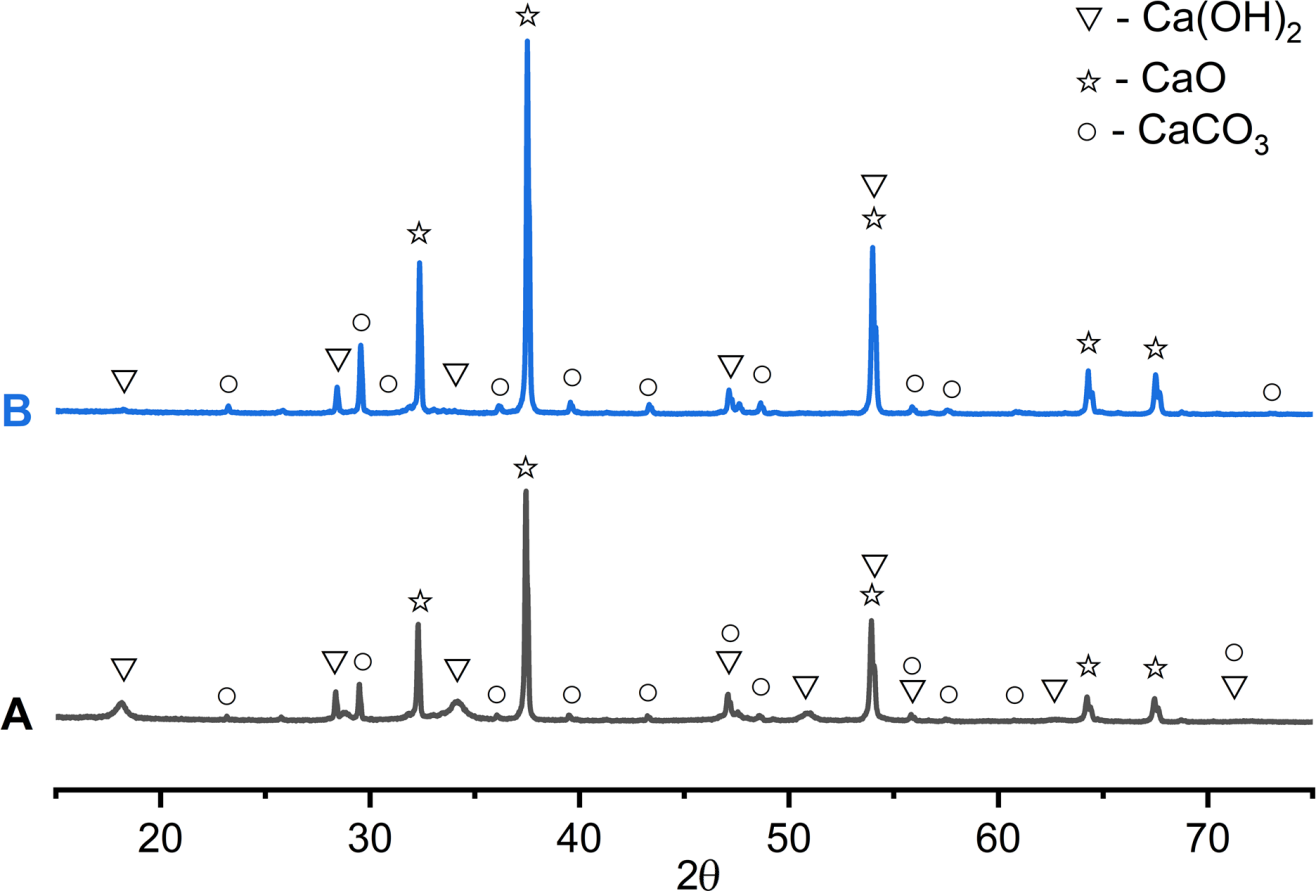
XRD patterns of the CS_600_ catalyst after the 1st reaction cycle: A) after washing with methanol and hexane and drying at 80 °C for 30 min; B) after calcination at 600 °C for 2 h.

After washing and drying the catalyst after the 1st reaction cycle, the XRD patterns (Figure 3A) showed the following main phases: 51.3 wt % of CaO (lime, ICDD 01-082-1690) with reflections at 2θ = 32.3, 37.4, 53.9, 64.2, 67.4; Ca(OH)_2_ (portlandite, ICDD 01-076-0570) in an amount of 34.2 wt % at 2θ = 18.1, 28.8, 34.1, 47.5, 50.9, 54.5, 56.7, 62.7, 64.7, 71.7; 8.6 wt % of CaCO_3_ (calcite, ICDD 01-083-4614) with reflections at 2θ = 23.2, 29.5, 36.1, 39.5, 43.2, 47.5, 48.6, 56.7, 57.5, 60.7, 64.7, 71.7. After calcining the catalyst at 600 °C for 2 hours, an increase in the amount of the CaO phase (with reflections at 2θ = 32.4, 37.5, 54.0, 64.3, 67.5) to 73.5 wt % was detected due to the decomposition of the Ca(OH)_2_ phase; the amount of the Ca(OH)_2_ phase after calcination was 2.4 wt %. The amount of the CaCO_3_ phase increased to 17.6 wt %, probably due to the sorption of carbon dioxide or the decomposition of organic impurities (Figure 3B). Thus, using **4a** as an example, the possibility of reusing the CS_600_ catalyst in transesterification was successfully demonstrated.

## Conclusion

Currently, the transesterification reaction of esters is a well-established procedure. The simple reaction results in a wide scope of the desired compounds. The unique nature of selected esters determines significant amounts of required products (e.g., biodiesel) and industrial-scale productions of valuable esters require huge amounts of catalysts. In this work, the used calcium carbide slag-based catalyst provided the targeted esters in high yields. In fact, a waste from industry was fully compatible with fine organic synthesis as an efficient catalyst.

In this work, the effectiveness of using a catalyst derived from carbide slag (CS_600_) for the transesterification of soybean oil with various alcohols was investigated. The results showed that the CS_600_ catalyst can provide high yields of fatty acid alkyl esters, ranging from 51% to 99%, depending on the type of alcohol and catalyst loading. Alcohols with additional functional groups allow to synthesize esters suitable for further modifications. The possibility of scaling up the reaction to 21 gram was investigated using the example of transesterification of soybean oil (**1a**) with methanol (**2a**). The CS_600_ catalyst was effective for transesterification of low-molecular-weight esters, medium-chain triglycerides and lactones, achieving yields from 66% to 99% at catalyst loadings from 1 wt % to 5 wt %. The CS_600_ catalyst was reused in 5 reaction cycles and the yield of ester **5a** after the 5th reaction cycle was 89%. These results highlight the promise of carbide slag as a sustainable and affordable catalyst for the production of various valuable esters. The use of industrial wastes not only contributes to reducing environmental impact, but also provides new opportunities for the development of sustainable synthetic methods in the chemical industry. Further research can be focused on optimizing the process and exploring the applicability of this catalyst in other organic synthetic reactions.

## Supporting Information

File 1 General information, experimental procedures, characterization data, and copies of NMR spectra.

## Data Availability

All data that supports the findings of this study is available in the published article and/or the supporting information of this article.
