# Supplementary material for: Calcium waste as a catalyst in the transesterification for demanding esters: scalability perspective
Source: Beilstein J Org Chem. 2025 Jul 28;21:1520–7. doi: 10.3762/bjoc.21.114 (PMC12320737; doi:10.3762/bjoc.21.114)
Supplement: File 1 — General information, experimental procedures, characterization data, and copies of NMR spectra. [file Beilstein_J_Org_Chem-21-1520-s001.pdf]

BEILSTEIN JOURNAL OF ORGANIC CHEMISTRY

Supporting Information

for

Calcium waste as a catalyst in the transesterification for demanding esters: scalability perspective

Anton N. Potorochenko and Konstantin S. Rodygin

Beilstein J. Org. Chem. **2025,** *21,* 1520–1527. doi:10.3762/bjoc.21.114

General information, experimental procedures, characterization data, and copies of NMR spectra

License and Terms: This is a supporting information file under the terms of the Creative Commons Attribution License (https://creativecommons.org/licenses/by/4.0). Please note that the reuse, redistribution and reproduction in particular requires that the author(s) and source are credited and that individual graphics may be subject to special legal provisions.
The license is subject to the *Beilstein Journal of Organic Chemistry* terms and conditions: (https://www.beilstein-journals.org/bjoc/terms)

Table of contents

1. General information **S2**

2. General procedures and characterization data **S3**

2.1 General procedure for preparation of CS600 catalyst *S3*

*2.2 General procedure for the synthesis of esters **3a–k** by transesterification reaction of SBO* *S3*

2.2.1 Fatty acid methyl ester **3a** S3
2.2.2 Fatty acid ethyl ester **3b** S4
2.2.3 Fatty acid *n*-butyl ester **3c** S4
2.2.4 Fatty acid isobutyl ester **3d** S4
2.2.5 Fatty acid 2-propynyl ester **3e** S4
2.2.6 Fatty acid tetrahydrofurfuryl ester **3f** S4
2.2.7 Fatty acid 3-hexenyl ester **3g** S4
2.2.8 Fatty acid 3-butenyl ester **3h** S4

*2.3 Procedure for the synthesis of δ-valerolactone (**4e**)* *S5*

*2.4 General procedure for the synthesis of triglycerides **4f–i*** *S5*

2.4.1 Propane-1,2,3-triyl tripentanoate (**4f**) S5
2.4.2 Propane-1,2,3-triyl trinonanoate (**4g**) S5
2.4.3 Propane-1,2,3-triyl tris(decanoate) (**4h**) S5
2.4.4 Propane-1,2,3-triyl tridodecanoate (**4i**) S5

*2.5 General procedure for the synthesis of methyl esters **5a–i** by transesterification reaction of various esters* *S6*

2.5.1 Methyl benzoate (**5a**) S6
2.5.2 Methyl cinnamate (**5b**) S6
2.5.3 Methyl 3-phenylpropiolate (**5c**) S6
2.5.4 Methyl 2-bromopropanoate (**5d**) S6
2.5.5 Methyl 5-hydroxypentanoate (**5e**) S6
2.5.6 Methyl pentanoate (**5f**) S6
2.5.7 Methyl nonanoate (**5g**) S6
2.5.8 Methyl decanoate (**5h**) S6
2.5.9 Methyl dodecanoate (**5i**) S6

2.6 General procedure for the gram-scale batch process for the transesterification of soybean oil with methanol *S7*

*2.7 Procedure for reusing the CS_{600} catalyst (using the example of transesterification of compound **4a**)* *S7*

3. NMR spectra of compounds **S8**

4. Characterization of the catalyst CS_{600} **S30**

5. References **S33**

1. General information

Calcium carbide (granulated, technical grade, ≥75% (gas-volumetric)) was purchased from Sigma-Aldrich. CaO, $Ca(OH)_2$, and $CaCO_3$ were purchased from VECTON JSC and used without further purification. Chloroform (analytical grade) was purchased from Chemical Line Co. Ltd and used without additional purification. Hexane (chemically pure grade) and EtOAc (chemically pure grade) were purchased from VECTON JSC and used without additional purification. Methanol (**2a**) and ethanol (**2b**) were purified by using standard procedure and was stored over a type 3 Å molecular sieves [1]. Butanol (**2c**), isobutanol (**2d**), propargyl alcohol (**2e**), tetrahydrofurfuryl alcohol (**2f**), *trans*-3-hexen-1-ol (**2g**), 3-buten-1-ol (**2h**), phenol (**2i**), *tert*-amyl alcohol (**2j**), and isopropanol (**2k**) were purchased from Sigma-Aldrich, abcr GmbH, Acros Organics and used without additional purification. Soybean oil (SBO) was purchased from the local market (produced by Ottogi Co., Ltd, Republic of Korea) and used without additional purification. Glycerin (VG) to USP/EP purification standard (99.9% purity) was purchased from A&M at a local market. Ethyl benzoate (**4a**), ethyl cinnamate (**4b**), ethyl 3-phenylpropiolate (**4c**), ethyl 2-bromopropanoate (**4d**), and cyclopentanone were purchased from Sigma-Aldrich, abcr GmbH, Acros Organics and used without additional purification. Pentanoic acid (analytical grade) was purchased from VECTON JSC and used without additional purification. Nonanoic acid, decanoic acid, and dodecanoic acid were purchased from Acros Organics and used without additional purification.

XRD patterns were recorded by using a Bruker "D2 Phaser" powder diffractometer operating with X-ray tube radiation $CuK\alpha_{1+2}$, wavelengths $\lambda_{CuK\alpha1}$ = 1.54059 Å and $\lambda_{CuK\alpha2}$ = 1.54443 Å, tube operation mode 30 kV/10 mA, position-sensitive detector, reflection geometry, Bragg-Brentano focusing scheme, sample rotation speed 20 rpm, diffraction angle interval 2θ = 5(8)-90°, scanning step 0.02°, exposure at a point 0.7 seconds, T = 25 °C, air atmosphere.

FTIR spectra were recorded in KBr pellets in the wavenumber range 400–4000 cm^{-1} with a resolution of 1 cm^{-1} using a Bruker IFS 66 spectrometer.

NMR spectra were recorded on a Bruker Avance III spectrometer 400 MHz (400 MHz for ^1H; 101 MHz for ^{13}C). Chemical shifts δ are reported in ppm using residual protons of $CDCl_3$ as internal standards (^1H, δ = 7.26; ^{13}C, δ = 77.16). The yields of esters were calculated by ^1H NMR measurements of the extracted and evaporated residues with benzene (at 7.36 ppm) or nitromethane (at 4.33 ppm) as an internal standard. The standard was chosen depending on the signal overlapping in the NMR spectrum: for compound **3i**, nitromethane, for the other compounds, benzene. The NMR yields of esters were determined by following equations (1–3):

$$N_2 = N_1 \cdot \frac{I_2 \cdot n_1}{I_1 \cdot n_2} \quad (1)$$

$$N_2' = \frac{m_{mix} \cdot N_2}{m_{NMR}} \quad (2)$$

$$\text{Yield (\%)} = \frac{N_2'}{N_{ester}} * 100\% \quad (3)$$

where I_1 and I_2 are the NMR integral values of benzene (or nitromethane) and product peaks, respectively; n_1 and n_1 are coefficients corresponding to the number of protons for the signals of internal standard and ester, respectively; N_1 and N_2 are mole quantities of benzene (or nitromethane) and product in ^1H NMR, respectively; N_2' is the mole quantities of product in the isolated mixture; m_{mix} and m_{NMR} are the mass of the isolated mixture and the mass used for NMR analysis, respectively; N_{ester} is the theoretical amount of ester.

The yield of methyl esters obtained in the transesterification of ethyl esters with methanol was calculated using the equations 4–6:

$$r_{a/b} = \frac{I_a \cdot n_b}{I_b \cdot n_a} \quad (4)$$

$$N'_a = \frac{m_{mix}}{M_a + \frac{M_b}{r_{a/b}}} \quad (5)$$

$$\text{Yield (\%)} = \frac{N'_a}{N_{ester}} * 100\% \quad (6)$$

where $r_{a/b}$ is the molar ratio of the product to the initial ester; I_a and I_b are the NMR integral values of the product peak and the peak of the initial ester, respectively; n_a and n_b are coefficients corresponding to the number of protons for the signals of product and initial ester, respectively; M_a and M_b are the molecular weights of the product and the initial ester, respectively; N_a' is the mole quantities of product in the isolated mixture; m_{mix} is the mass of the isolated mixture; N_{ester} is the theoretical amount of product.
Column chromatography was performed using Merck silica gel 60 (60–200 mesh) that was preliminarily neutralized with Et_3N. Pre-coated TLC sheets ALUGRAM Xtra SIL G/UV_{254} were used for thin-layer chromatography; a solution of 5% $KMnO_4$ (universal indicator), ethanol solution of bromocresol purple (acid indicator) were used for visualization.
GC–MS measurements were performed using a QP2010 Ultra Shimadzu system equipped with an Agilent Technologies HP-5ms column (0.25 μm, 60 m × 0.32 mm), carrier gas was He (high purity, grade 6.0). The component composition of SBO was determined by GC–MS technique of the corresponding methyl esters and was a mixture of palmitic acid (14.7%), stearic acid (3.6%), elaidic acid (24.5%), and linoleic acid (57.2%).

2. General procedures and characterization data

2.1 General procedure for preparation of CS600 catalyst

The carbide slag (CS) was obtained by hydrolysis of calcium carbide and dried in an oven at 80 °C for 3 hours. The dried CS was ground in a porcelain mortar and stored under argon. CS_{600} catalyst was prepared by calcination for 2 hours at 600 °C. The catalyst samples were then transferred to a storage container, cooled with a flow of argon, and stored in an argon atmosphere.

2.2 General procedure for the synthesis of esters 3a–k by transesterification reaction of SBO

The reaction was carried out in a flask under reflux. SBO (1.5 mmol, 1308 mg), alcohol (18 mmol) and catalyst (1–10 wt %) were added to the flask, then the reaction mixture was heated for a certain time with stirring at the boiling point of the alcohol under reflux. After completion of the transesterification process, the reaction mixture was centrifuged (3000 rpm, 3 min) and separated from the catalyst. The resulting mixture was dissolved in 5 mL of $CHCl_3$ and washed with water (2 × 10 mL) and brine (1 × 10 mL). The organic phase was dried over Na_2SO_4, filtered and concentrated. The mass of the isolated mixture was weighed and the ester yield (%) was calculated using ^{1}H NMR spectroscopy. The obtained crude products were purified using column chromatography with hexane/ethyl acetate as the eluent.

2.2.1 Fatty acid methyl ester 3a

Light yellow oil; yield 99%; ^{1}H NMR (400 MHz, $CDCl_3$) δ 5.44 – 5.26 (m, 1H), **3.65 (s, 3H)**, 2.83 – 2.71 (m), 2.29 (t, J = 7.5 Hz), 2.10 - 1.94 (m), 1.69 – 1.54 (m), 1.39 – 1.19 (m), 0.97 (t, J = 7.5 Hz), 0.91 – 0.84 (m); ^{13}C NMR (101 MHz, $CDCl_3$) δ 174.43, 132.08, 130.34, 130.17, 130.13, 129.88, 128.41, 128.19, 128.05, 127.87, 127.26, 77.48, 77.16, 76.84, **51.54**, 34.23, 32.67, 32.06, 32.04, 31.66, 29.90, 29.82, 29.72, 29.66, 29.59, 29.48, 29.46, 29.39, 29.29, 29.26, 29.23, 27.34, 27.32, 27.30, 25.77, 25.08, 22.81, 22.71, 20.68, 14.39, 14.37, 14.23, 14.19.

2.2.2 Fatty acid ethyl ester 3b

Light yellow oil; yield 91%; ^{1}H NMR (400 MHz, $CDCl_3$) δ 5.42 – 5.24 (m), **4.11 (q, *J* = 7.1 Hz, 2H)**, 2.82 – 2.71 (m), 2.27 (t, *J* = 7.5 Hz), 2.09 – 1.94 (m), 1.65 – 1.55 (m), **1.39 – 1.18 (m, 3H**+nH**)**, 0.96 (t, *J* = 7.5 Hz), 0.91 – 0.83 (m); ^{13}C NMR (101 MHz, $CDCl_3$) δ 173.94, 132.04, 130.36, 130.30, 130.14, 130.09, 129.86, 128.39, 128.36, 128.16, 128.03, 127.85, 127.24, **60.23**, 34.49, 32.05, 32.03, 31.65, 29.89, 29.81, 29.78, 29.71, 29.65, 29.59, 29.47, 29.44, 29.39, 29.29, 29.24, 29.23, 27.32, 27.31, 27.28, 25.75, 25.09, 22.80, 22.69, 20.67, **14.36**, 14.21, 14.16.

2.2.3 Fatty acid n-butyl ester 3c

Yellow oil; yield 78%; ^{1}H NMR (400 MHz, $CDCl_3$) δ 5.45 – 5.23 (m), **4.06 (t, *J* = 6.7 Hz, 2H)**, 2.84 – 2.70 (m), 2.28 (t, *J* = 7.5 Hz), 2.10 – 1.95 (m), **1.67 – 1.54 (m, 2H**+nH**)**), **1.45 – 1.17 (m, 2H**+nH**)**, 0.99 – 0.94 (m), **0.93 (t, *J* = 7.4 Hz, 3H)**, 0.91 – 0.85 (m, 1H); ^{13}C NMR (101 MHz, $CDCl_3$) δ 174.13, 174.08, 132.09, 130.40, 130.34, 130.18, 130.13, 129.89, 128.42, 128.19, 128.06, 127.87, **64.23**, 34.53, 32.05, 31.67, **30.87**, 29.91, 29.83, 29.79, 29.74, 29.66, 29.61, 29.49, 29.46, 29.41, 29.30, 29.27, 29.25, 27.35, 27.33, 27.31, 25.77, 25.67, 25.15, 22.82, 22.71, 20.69, **19.29**, 14.39, 14.23, 14.19, **13.83.**

2.2.4 Fatty acid isobutyl ester 3d

Yellow oil; yield 94%; ^{1}H NMR (400 MHz, $CDCl_3$) δ 5.43 – 5.26 (m), **3.84 (d, *J* = 6.7 Hz, 2H)**, 2.83 – 2.71 (m), 2.30 (t, *J* = 7.5 Hz), 2.10 – 1.98 (m), **1.97 – 1.85 (m, *J* = 6.7 Hz, 1H)**, 1.67 – 1.56 (m), 1.38 – 1.21 (m), 0.97 (t, *J* = 7.6 Hz), **0.92 (d, *J* = 6.7 Hz, 6H)**, 0.90 – 0.85 (m); ^{13}C NMR (101 MHz, $CDCl_3$) δ 174.07, 174.03, 132.07, 130.39, 130.32, 130.16, 130.12, 129.88, 128.39, 128.18, 128.05, 127.87, 127.26, **70.49**, 34.52, 32.06, 32.04, 31.66, 29.90, 29.82, 29.79, 29.73, 29.66, 29.60, 29.48, 29.45, 29.40, 29.30, 29.28, 29.25, **27.88**, 27.34, 27.32, 27.30, 25.77, 25.17, 22.81, 22.70, **19.22**, 14.39, 14.22, 14.18.

2.2.5 Fatty acid 2-propynyl ester 3e

Yellow-orange oil; yield 97%; ^{1}H NMR (400 MHz, $CDCl_3$) δ 5.45 – 5.24 (m), **4.67 (d, *J* = 2.4 Hz, 2H)**, 2.82 – 2.69 (m), **2.45 (t, *J* = 2.4 Hz, 1H)**, 2.34 (t, *J* = 7.5 Hz), 2.10 – 1.94 (m), 1.69 – 1.57 (m), 1.39 – 1.19 (m), 0.97 (t, *J* = 7.5 Hz), 0.91 – 0.83 (m); ^{13}C NMR (101 MHz, $CDCl_3$) δ 173.09, 173.05, 132.08, 130.34, 130.15, 129.86, 128.42, 128.38, 128.20, 128.04, 127.89, 127.26, **77.95**, **74.80**, **51.86**, 34.11, 32.06, 32.04, 31.66, 29.90, 29.80, 29.70, 29.65, 29.56, 29.48, 29.45, 29.35, 29.24, 29.20, 29.16, 27.34, 27.32, 27.29, 25.77, 25.67 24.93, 22.81, 22.70, 20.68, 14.39, 14.23, 14.19.

2.2.6 Fatty acid tetrahydrofurfuryl ester 3f

Orange oil; yield 76%; ^{1}H NMR (400 MHz, $CDCl_3$) δ 5.39 – 5.21 (m, 1H), **4.14 – 4.02 (m, 2H)**, **4.00 – 3.91 (m, 1H), 3.89 – 3.80 (m, 1H), 3.78 – 3.71 (m, 1H)**, 2.79 – 2.67 (m), 2.30 (t, *J* = 7.5 Hz), **2.06 – 1.91 (m, 1H**+nH**)**, **1.91 – 1.79 (m, 2H)**, **1.63 – 1.50 (m, 1H**+nH**)**, 1.36 – 1.17 (m), 0.93 (t, *J* = 7.5 Hz), 0.89 – 0.79 (m); ^{13}C NMR (101 MHz, $CDCl_3$) δ 173.77, 173.73, 131.92, 130.24, 130.17, 130.02, 129.98, 129.75, 128.28, 128.26, 128.06, 127.94, 127.75, 127.15, **76.58**, **68.43**, **66.31**, 34.21, 31.96, 31.94, 31.55, 29.80, 29.72, 29.69, 29.62, 29.56, 29.49, 29.38, 29.35, 29.29, 29.19, 29.17, 29.13, **28.04**, 27.38, 27.23, 27.19, **25.69**, 25.66, 25.56, 24.95, 22.71, 22.60, 20.57, 14.28, 14.12, 14.08.

2.2.7 Fatty acid 3-hexenyl ester 3g

Colorless oil; yield 97%; ^{1}H NMR (400 MHz, $CDCl_3$) δ **5.55 (dt, *J* = 13.9, 6.3 Hz, 1H)**, **5.42 – 5.28 (m, 1H**+nH**)**, **4.07 (t, *J* = 6.9 Hz, 2H)**, 2.84 – 2.71 (m), **2.35 – 2.24 (m, 2H**+nH**)**, **2.10 – 1.95 (m, 2H**+nH**)**, 1.67 – 1.53 (m), 1.39 – 1.21 (m), **0.96 (t, *J* = 7.5 Hz, 3H**+nH**)**, 0.92 – 0.84 (m); ^{13}C NMR (101 MHz, $CDCl_3$) δ 173.99, 173.95, **135.12**, 132.08, 130.34, 130.17, 130.12, 129.88, 128.39, 128.18, 128.05, 127.86, 127.26, **124.26**, **64.02**, 34.50, **32.13**, 32.06, 32.04, 31.67, 29.91, 29.83, 29.79, 29.74, 29.66, 29.60, 29.49, 29.46, 29.41, 29.31, 29.26, 27.34, 27.31, **25.76**, 25.75, 25.14, 22.82, 22.71, 14.24, 14.19, **13.86**.

2.2.8 Fatty acid 3-butenyl ester 3h

Light yellow oil; yield 51%; ^{1}H NMR (400 MHz, $CDCl_3$) δ **5.78 (ddt, *J* = 17.0, 10.2, 6.7 Hz, 1H)**, 5.44 – 5.26 (m), **5.10 (dd, *J* = 23.7, 6.1 Hz, 2H)**, **4.12 (t, *J* = 6.7 Hz, 2H)**, 2.83 – 2.73 (m), **2.38 (q, *J* = 6.7 Hz, 2H)**, 2.29 (t, *J* = 7.5 Hz), 2.10 – 1.96 (m), 1.67 – 1.56 (m), 1.40 – 1.21 (m), 0.97 (t, *J* = 7.5 Hz), 0.93 – 0.83 (m); ^{13}C NMR (101 MHz, $CDCl_3$) δ 173.93, **134.22**, 132.08, 130.34, 130.17, 130.13, 129.88, 128.42, 128.39, 128.18, 128.05,

127.87, 127.26, **117.26**, **63.38**, 34.45, **33.26**, 32.06, 32.04, 31.66, 29.90, 29.83, 29.79, 29.73, 29.66, 29.60, 29.48, 29.46, 29.40, 29.30, 29.24, 27.34, 27.30, 25.77, 25.67, 25.11, 22.82, 22.71, 14.40, 14.24, 14.19.

2.3 Procedure for the synthesis of δ-valerolactone (4e)

To cyclopentanone (12 mmol, 1.01 g) in DCM (20 mL) was added *m*-CPBA (18 mmol, 3.11 g) portion-wise and the mixture was stirred at room temperature for 48 h. After the reaction, the organic layer was washed with 10% $Na_2S_2O_3$ solution (2 × 20 mL), $NaHCO_3$ solution (2 × 20 mL), brine, dried over Na_2SO_4, filtered and concentrated. The obtained crude products were purified using column chromatography with hexane/ethyl acetate as the eluent.

2.3.1 δ-Valerolactone (4e)

Colorless oil; 1.13 g (94%); ^{1}H NMR (400 MHz, $CDCl_3$) δ 4.31 (t, J = 5.5 Hz, 2H), 2.52 (t, J = 6.9 Hz, 2H), 1.94 – 1.74 (m, 4H); ^{13}C NMR (101 MHz, $CDCl_3$) δ 171.44, 69.51, 29.90, 22.38, 19.17; cf. lit. data [2].

2.4 General procedure for the synthesis of triglycerides 4f–i

The synthesis of triglycerides **4f–i** was carried out by a modernized procedure [3] using CS_{600} instead of calcium oxide. To a 5 mL flask containing glycerol (184 mg, 2 mmol) and equipped with a condenser were added the carboxylic acid (8 mmol) and CS_{600} (1.7 mg, 0.03 mmol). The mixture was heated under partial vacuum (5 mbar) until the carboxylic acid boiled and held for 22 h. The temperature of the water in the condenser was approximately 35 °C to maintain gentle boiling of the carboxylic acid and to accelerate the removal of water under vacuum. For compounds **4f** and **4g**: the reaction mixture was cooled and dissolved in ethyl acetate. This solution was washed with 10% NaOH solution, brine, dried over Na_2SO_4, filtered and concentrated. The obtained crude products were purified using column chromatography with hexane/ethyl acetate as the eluent. For compounds **4h** and **4i**: the reaction mixture was cooled to room temperature and the residue was dissolved in hot ethanol (95%, 7 mL). This solution was filtered and cooled in an ice bath at 0–5 °C for 2 h. The triglycerides crystallized as a white solid, which was filtered and washed with cold ethanol (95%, 5 mL).

2.4.1 Propane-1,2,3-triyl tripentanoate (4f)

Yellow oil; 544 mg (79%); ^{1}H NMR (400 MHz, $CDCl_3$) δ 5.30– 5.21 (m, 1H), 4.37 – 4.05 (m, 4H), 2.36 – 2.27 (m, 6H), 1.65 – 1.54 (m, 6H), 1.40 – 1.28 (m, 6H), 0.90 (t, J = 7.3 Hz, 9H); ^{13}C NMR (101 MHz, $CDCl_3$) δ 173.39, 172.98, 69.00, 62.21, 34.03, 33.87, 27.05, 27.02, 22.30, 22.26, 13.76.

2.4.2 Propane-1,2,3-triyl trinonanoate (4g)

Colorless oil; 741.2 mg (72%); ^{1}H NMR (400 MHz, $CDCl_3$) δ 5.30 – 5.21 (m, 1H), 4.32 – 4.08 (m, 4H), 2.33 – 2.25 (m, 6H), 1.65 – 1.55 (m, 6H), 1.31 – 1.24 (m, 30H), 0.87 (t, J = 6.8 Hz, 9H); ^{13}C NMR (101 MHz, $CDCl_3$) δ 173.40, 172.98, 69.01, 62.22, 34.34, 34.17, 31.93, 29.35, 29.33, 29.26, 29.24, 29.19, 25.02, 24.98, 22.76, 14.19; cf. lit. data [4].

2.4.3 Propane-1,2,3-triyl tris(decanoate) (4h)

White powder; 832.3 mg (75%); ^{1}H NMR (400 MHz, $CDCl_3$) δ 5.30 – 5.22 (m, 1H), 4.33 – 4.10 (m, 4H), 2.36 – 2.23 (m, 6H), 1.68 – 1.53 (m, 6H), 1.37 – 1.16 (m, 36H), 0.87 (t, J = 6.7 Hz, 9H); ^{13}C NMR (101 MHz, $CDCl_3$) δ 173.43, 173.02, 69.03, 62.25, 34.37, 34.20, 32.00, 29.58, 29.56, 29.42, 29.40, 29.25, 29.22, 25.05, 25.01, 22.80, 14.22.

2.4.4 Propane-1,2,3-triyl tridodecanoate (4i)

White powder; 1131 mg (88%); ^{1}H NMR (400 MHz, $CDCl_3$) δ 5.34 – 5.19 (m, 1H), 4.32 – 4.11 (m, 4H), 2.39 – 2.24 (m, 6H), 1.68 – 1.54 (m, 6H), 1.36 – 1.17 (m, 48H), 0.88 (t, J = 6.8 Hz, 9H); ^{13}C NMR (101 MHz, $CDCl_3$) δ 173.43, 173.02, 69.02, 62.25, 34.37, 34.25, 34.20, 32.06, 29.76, 29.64, 29.62, 29.48, 29.44, 29.41, 29.26, 29.23, 25.05, 25.01, 22.82, 14.24.

2.5 General procedure for the synthesis of methyl esters 5a–i by transesterification reaction of various esters

The reaction was carried out in a flask under reflux. Ester (1 mmol), methanol (12 mmol, 385 mg, 486 µL) and catalyst (1–5 wt %) were added to the flask, then the reaction mixture was heated for a certain time with stirring at 65 °C. After completion of the transesterification process, the reaction mixture was centrifuged (3000 rpm, 3 min) and separated from the catalyst. The resulting mixture was dissolved in 5 mL of $CHCl_3$ and washed with water (2 × 10 mL) and brine (1 × 10 mL). The organic phase was dried over Na_2SO_4, filtered and concentrated. The mass of the isolated mixture was weighed and the ester yield (%) was calculated using ^{1}H NMR spectroscopy. The obtained crude products were purified using column chromatography with hexane/ethyl acetate as the eluent.

2.5.1 Methyl benzoate (5a)

Colorless oil; yield 97%; ^{1}H NMR (400 MHz, $CDCl_3$) δ 8.04 (d, J = 7.2 Hz, 2H), 7.57 – 7.50 (m, 1H), 7.46 – 7.39 (m, 2H), 3.91 (s, 3H); ^{13}C NMR (101 MHz, $CDCl_3$) δ 167.16, 132.97, 130.26, 129.64, 128.42, 52.13; cf. lit. data [5].

2.5.2 Methyl cinnamate (5b)

White powder; yield 96%; ^{1}H NMR (400 MHz, $CDCl_3$) δ 7.70 (d, J = 16.0 Hz, 1H), 7.55 – 7.46 (m, 2H), 7.42 – 7.33 (m, 3H), 6.44 (d, J = 16.0 Hz, 1H), 3.80 (s, 3H); ^{13}C NMR (101 MHz, $CDCl_3$) δ 167.50, 144.96, 134.52, 130.39, 128.99, 128.17, 117.94, 51.77; cf. lit. data [6].

2.5.3 Methyl 3-phenylpropiolate (5c)

Light yellow oil, yield 95%; ^{1}H NMR (400 MHz, $CDCl_3$) δ 7.61 – 7.54 (m, 2H), 7.44 (t, J = 7.4 Hz, 1H), 7.36 (t, J = 7.5 Hz, 2H), 3.83 (s, 3H); ^{13}C NMR (101 MHz, $CDCl_3$) δ 154.56, 133.09, 130.79, 128.69, 119.63, 86.58, 80.47, 52.87; cf. lit. data [7].

2.5.4 Methyl 2-bromopropanoate (5d)

Colorless oil, yield 66%; ^{1}H NMR (400 MHz, $CDCl_3$) δ 4.38 (q, J = 6.9 Hz, 1H), 3.78 (s, 3H), 1.82 (d, J = 6.9 Hz, 3H); ^{13}C NMR (101 MHz, $CDCl_3$) δ 170.85, 53.10, 39.88, 21.81; cf. lit. data [8].

2.5.5 Methyl 5-hydroxypentanoate (5e)

Light yellow oil; yield 91%; ^{1}H NMR (400 MHz, $CDCl_3$) δ 3.64 (s, 3H), 3.61 (t, J = 6.3 Hz, 2H), 2.33 (t, J = 7.3 Hz, 2H), 1.99 (s, 1H), 1.75 – 1.51 (m, 4H); ^{13}C NMR (101 MHz, $CDCl_3$) δ 174.33, 62.24, 51.65, 33.74, 32.10, 21.19; cf. lit. data [9].

2.5.6 Methyl pentanoate (5f)

Light yellow oil; yield 99%; ^{1}H NMR (400 MHz, $CDCl_3$) δ 3.65 (s, 3H), 2.29 (t, J = 7.5 Hz, 2H), 1.65 – 1.52 (m, 2H), 1.39 – 1.26 (m, 2H), 0.90 (t, J = 7.3 Hz, 3H); ^{13}C NMR (101 MHz, $CDCl_3$) δ 174.41, 51.51, 33.93, 27.15, 22.38, 13.79; cf. lit. data [10].

2.5.7 Methyl nonanoate (5g)

Colorless oil; yield 98%; ^{1}H NMR (400 MHz, $CDCl_3$) δ 3.65 (s, 3H), 2.28 (t, J = 7.6 Hz, 2H), 1.66 – 1.55 (m, 2H), 1.33 – 1.20 (m, 10H), 0.86 (t, J = 6.8 Hz, 3H); ^{13}C NMR (101 MHz, $CDCl_3$) δ 174.45, 51.52, 34.24, 31.93, 29.34, 29.28, 29.23, 25.09, 22.76, 14.18; cf. lit. data [11].

2.5.8 Methyl decanoate (5h)

Colorless oil; yield 97%; ^{1}H NMR (400 MHz, $CDCl_3$) δ 3.65 (s, 3H), 2.28 (t, J = 7.5 Hz, 2H), 1.65 – 1.55 (m, 2H), 1.34 – 1.20 (m, 12H), 0.86 (t, J = 6.8 Hz, 3H); ^{13}C NMR (101 MHz, $CDCl_3$) δ 174.44, 51.52, 34.24, 31.99, 29.53, 29.38, 29.28, 25.09, 22.78, 14.20; cf. lit. data [12].

2.5.9 Methyl dodecanoate (5i)

Colorless oil; yield 99%; ^{1}H NMR (400 MHz, $CDCl_3$) δ 3.65 (s, 3H), 2.29 (t, J = 7.5 Hz, 2H), 1.66 – 1.56 (m, 2H), 1.35 – 1.19 (m, 16H), 0.87 (t, J = 6.7 Hz, 3H); ^{13}C NMR (101 MHz, $CDCl_3$) δ 174.46, 51.54, 34.26, 32.04, 29.73, 29.59, 29.47, 29.40, 29.30, 25.10, 22.82, 14.23; cf. lit. data [13].

2.6 General procedure for the gram-scale batch process for the transesterification of soybean oil with methanol

The reaction was carried out in a flask under reflux. SBO **1a** (0.024 mol, 21 g), MeOH (**2a**, 0.29 mol, 11.7 mL) and catalyst CS_{600} (1 wt %, 0.21 g) were added to the flask, then the reaction mixture was heated at 65 °C for 2 hours. After completion of the transesterification process, the reaction mixture was centrifuged (3000 rpm, 3 min) and separated from the catalyst. The resulting mixture was dissolved in 25 mL of $CHCl_3$ and washed with water (2 × 30 mL) and brine (1 × 30 mL). The organic phase was dried over Na_2SO_4, filtered and concentrated. The isolated mixture was weighed and the ester yield (%) was calculated using ^{1}H NMR spectroscopy.

2.7 Procedure for reusing the CS_{600} catalyst (using the example of transesterification of compound 4a)

Before repeating the cycle, the catalyst was separated from the reaction mixture by centrifugation, washed with hexane (5 mL) and methanol (5 mL). The catalyst was transferred to a porcelain crucible and dried in an oven at 80 °C for 30 minutes. The crucible was then transferred to a muffle furnace preheated to 600 °C, and the catalyst was calcined for 2 hours. After calcination, the catalyst was immediately transferred to a storage container, weighed, and stored under argon until cooled to room temperature, and then reused (according to reagent loads in Table S1).

Table S1. Loading of reagents for reuse of the catalyst in transesterification **4a**

	1 cycle	2 cycle	3 cycle	4 cycle	5 cycle
Compound **4a**, g	4.5	3.5	2.5	2	1.75
MeOH **2a**, mL	14.5	11.3	8.1	6.5	5.6
CS_{600}, g	0.09	0.07	0.05	0.04	0.035

3. NMR spectra of compounds

3.1 Fatty acid methyl ester 3a

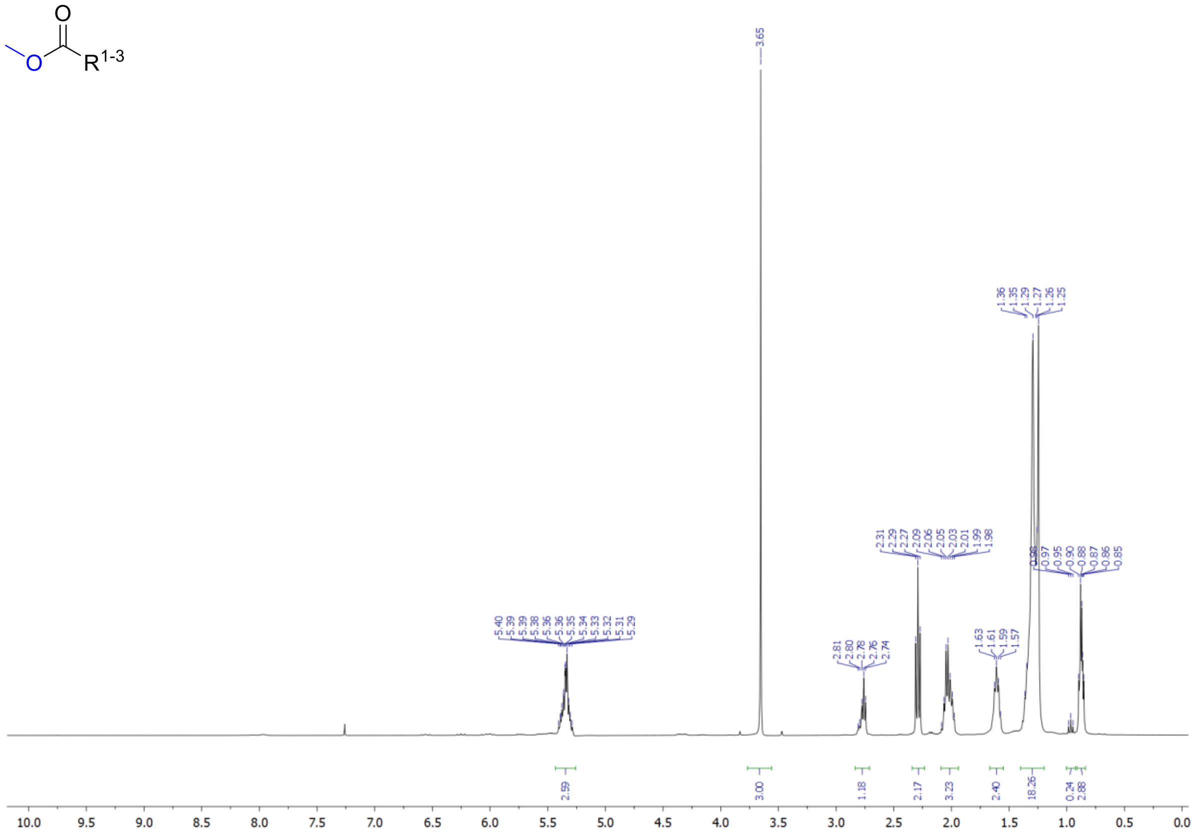

Figure S1. ^{1}H NMR spectrum (400 MHz, $CDCl_3$) of ester mixture **3a**

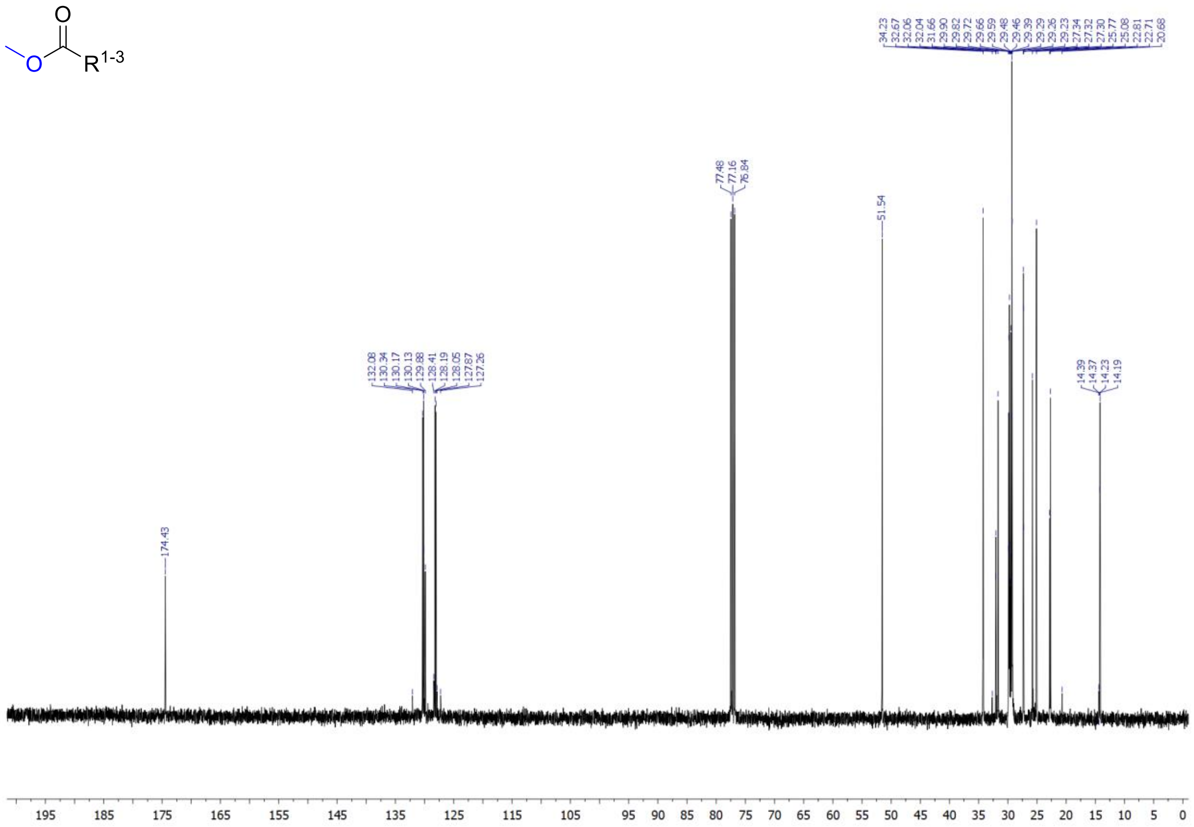

Figure S2. ^{13}C NMR spectrum (101 MHz, $CDCl_3$) of ester mixture **3a**

3.2 Fatty acid ethyl ester 3b

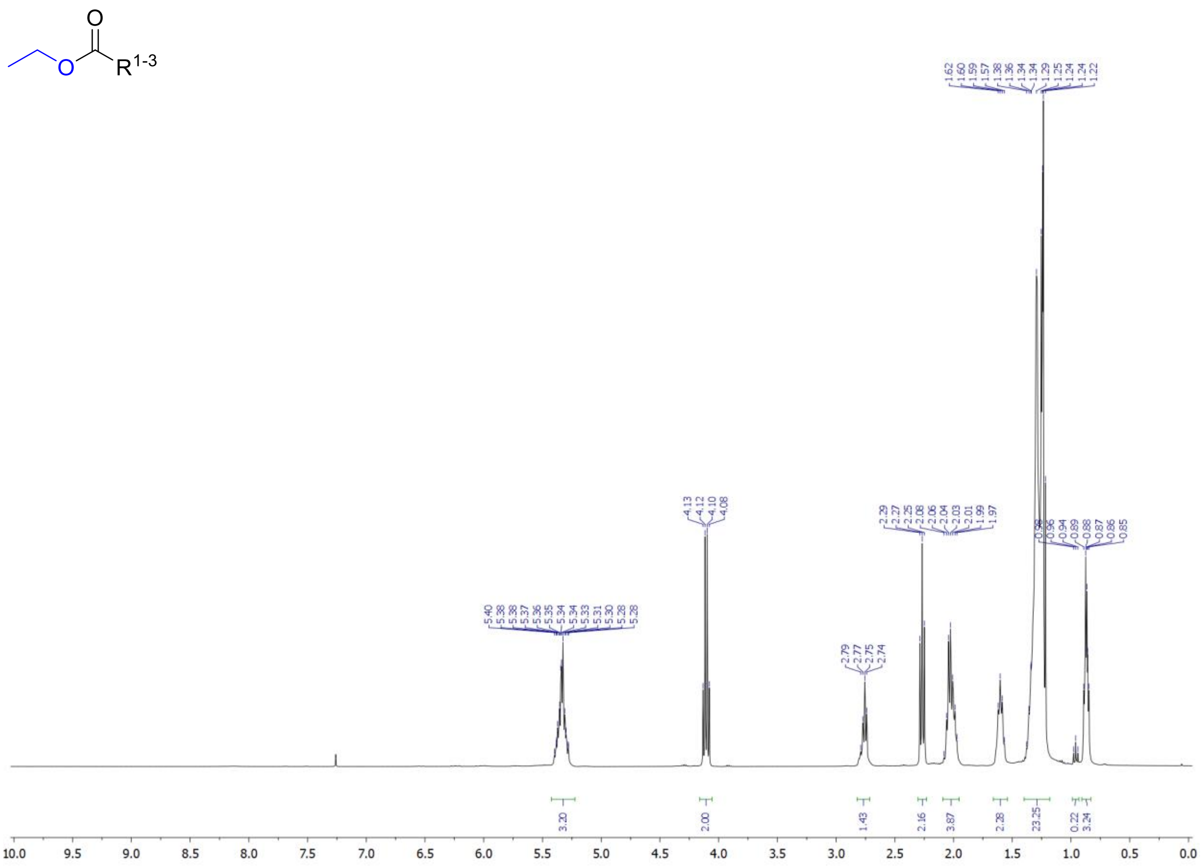

Figure S3. ^{1}H NMR spectrum (400 MHz, $CDCl_3$) of ester mixture **3b**

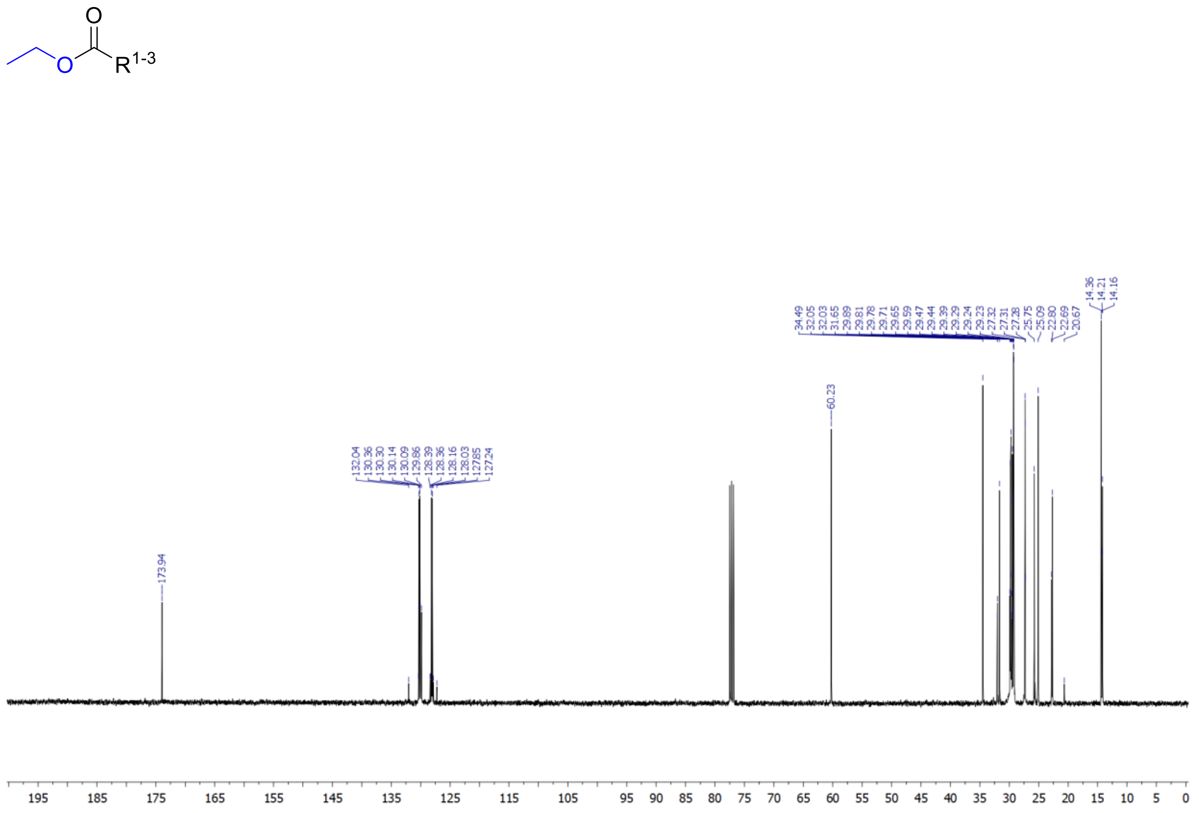

Figure S4. ^{13}C NMR spectrum (101 MHz, $CDCl_3$) of ester mixture **3b**

3.3 Fatty acid butyl ester 3c

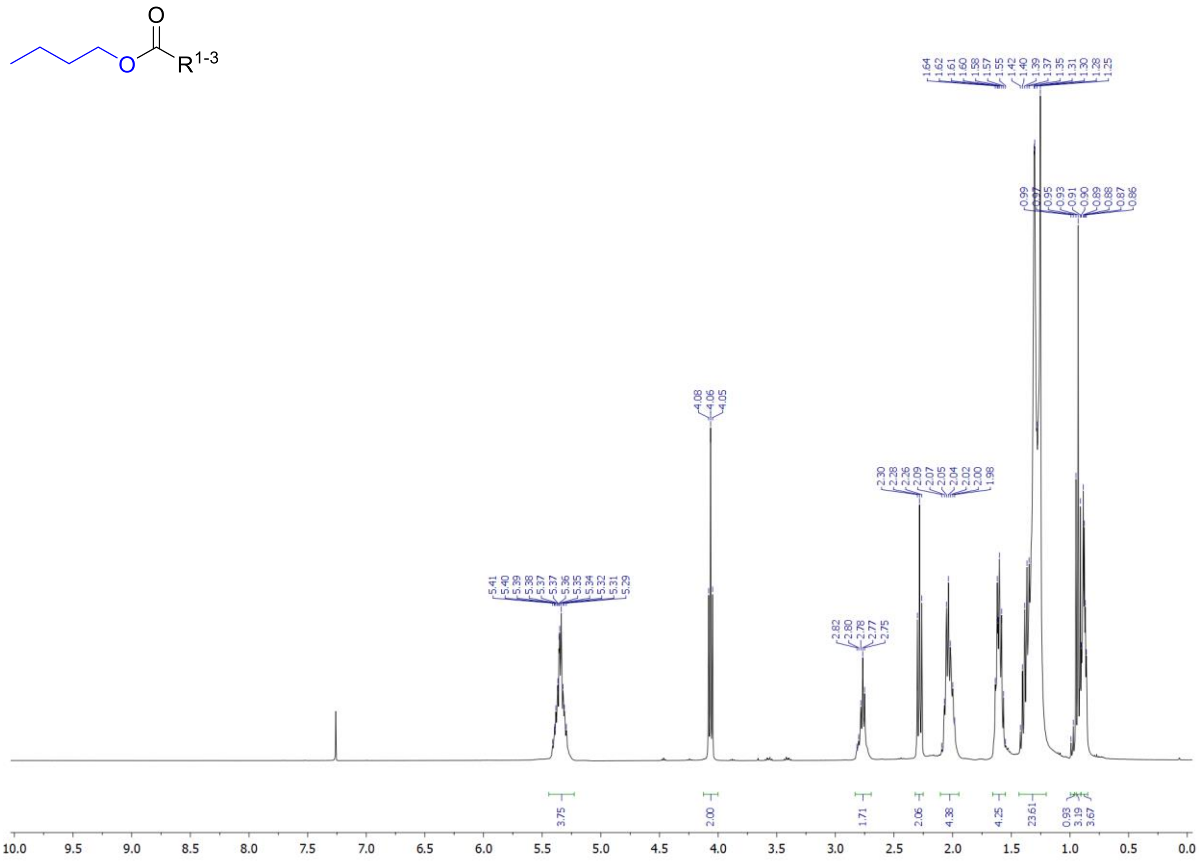

Figure S5. ^{1}H NMR spectrum (400 MHz, $CDCl_3$) of ester mixture **3c**

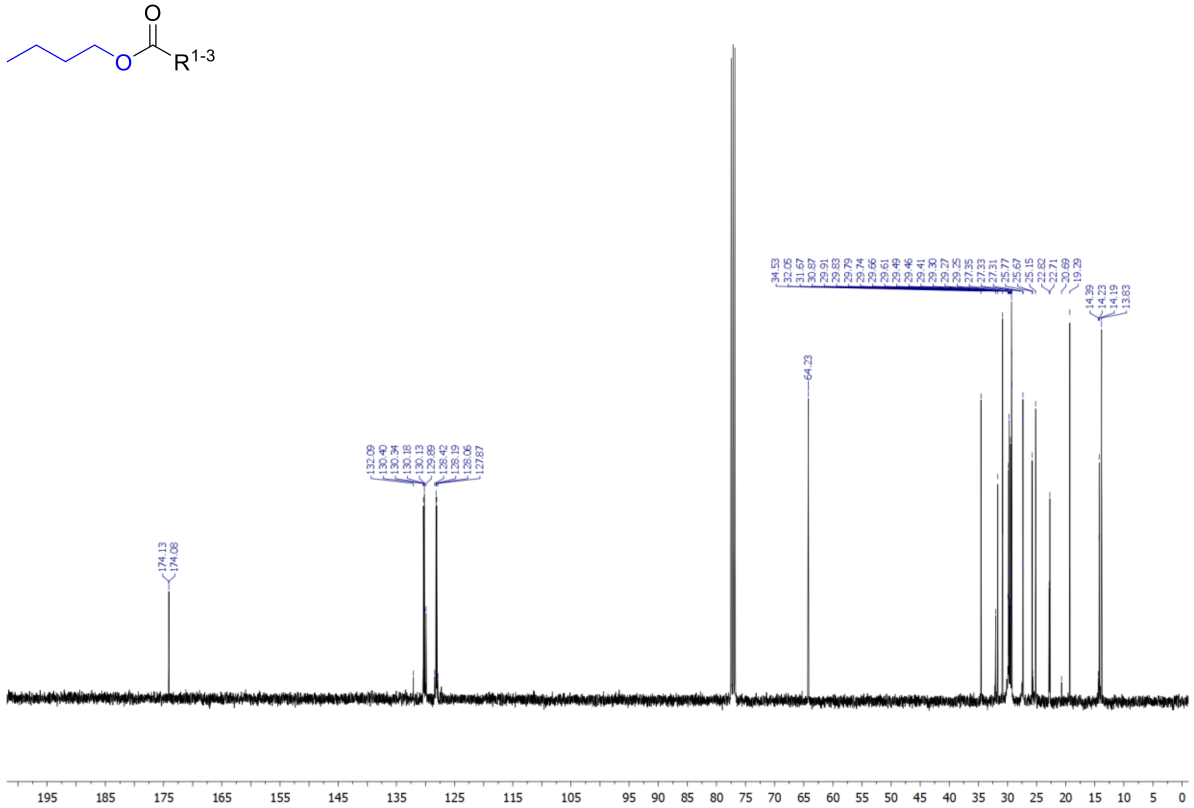

Figure S6. ^{13}C NMR spectrum (101 MHz, $CDCl_3$) of ester mixture **3c**

3.4 Fatty acid isobutyl ester 3d

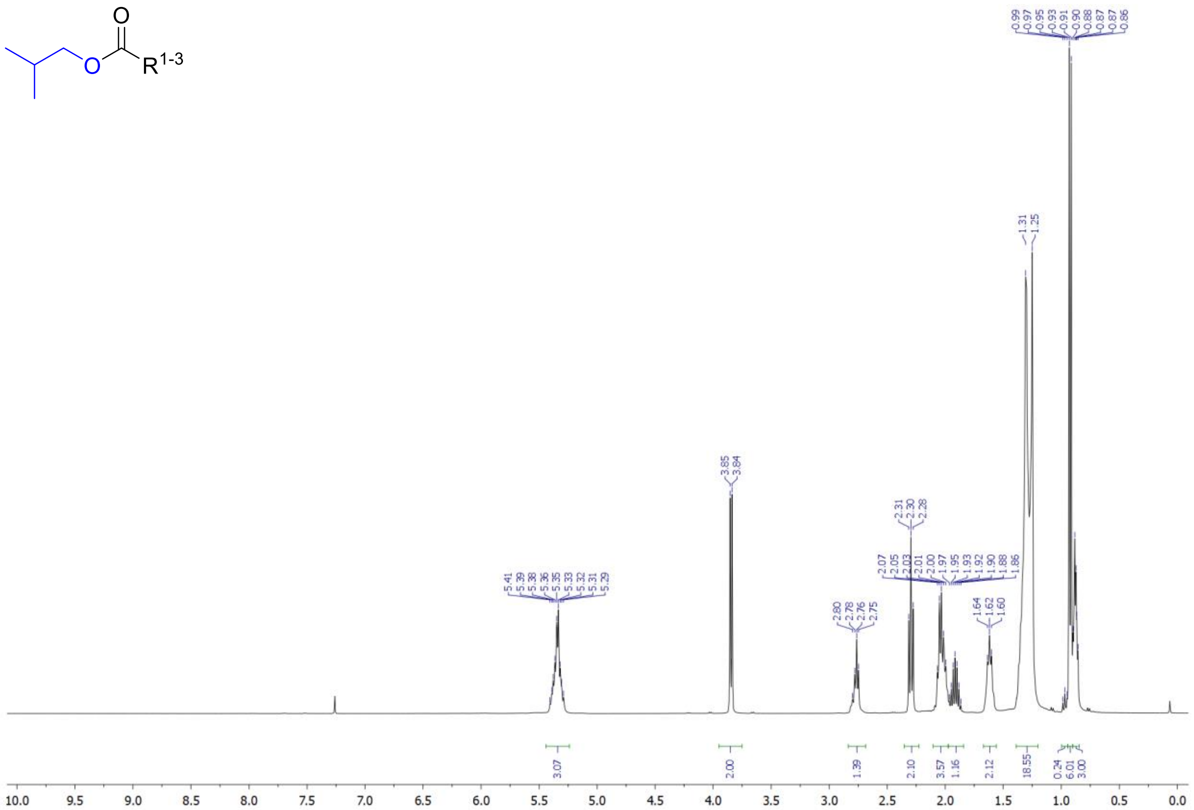

Figure S7. ^{1}H NMR spectrum (400 MHz, $CDCl_3$) of ester mixture **3d**

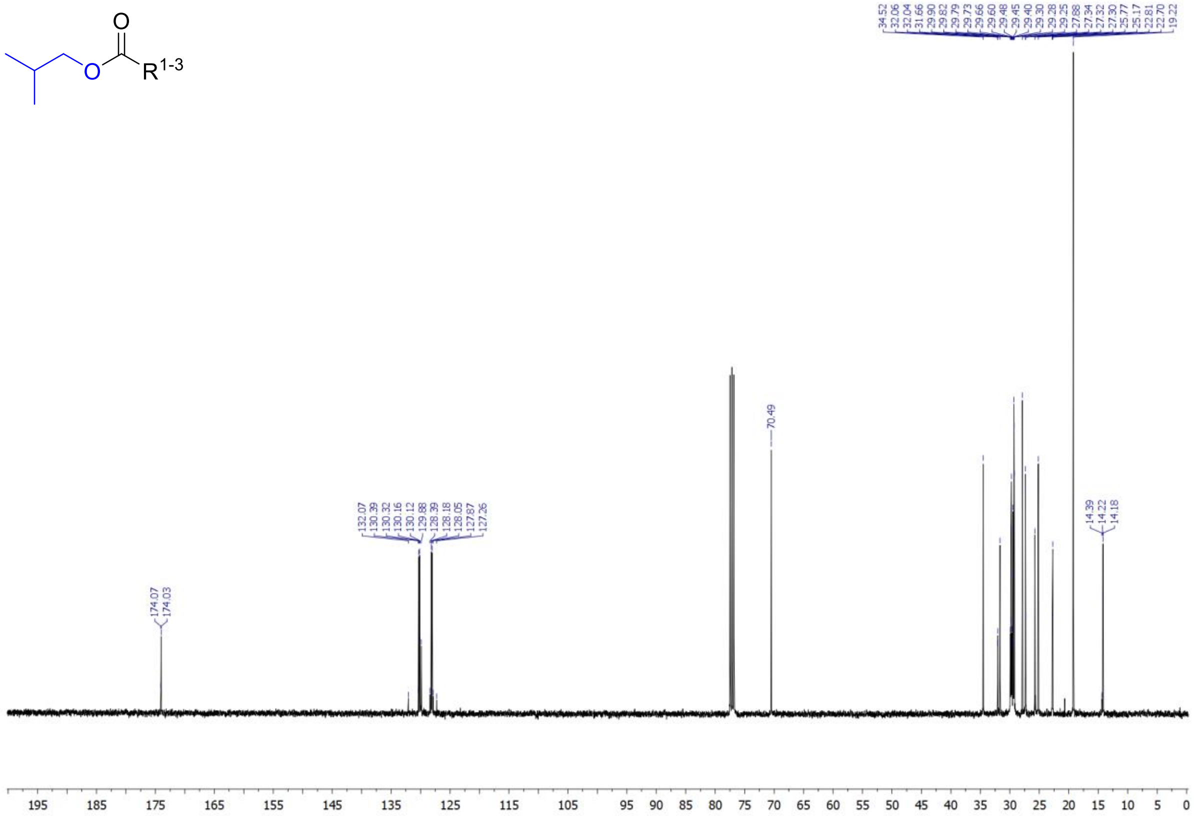

Figure S8. ^{13}C NMR spectrum (101 MHz, $CDCl_3$) of ester mixture **3d**

3.5 Fatty acid 2-propynyl ester 3e

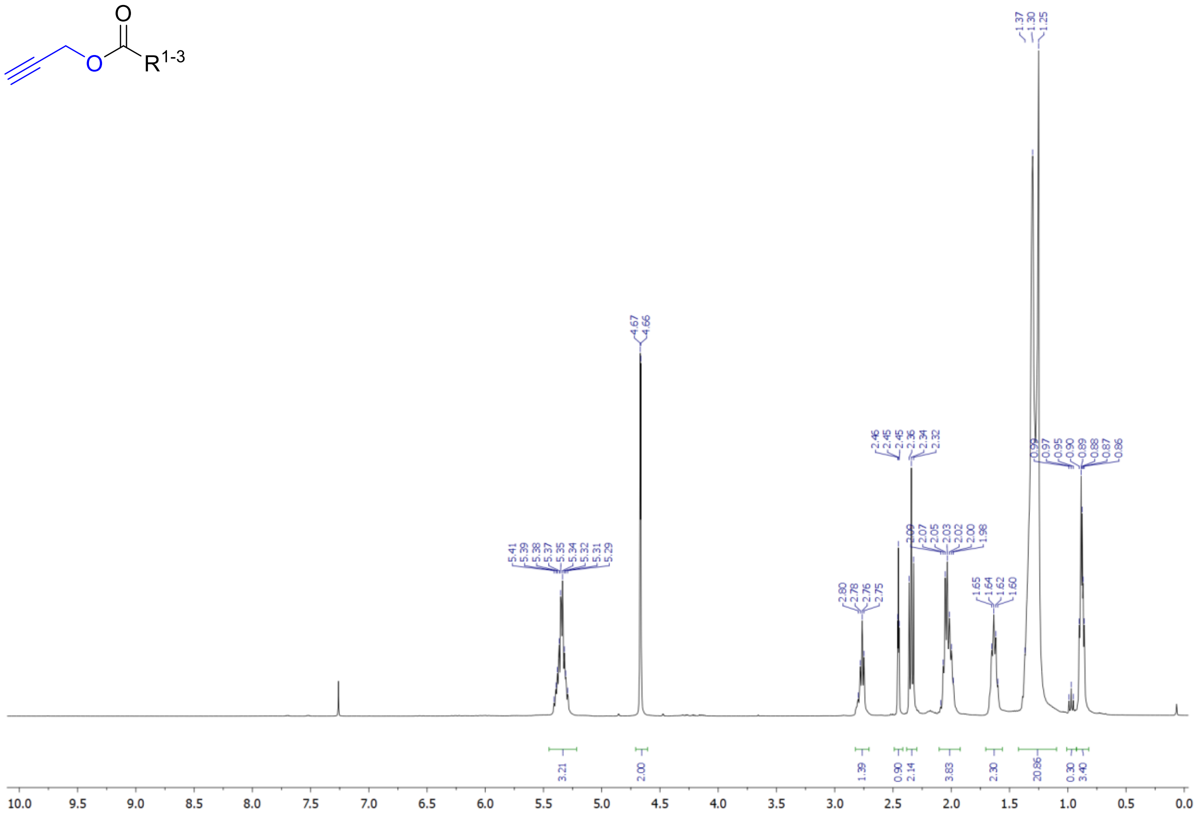

Figure S9. ^{1}H NMR spectrum (400 MHz, $CDCl_3$) of ester mixture **3e**

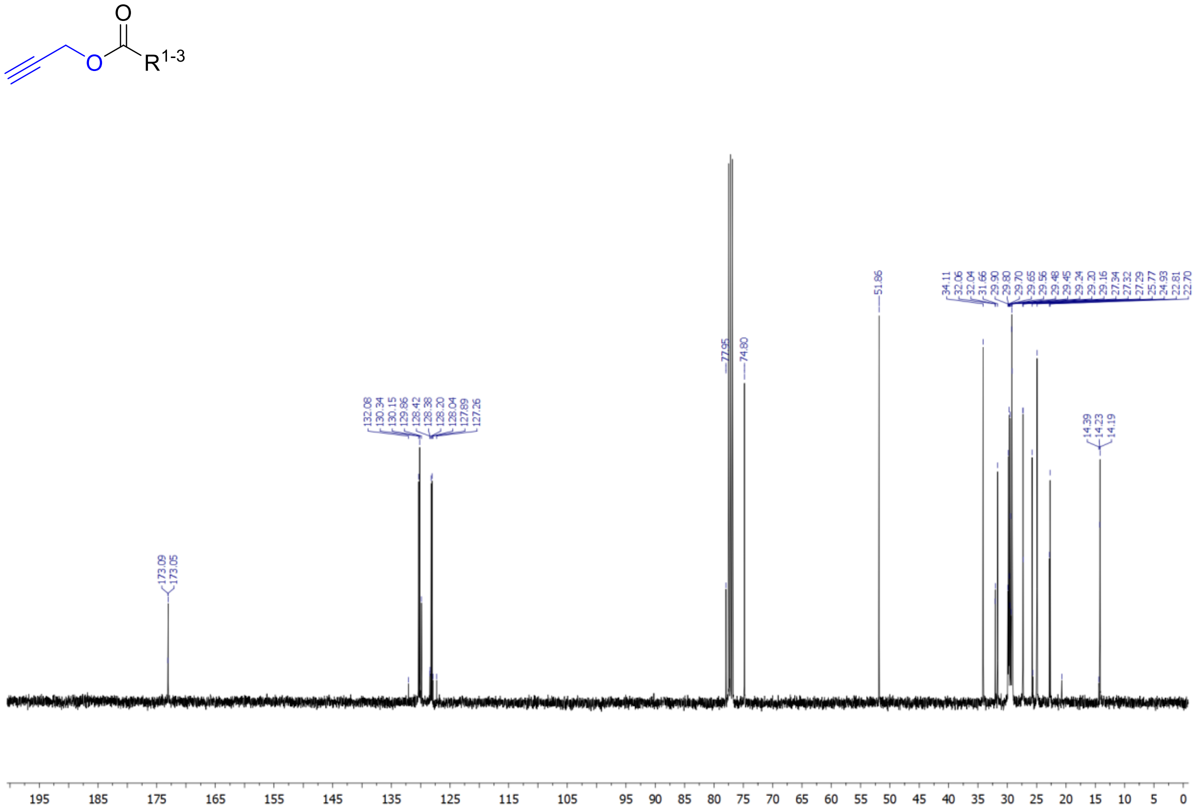

Figure S10. ^{13}C NMR spectrum (101 MHz, $CDCl_3$) of ester mixture **3e**

3.6 Fatty acid tetrahydrofurfuryl ester 3f

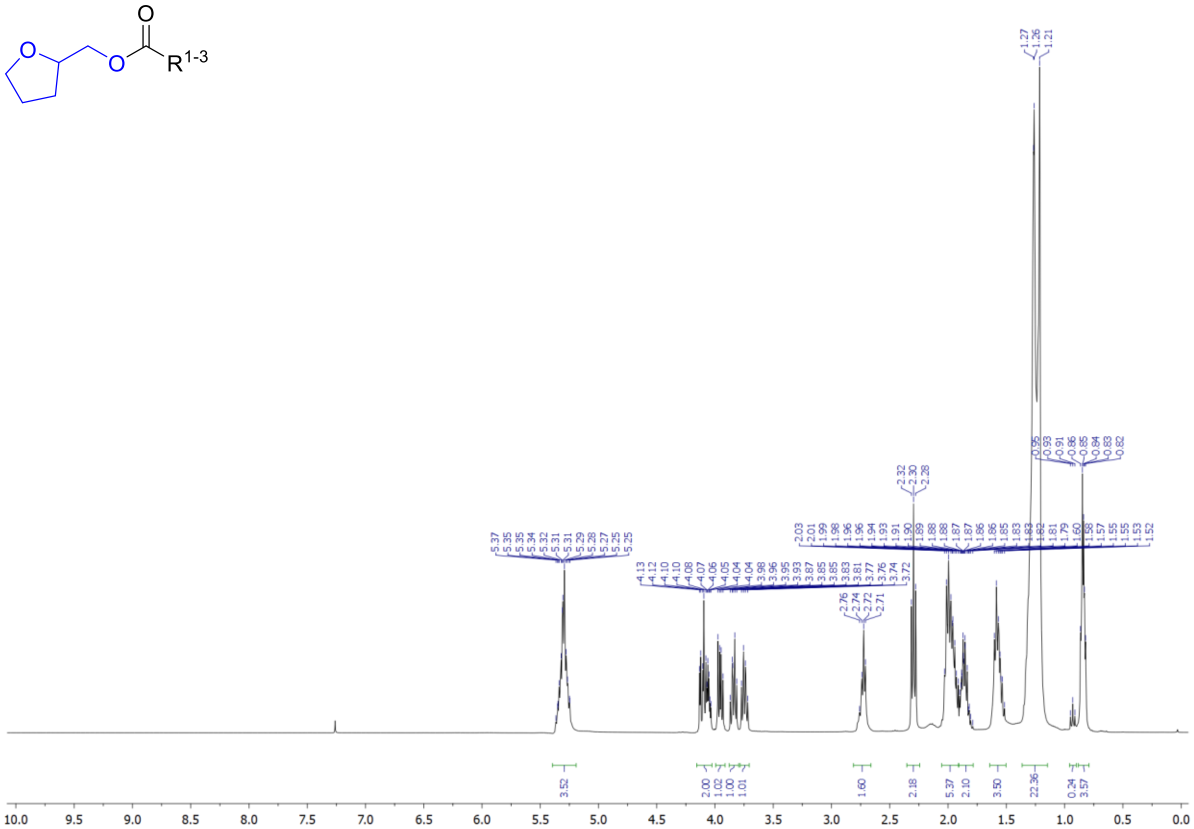

Figure S11. ^{1}H NMR spectrum (400 MHz, $CDCl_3$) of ester mixture **3f**

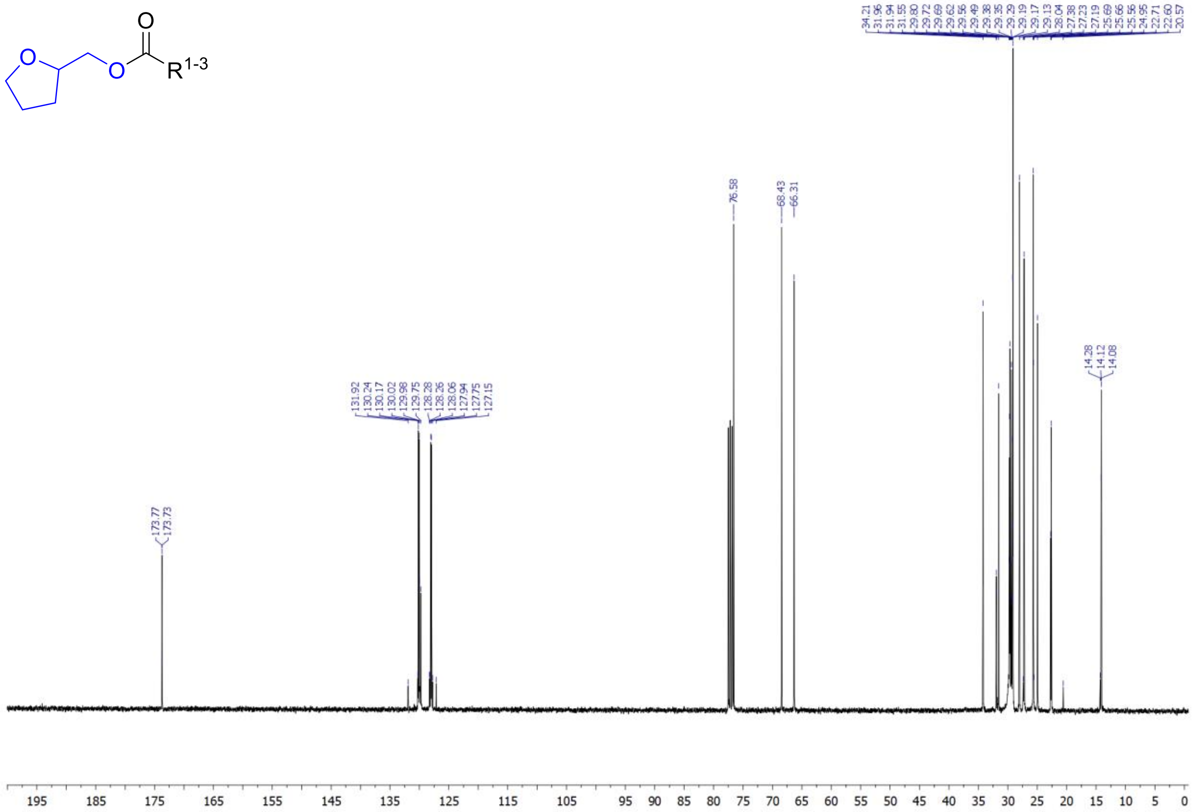

Figure S12. ^{13}C NMR spectrum (101 MHz, $CDCl_3$) of ester mixture **3f**

3.7 Fatty acid 3-hexenyl ester 3g

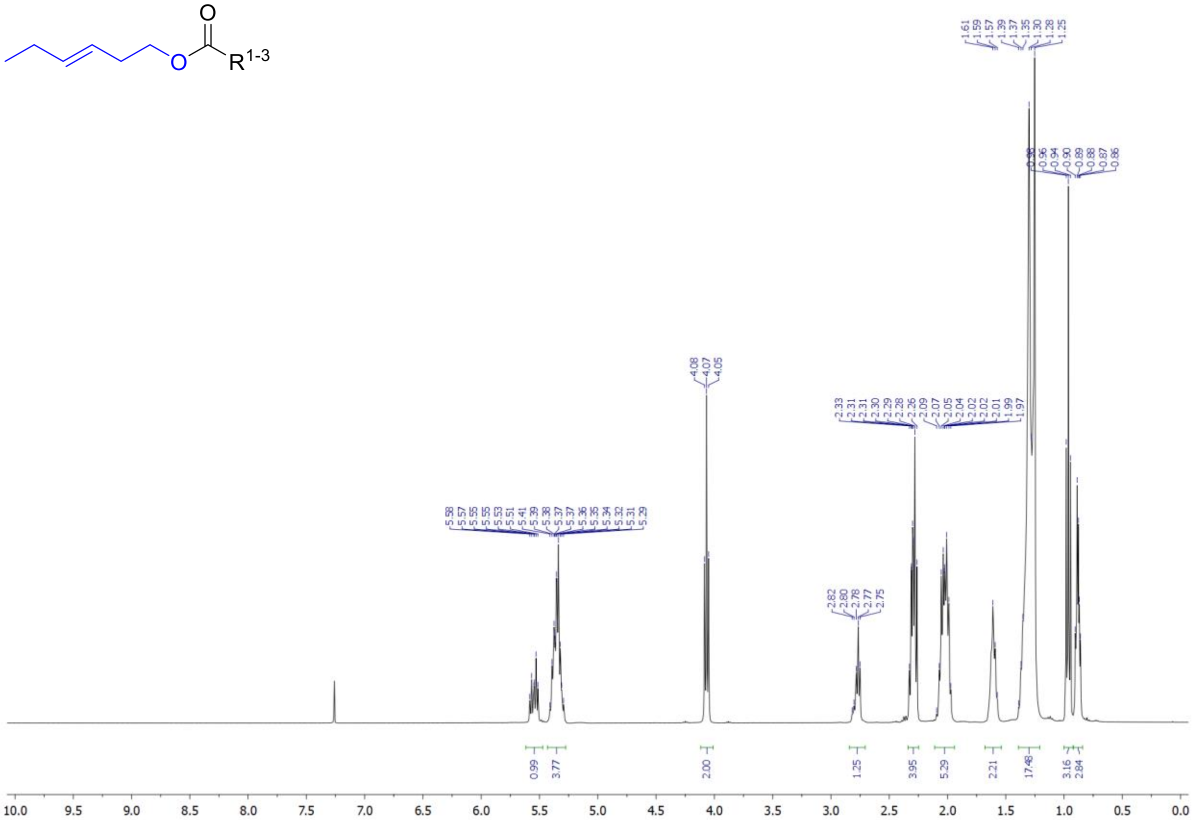

Figure S13. ^{1}H NMR spectrum (400 MHz, $CDCl_3$) of ester mixture **3g**

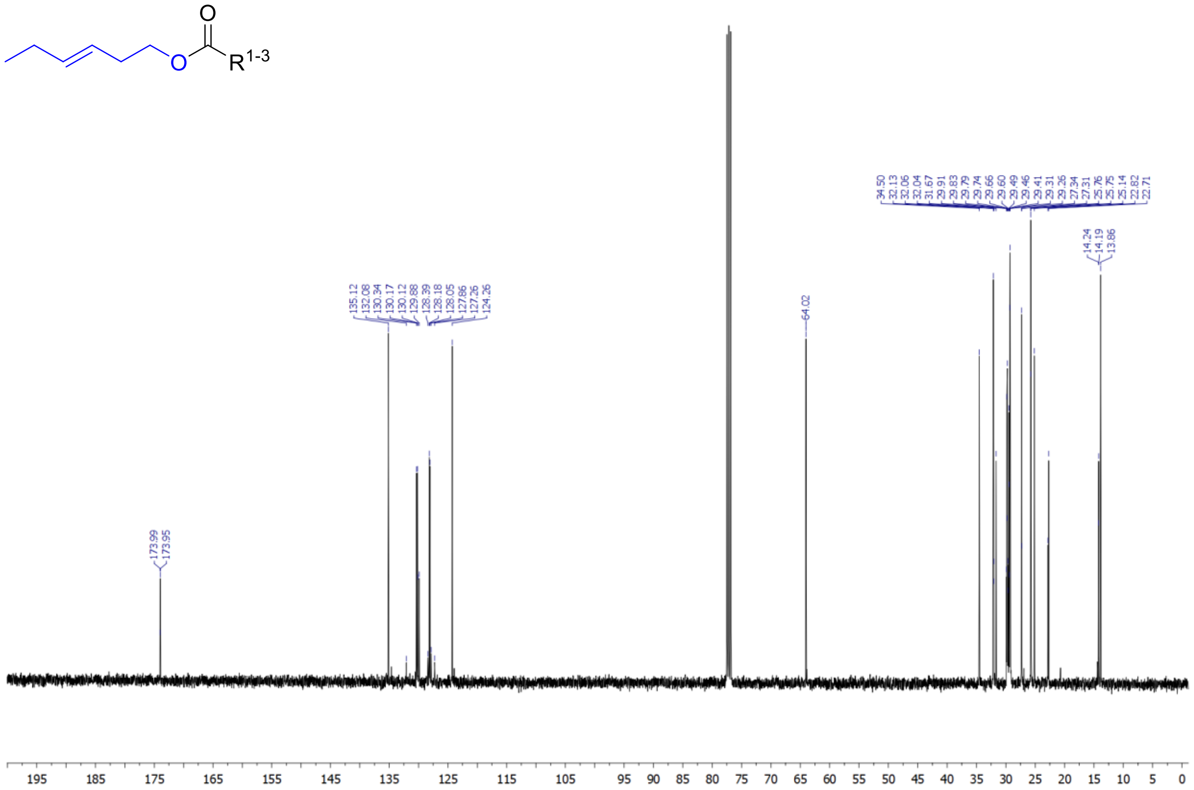

Figure S14. ^{13}C NMR spectrum (101 MHz, $CDCl_3$) of ester mixture **3g**

3.8 Fatty acid 3-butenyl ester 3h

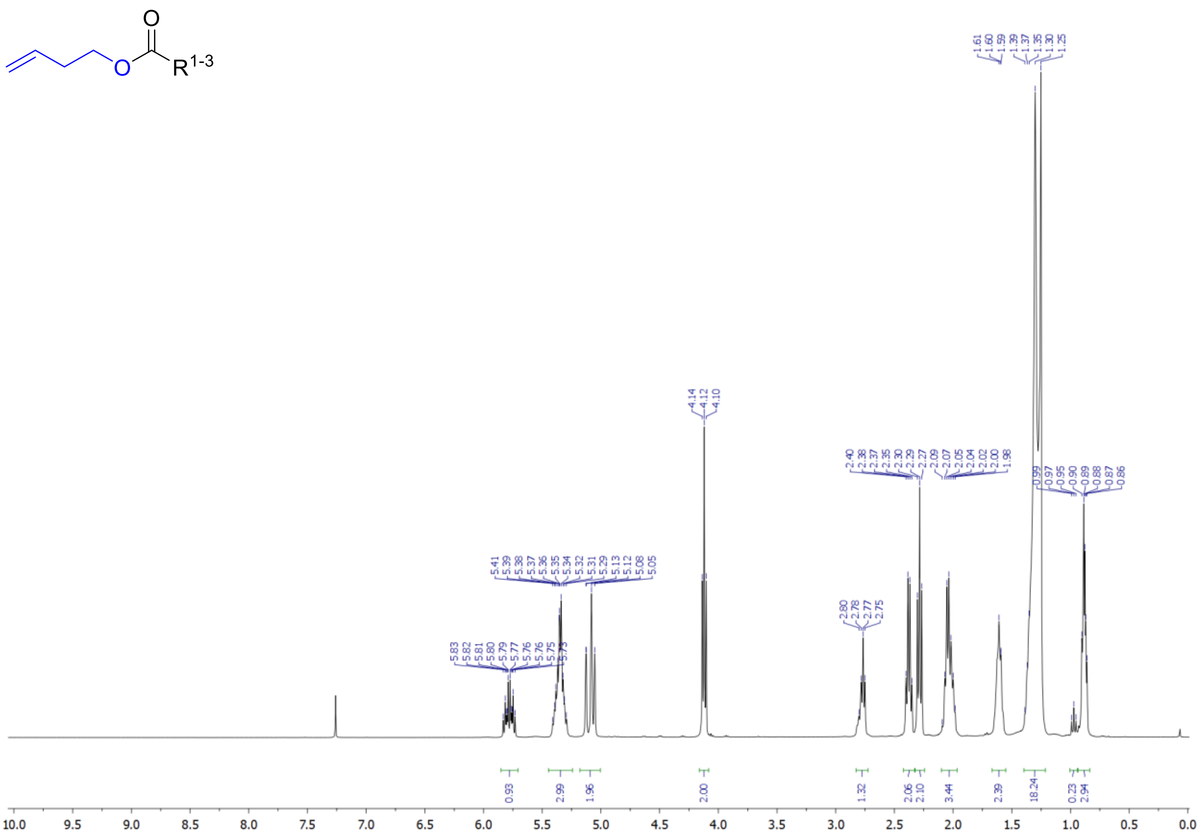

Figure S15. ^{1}H NMR spectrum (400 MHz, $CDCl_3$) of ester mixture **3h**

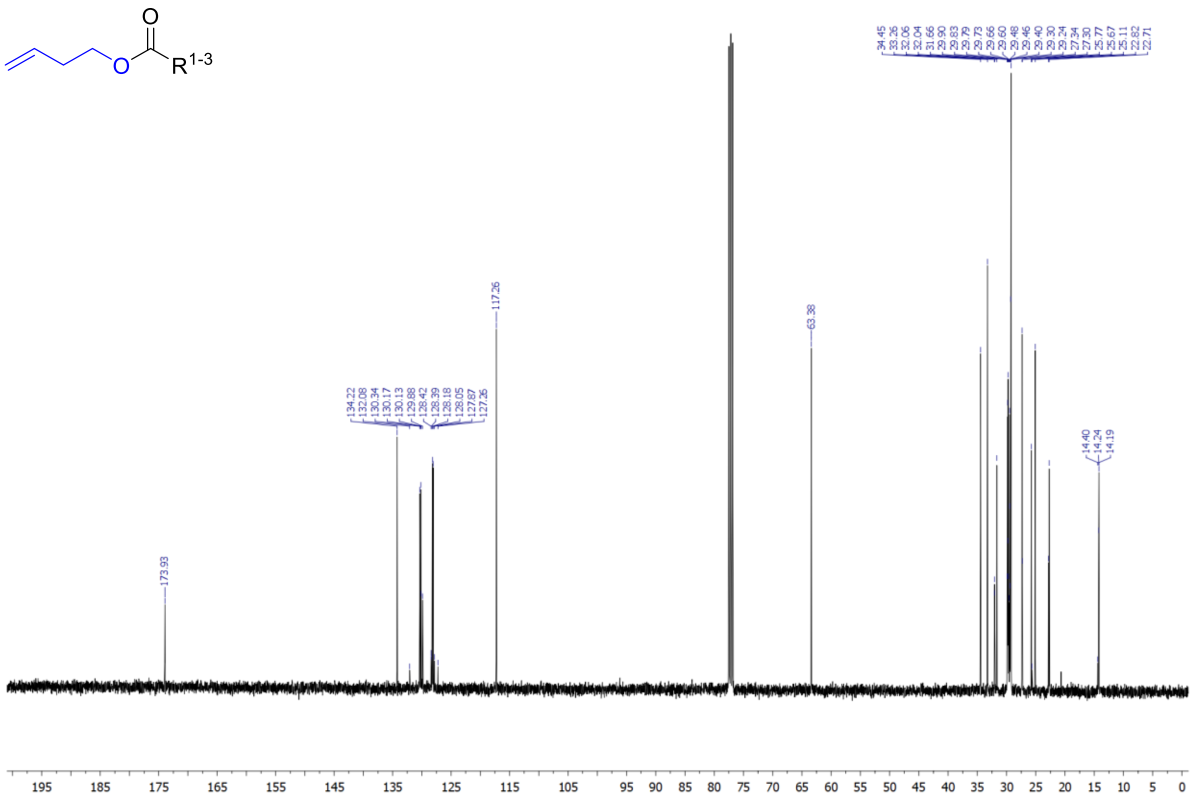

Figure S16. ^{13}C NMR spectrum (101 MHz, $CDCl_3$) of ester mixture **3h**

3.9 δ-Valerolactone (4e)

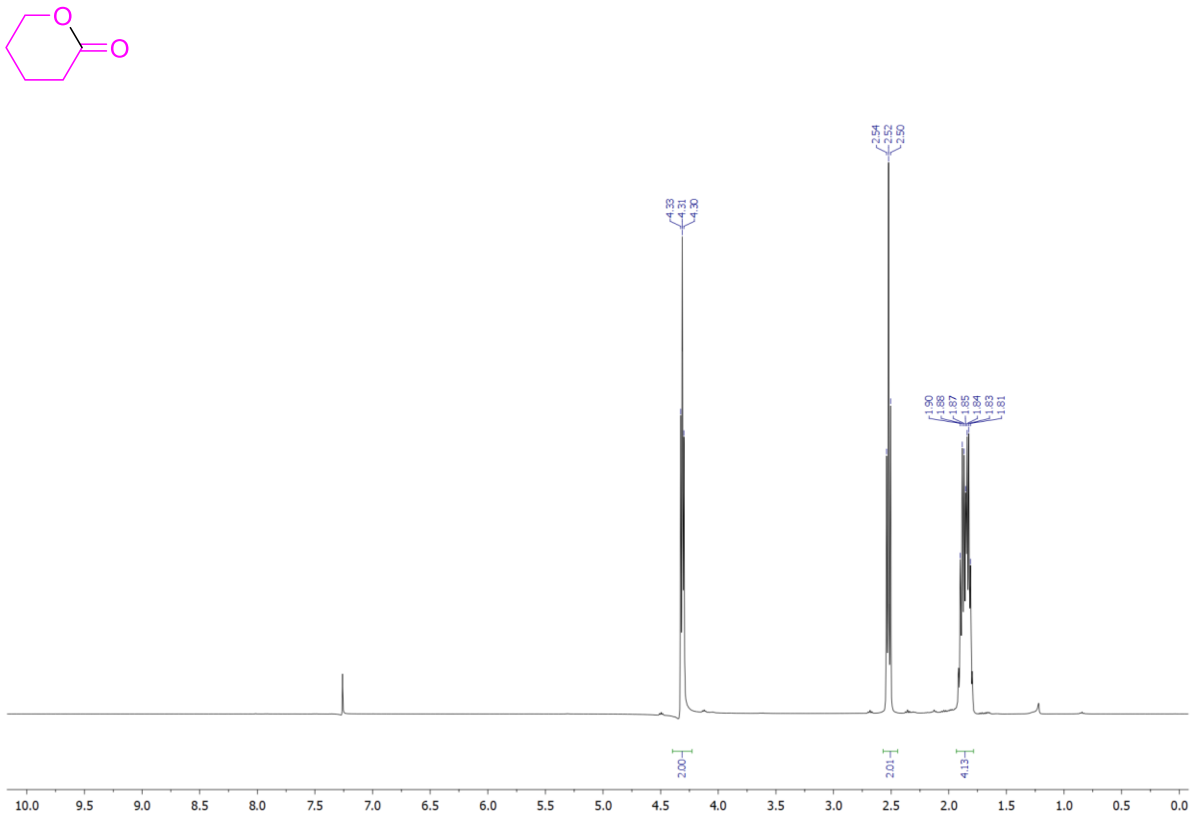

Figure S17. ^{1}H NMR spectrum (400 MHz, $CDCl_3$) of compound **4e**

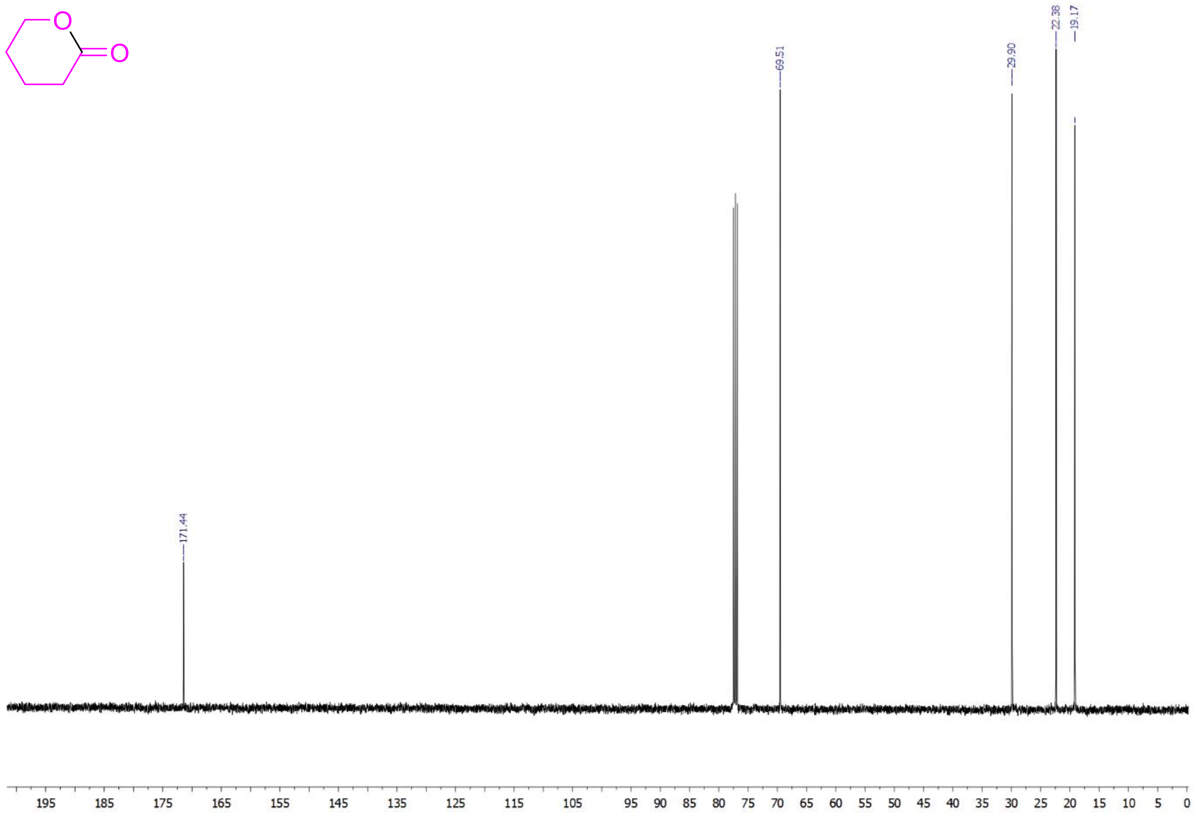

Figure S18. ^{13}C NMR spectrum (101 MHz, $CDCl_3$) of compound **4e**

3.10 Propane-1,2,3-triyl tripentanoate (4f)

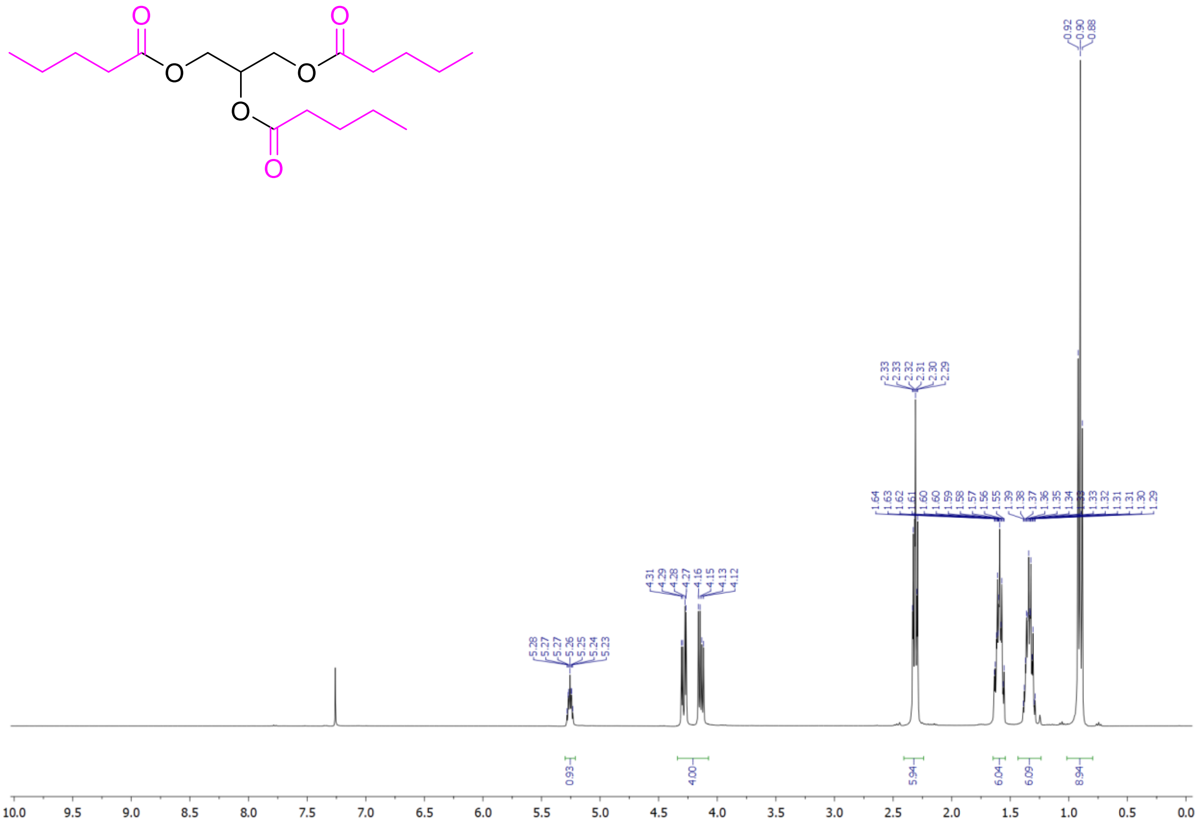

Figure S19. ^{1}H NMR spectrum (400 MHz, $CDCl_3$) of compound **4f**

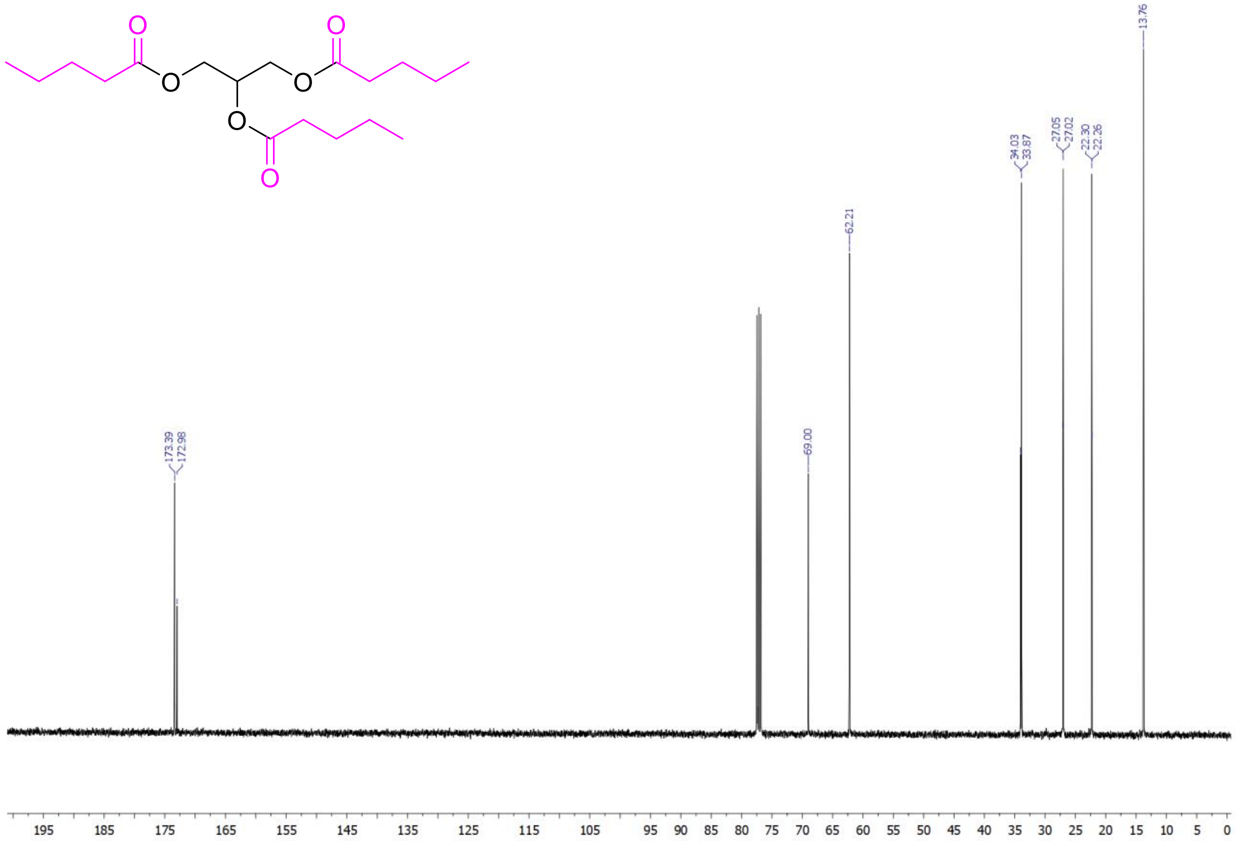

Figure S20. ^{1}H NMR spectrum (400 MHz, $CDCl_3$) of compound **4f**

3.11 Propane-1,2,3-triyl trinonanoate (4g)

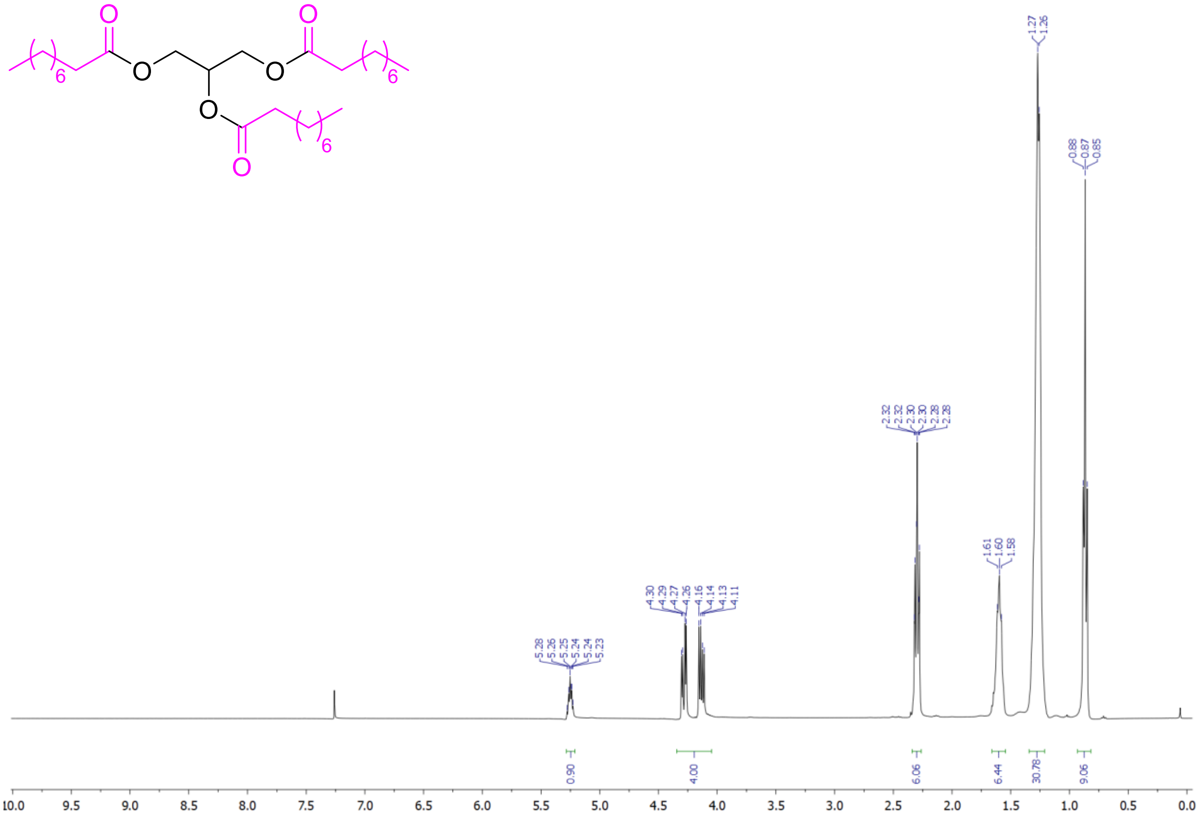

Figure S21. ^{1}H NMR spectrum (400 MHz, $CDCl_3$) of compound **4g**

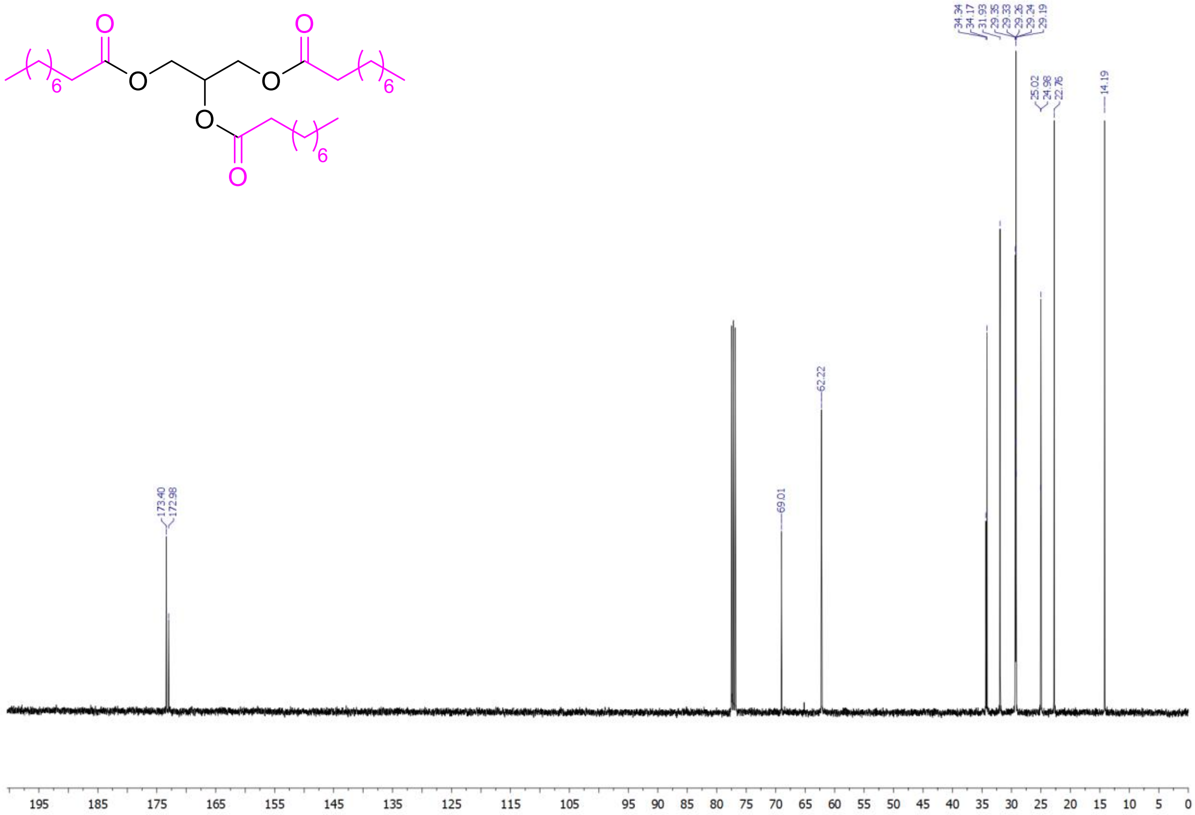

Figure S22. ^{13}C NMR spectrum (101 MHz, $CDCl_3$) of compound **4g**

3.12 Propane-1,2,3-triyl tris(decanoate) (4h)

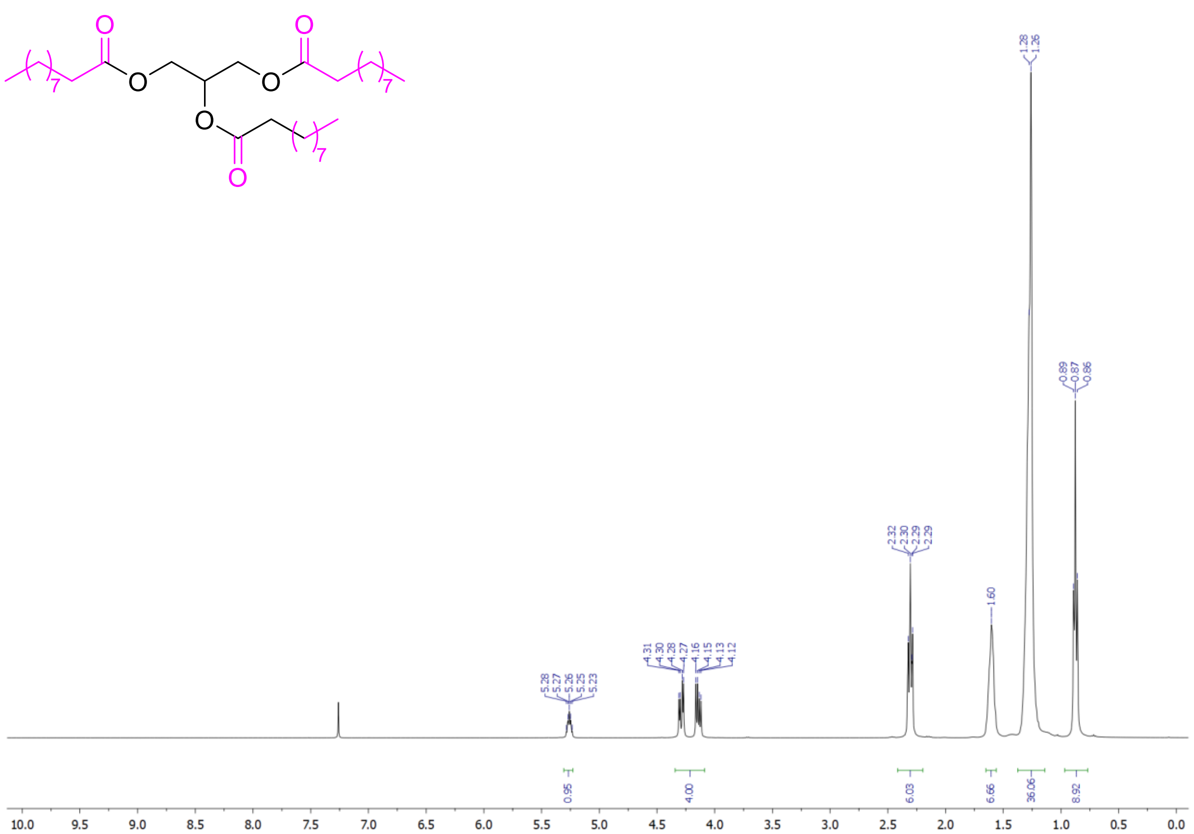

Figure S23. ^{1}H NMR spectrum (400 MHz, $CDCl_3$) of compound **4h**

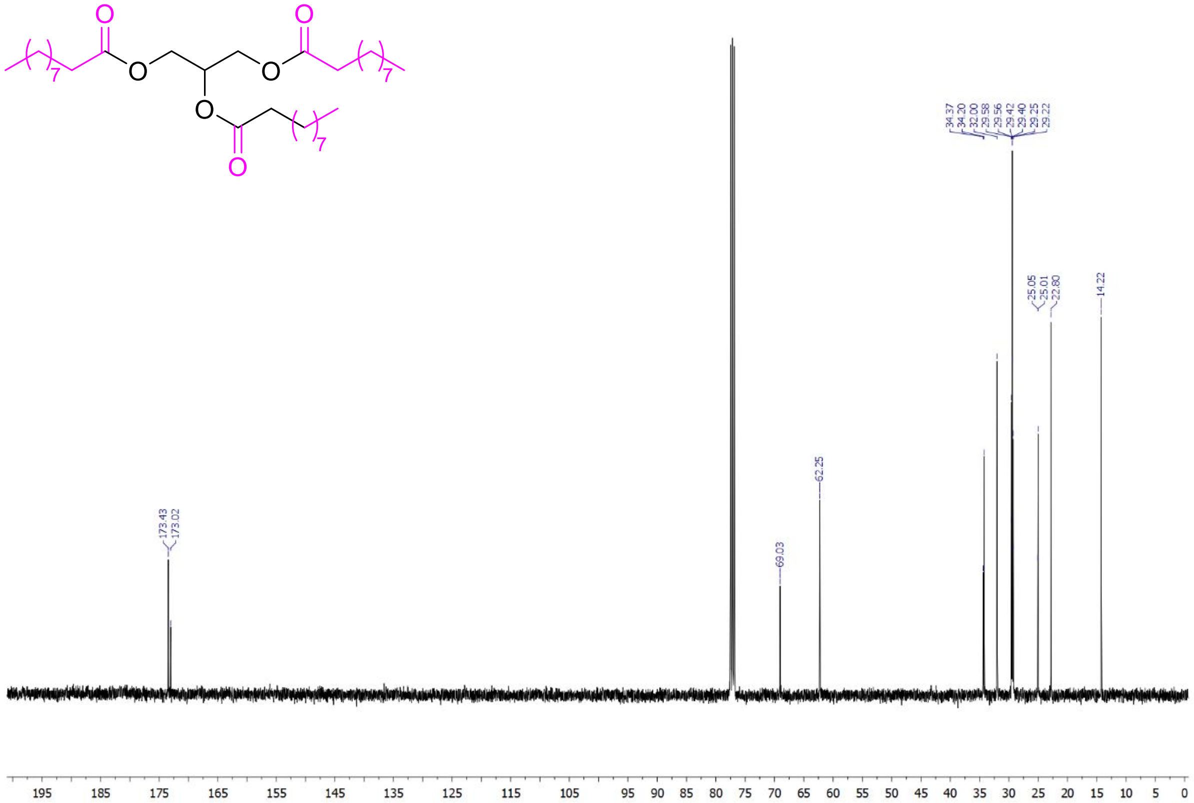

Figure S24. ^{13}C NMR spectrum (101 MHz, $CDCl_3$) of compound **4h**

3.13 Propane-1,2,3-triyl tridodecanoate (4i)

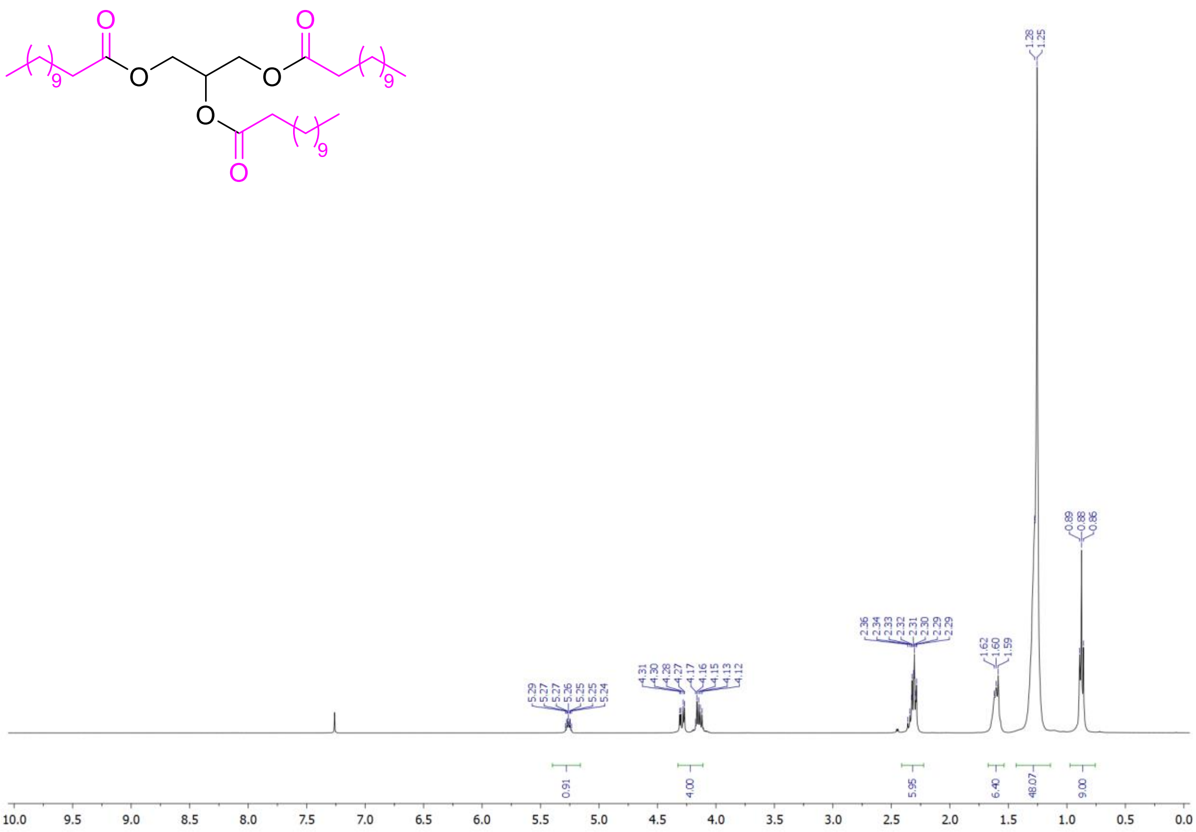

Figure S25. ^{1}H NMR spectrum (400 MHz, $CDCl_3$) of compound **4i**

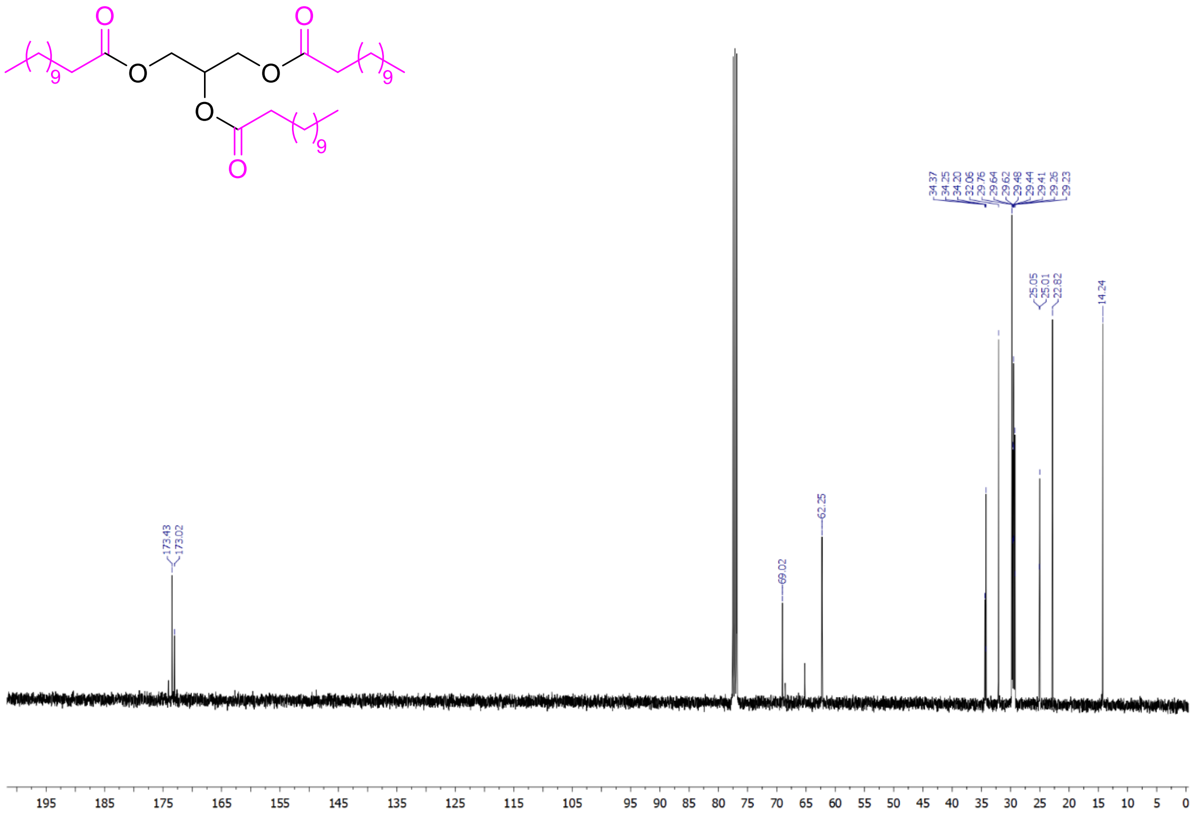

Figure S26. ^{13}C NMR spectrum (101 MHz, $CDCl_3$) of compound **4i**

3.14 Methyl benzoate (5a)

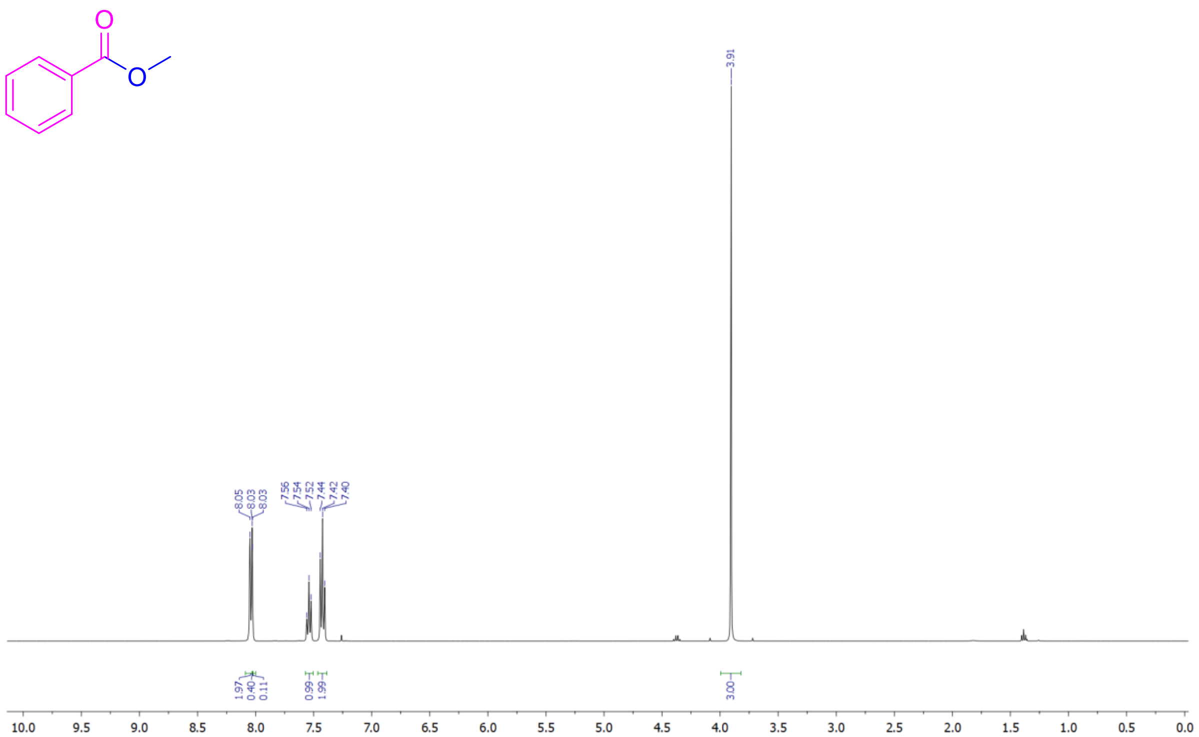

Figure S27. ^{1}H NMR spectrum (400 MHz, $CDCl_3$) of compound **5a**

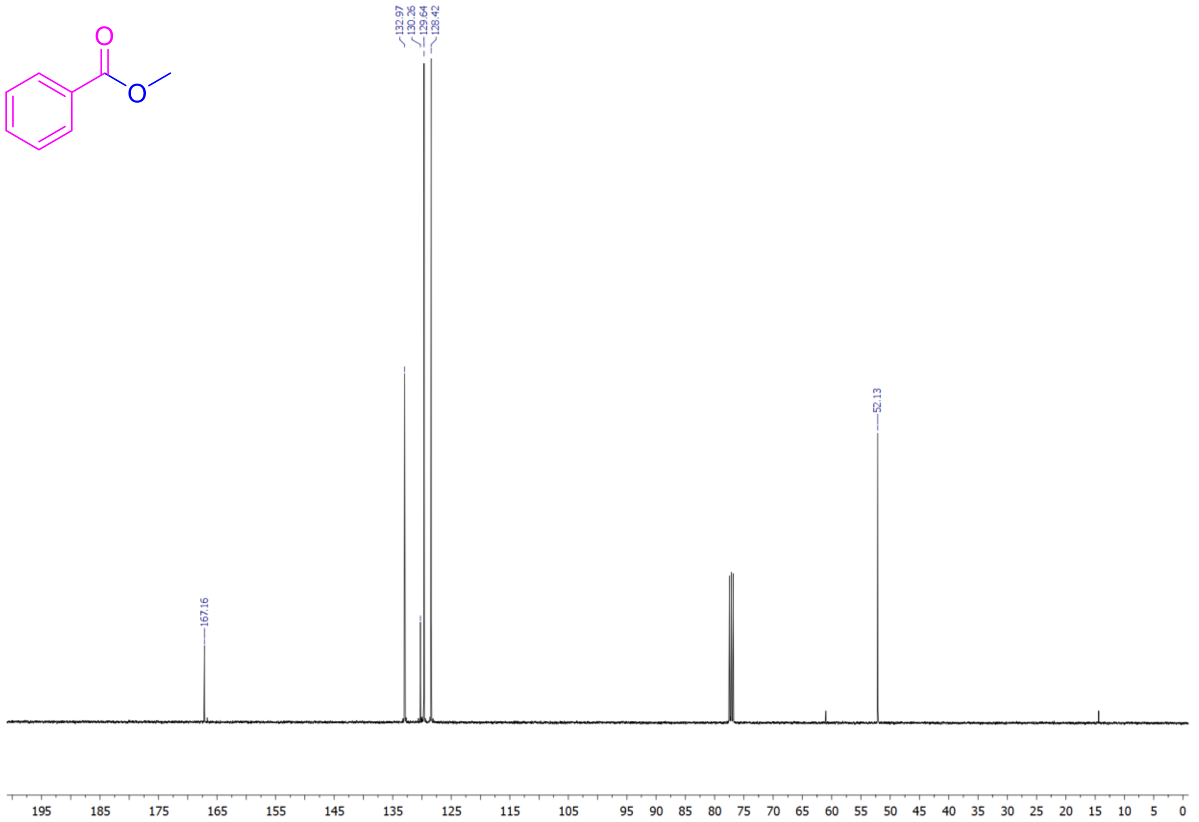

Figure S28. ^{13}C NMR spectrum (101 MHz, $CDCl_3$) of compound **5a**

3.15 Methyl cinnamate (5b)

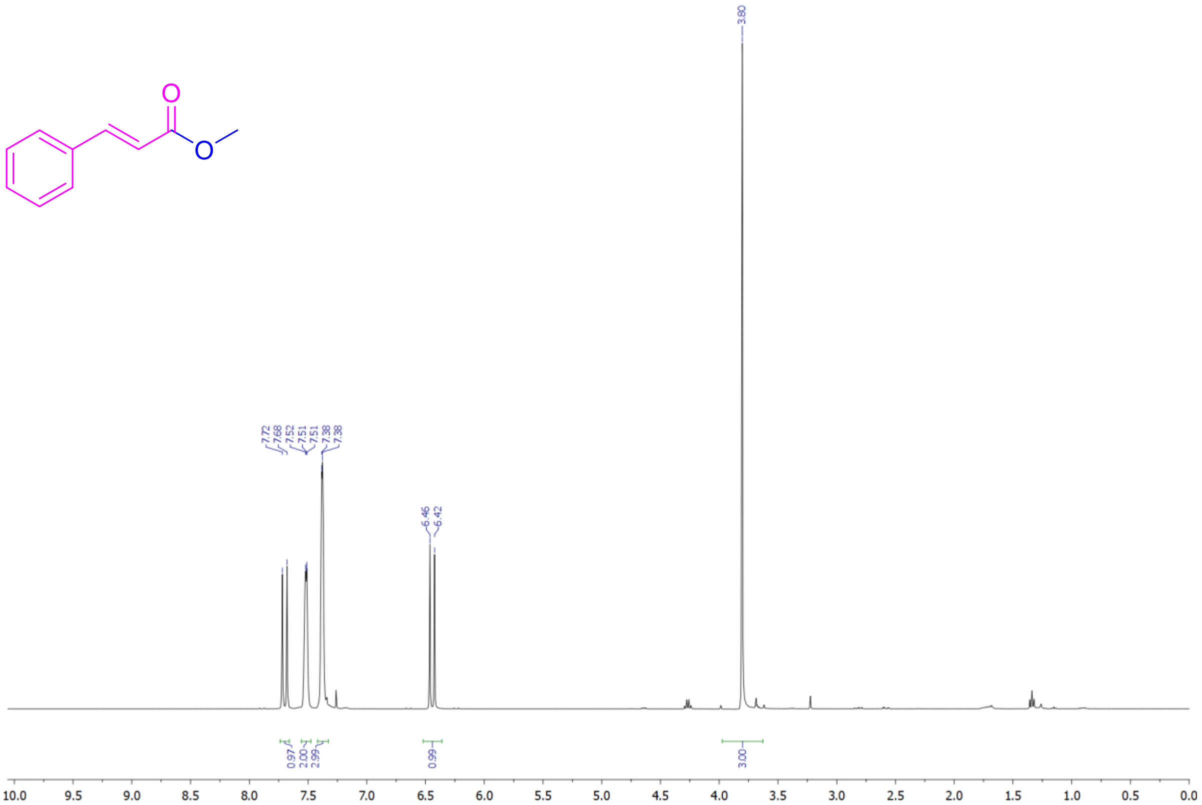

Figure S29. ^{1}H NMR spectrum (400 MHz, $CDCl_3$) of compound **5b**

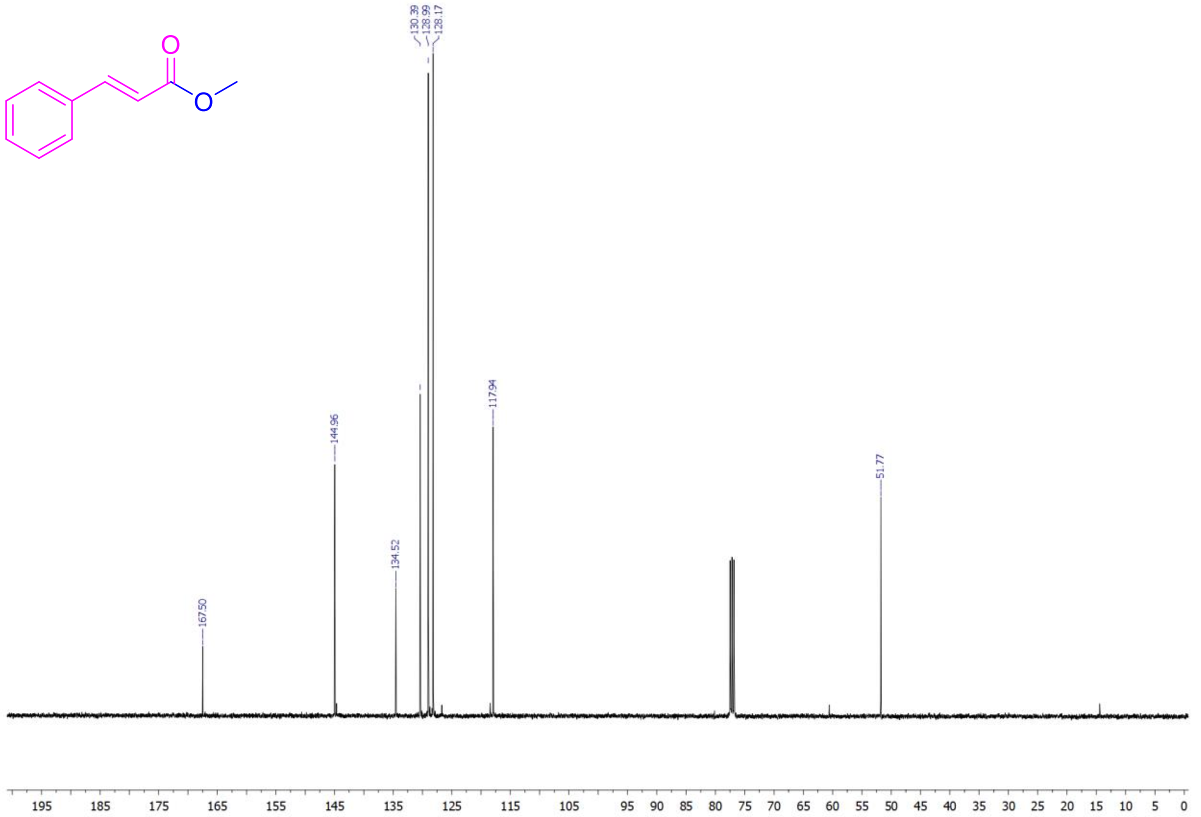

Figure S30. ^{13}C NMR spectrum (101 MHz, $CDCl_3$) of compound **5b**

3.16 Methyl 3-phenylpropiolate (5c)

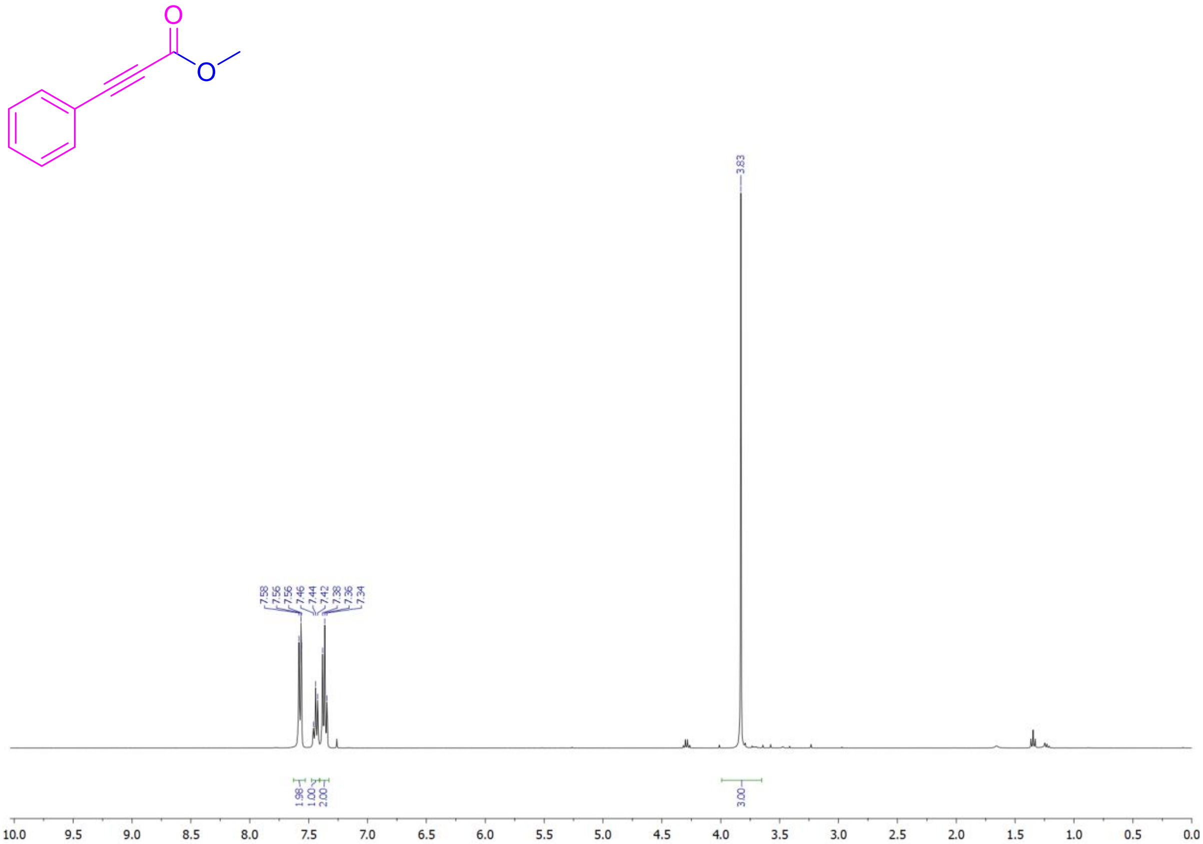

Figure S31. ^{1}H NMR spectrum (400 MHz, $CDCl_3$) of compound **5c**

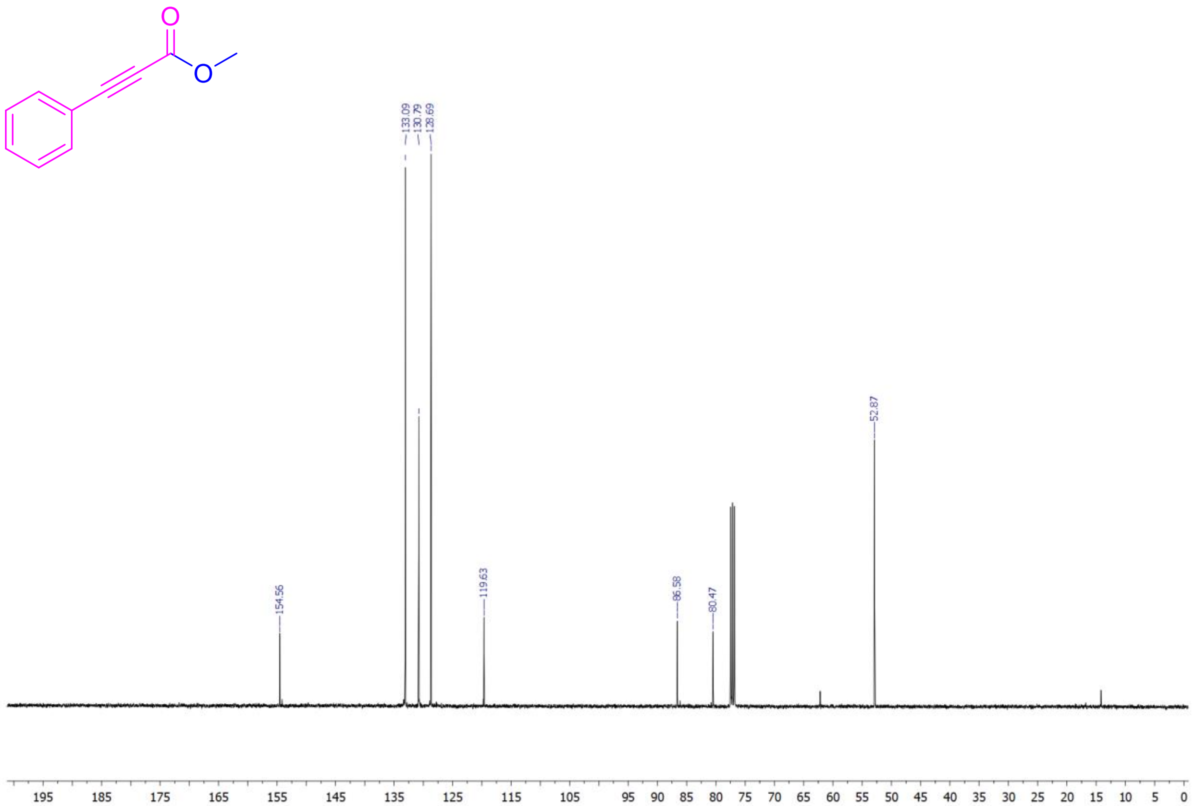

Figure S32. ^{13}C NMR spectrum (101 MHz, $CDCl_3$) of compound **5c**

3.17 Methyl 2-bromopropanoate (5d)

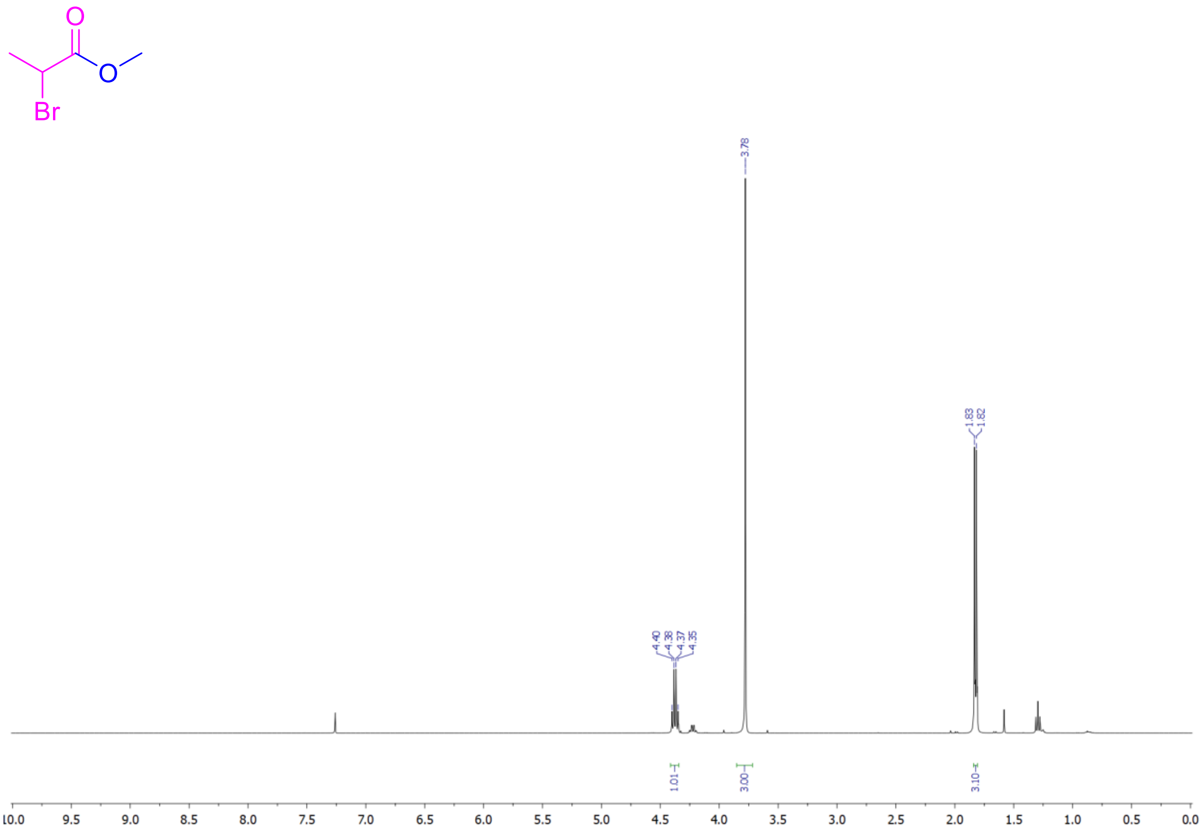

Figure S33. ^{1}H NMR spectrum (400 MHz, $CDCl_3$) of compound **5d**

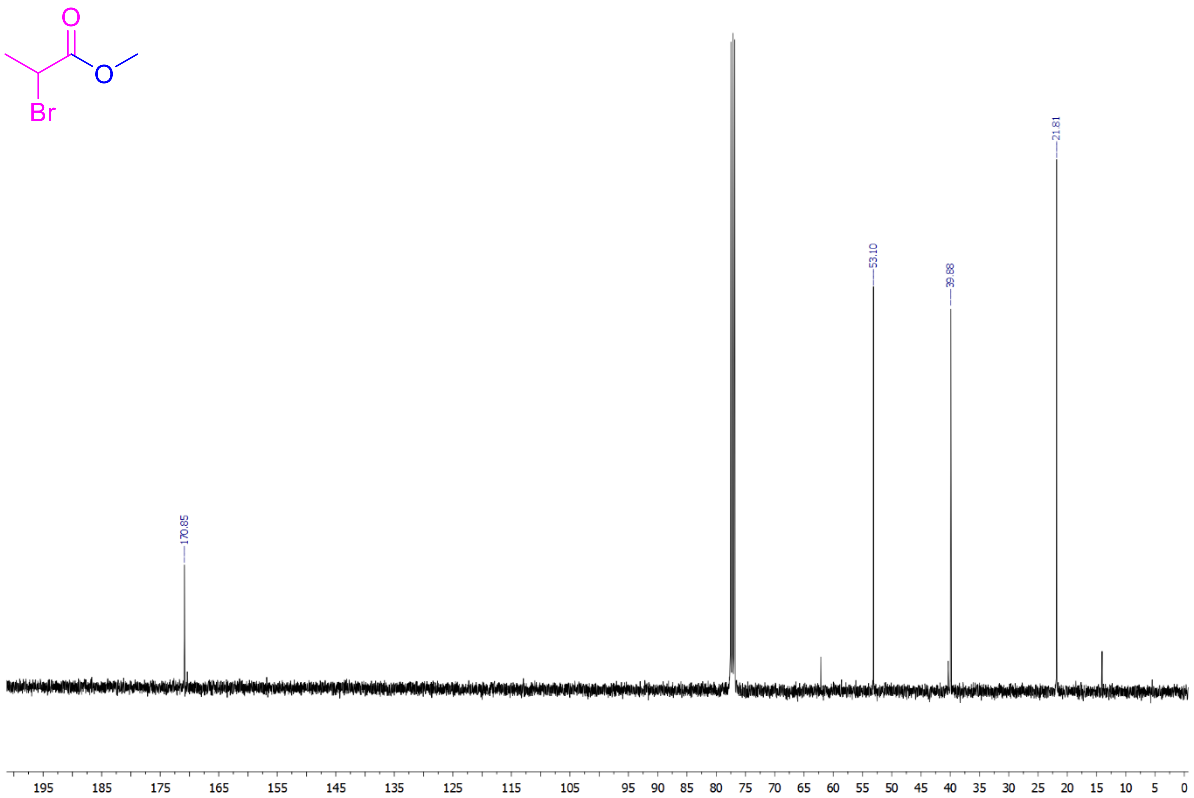

Figure S34. ^{13}C NMR spectrum (101 MHz, $CDCl_3$) of compound **5d**

3.18 Methyl 5-hydroxypentanoate (5e)

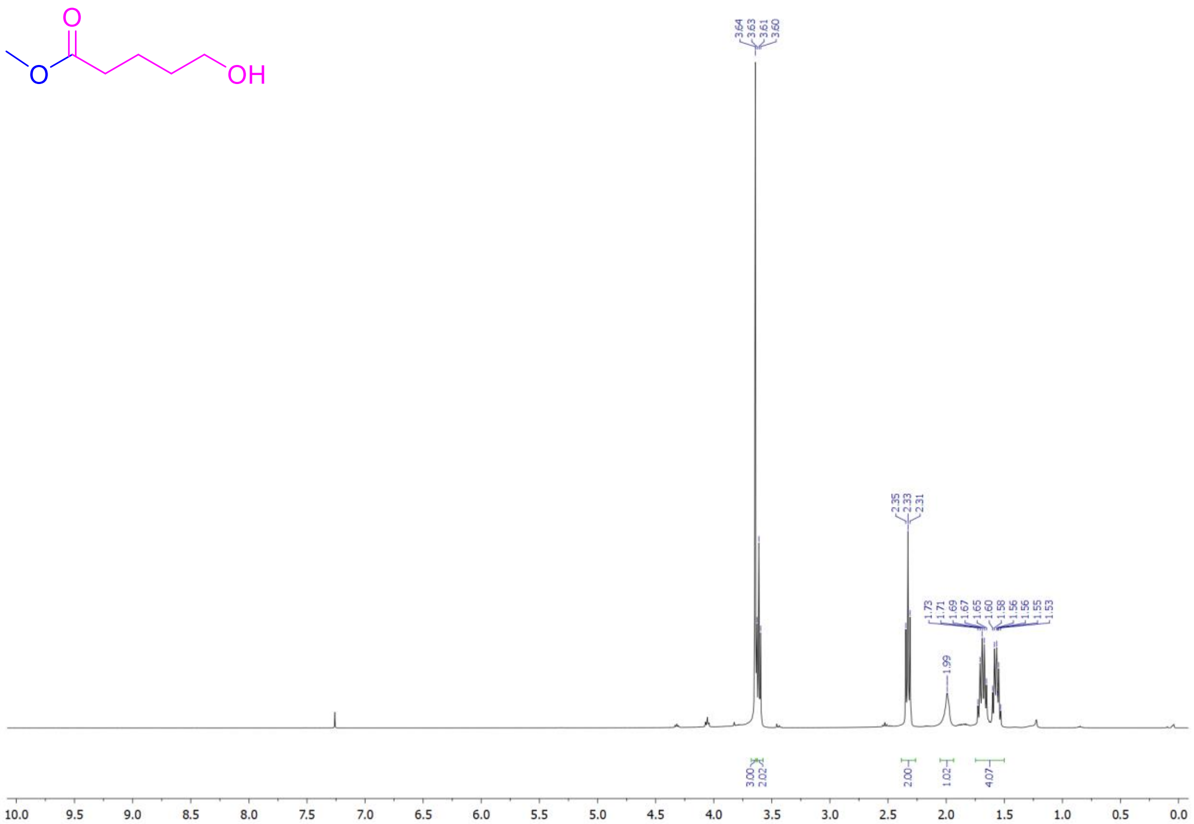

Figure S35. ^{1}H NMR spectrum (400 MHz, $CDCl_3$) of compound **5e**

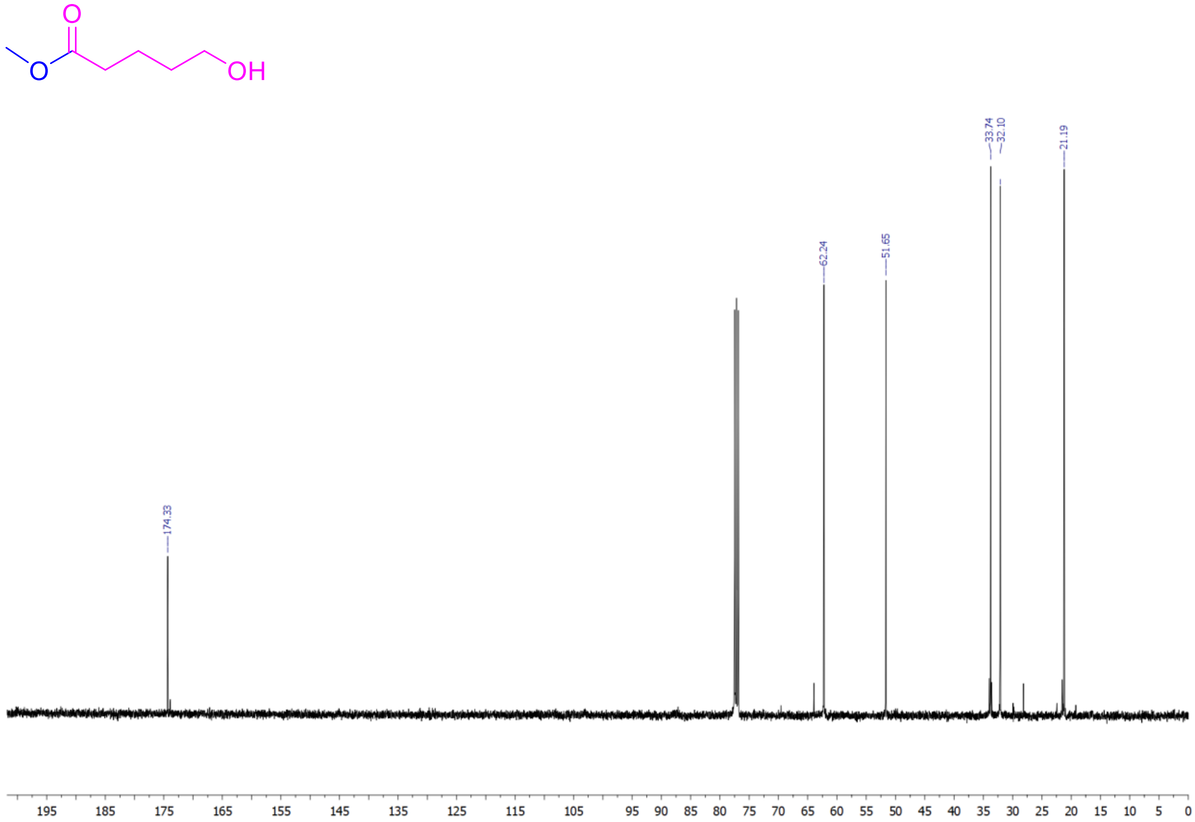

Figure S36. ^{13}C NMR spectrum (101 MHz, $CDCl_3$) of compound **5e**

3.19 Methyl pentanoate (5f)

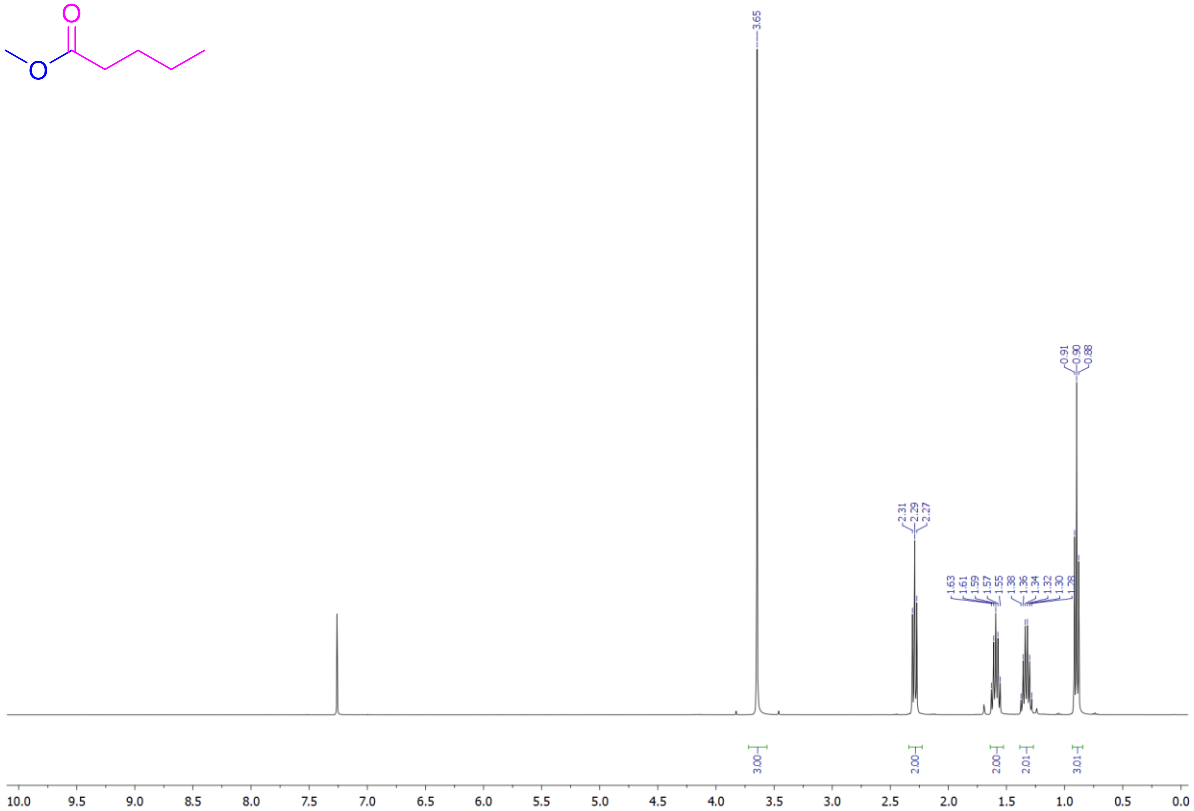

Figure S37. ^{1}H NMR spectrum (400 MHz, $CDCl_3$) of compound **5f**

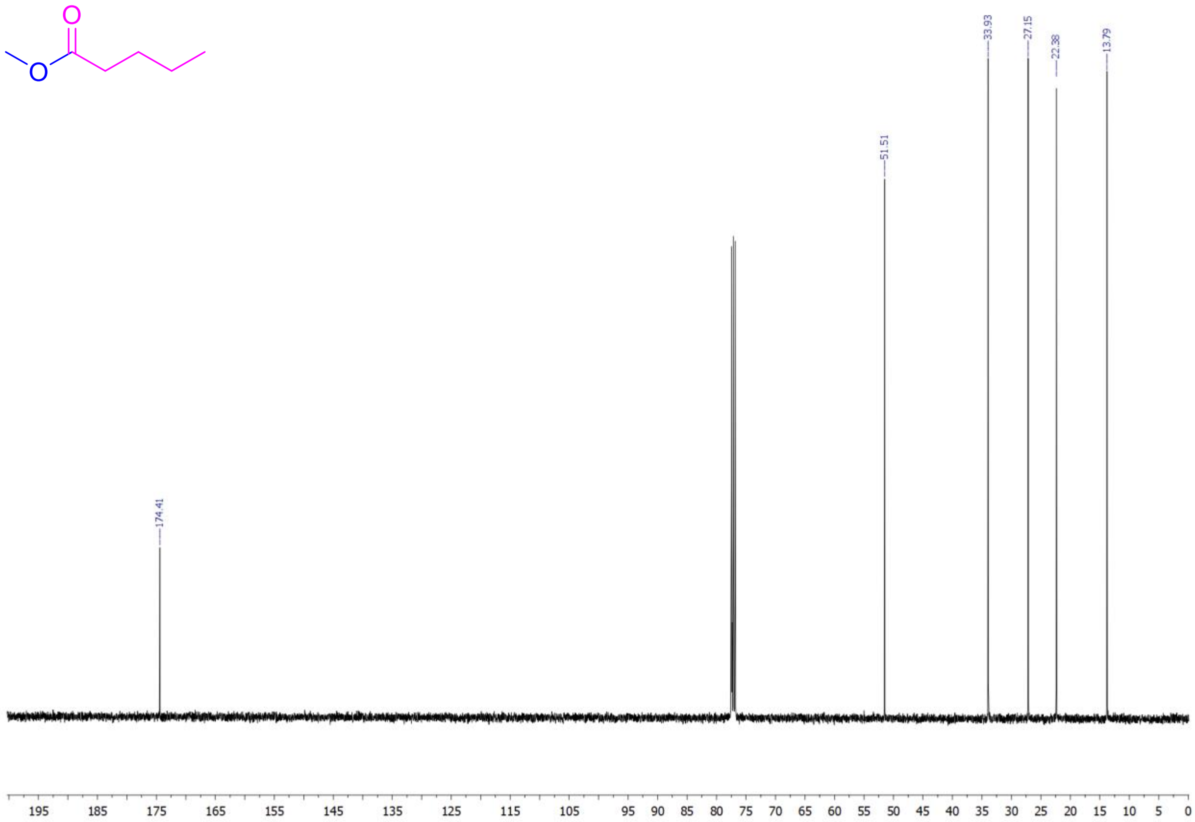

Figure S38. ^{13}C NMR spectrum (101 MHz, $CDCl_3$) of compound **5f**

3.20 Methyl nonanoate (5g)

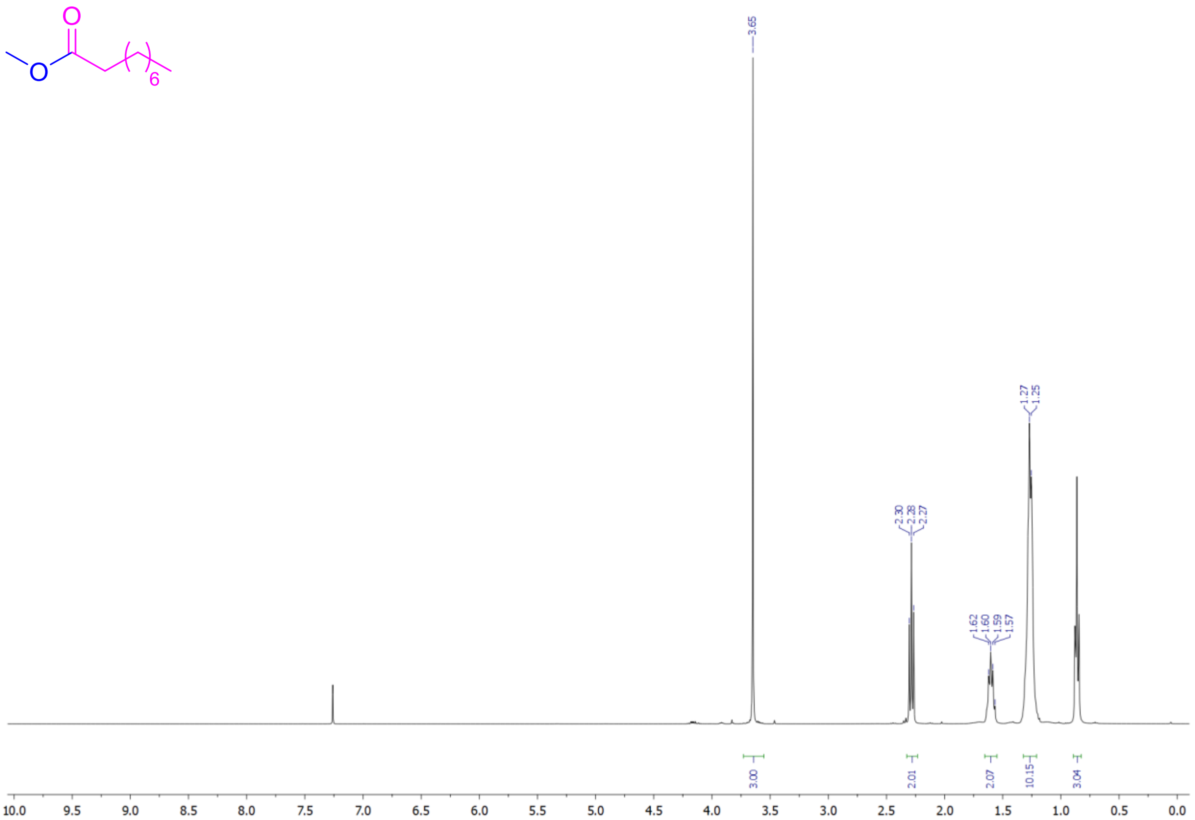

Figure S39. ^{1}H NMR spectrum (400 MHz, $CDCl_3$) of compound **5g**

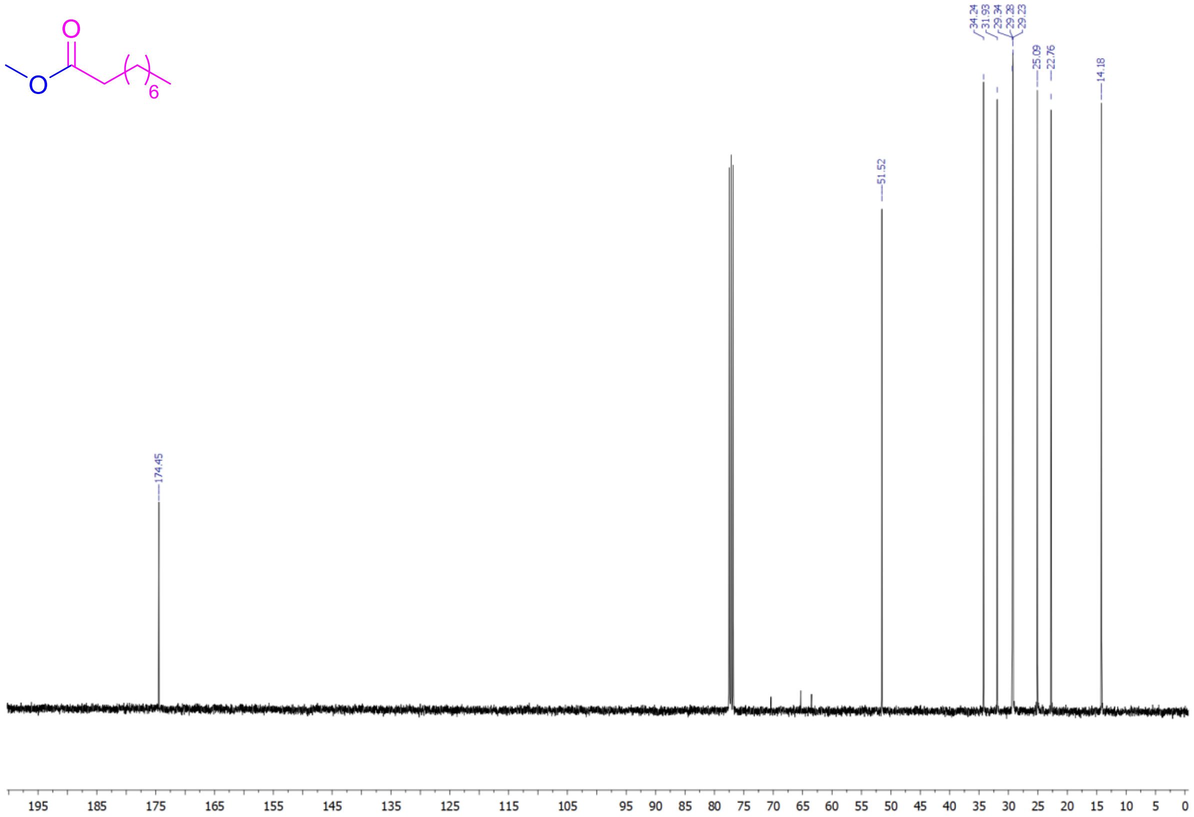

Figure S40. ^{13}C NMR spectrum (101 MHz, $CDCl_3$) of compound **5g**

3.21 Methyl decanoate (5h)

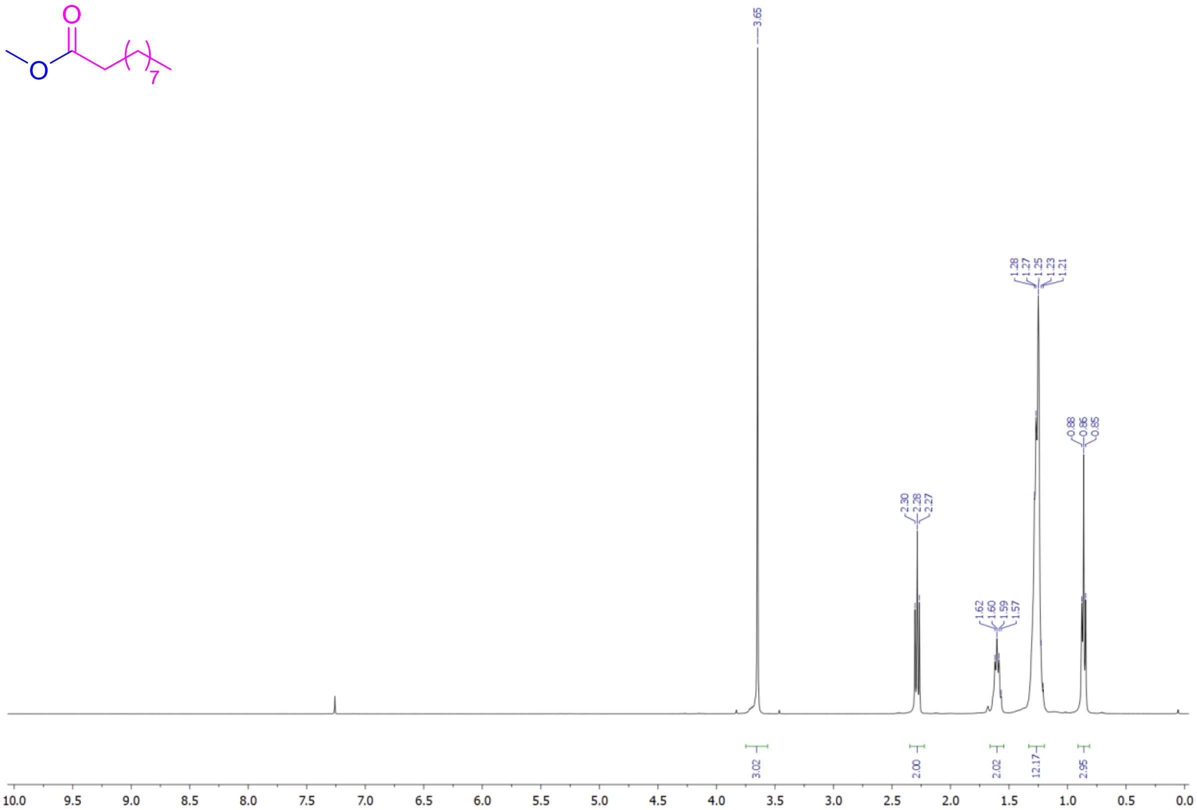

Figure S41. ^{1}H NMR spectrum (400 MHz, $CDCl_3$) of compound **5h**

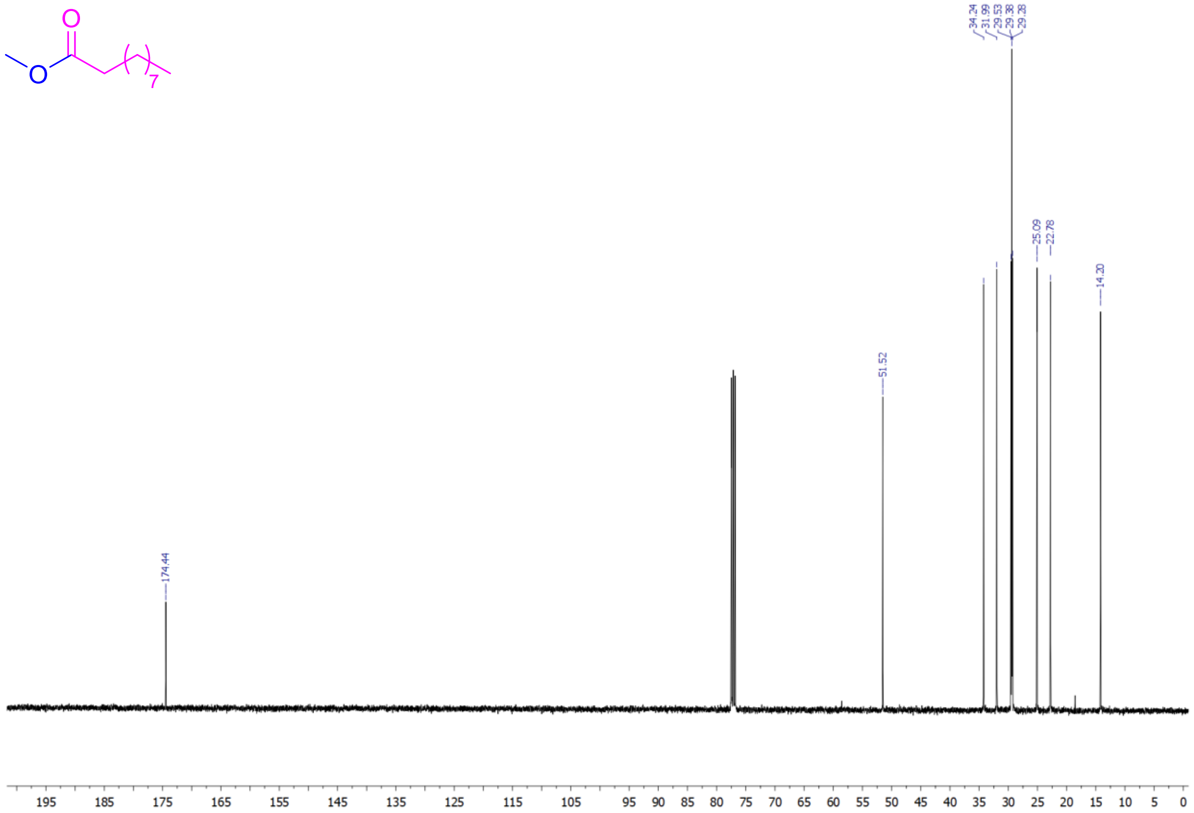

Figure S42. ^{13}C NMR spectrum (101 MHz, $CDCl_3$) of compound **5h**

3.22 Methyl dodecanoate (5i)

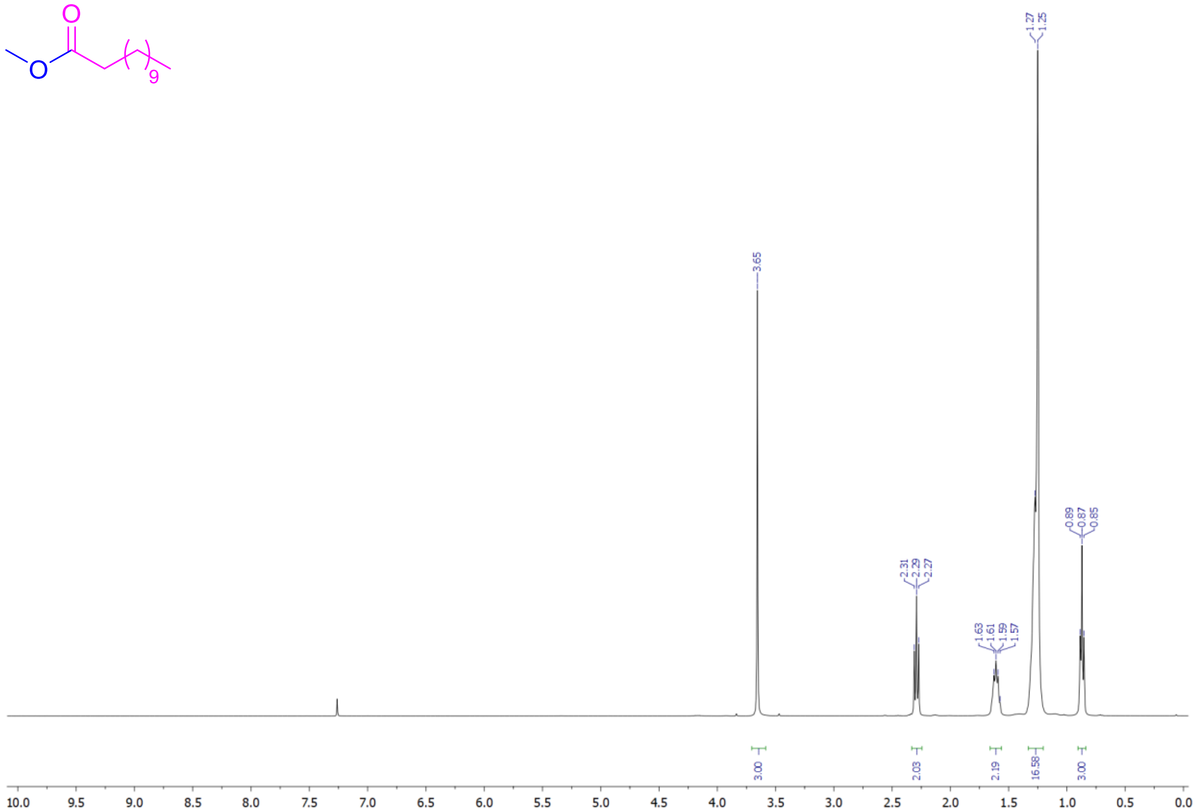

Figure S43. ^{1}H NMR spectrum (400 MHz, $CDCl_3$) of compound **5i**

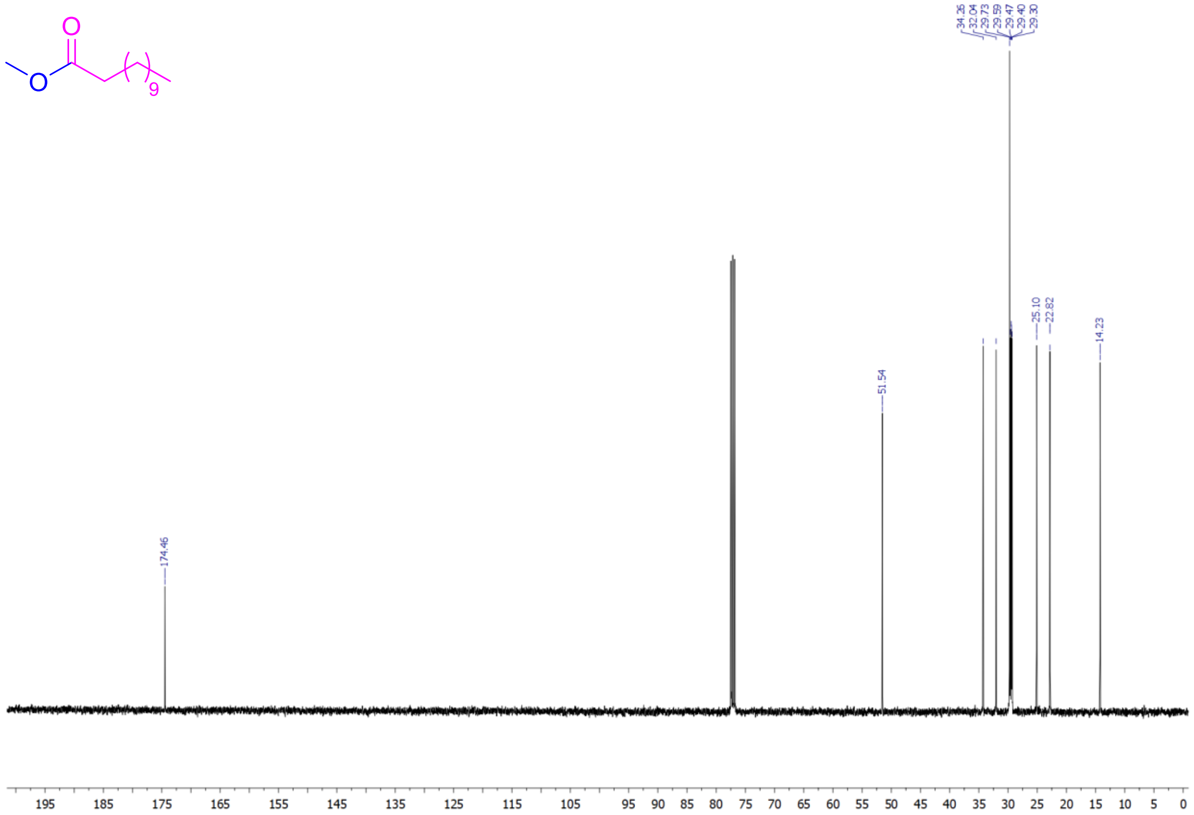

Figure S44. ^{13}C NMR spectrum (101 MHz, $CDCl_3$) of compound **5i**

4. Characterization of the catalyst CS_{600}

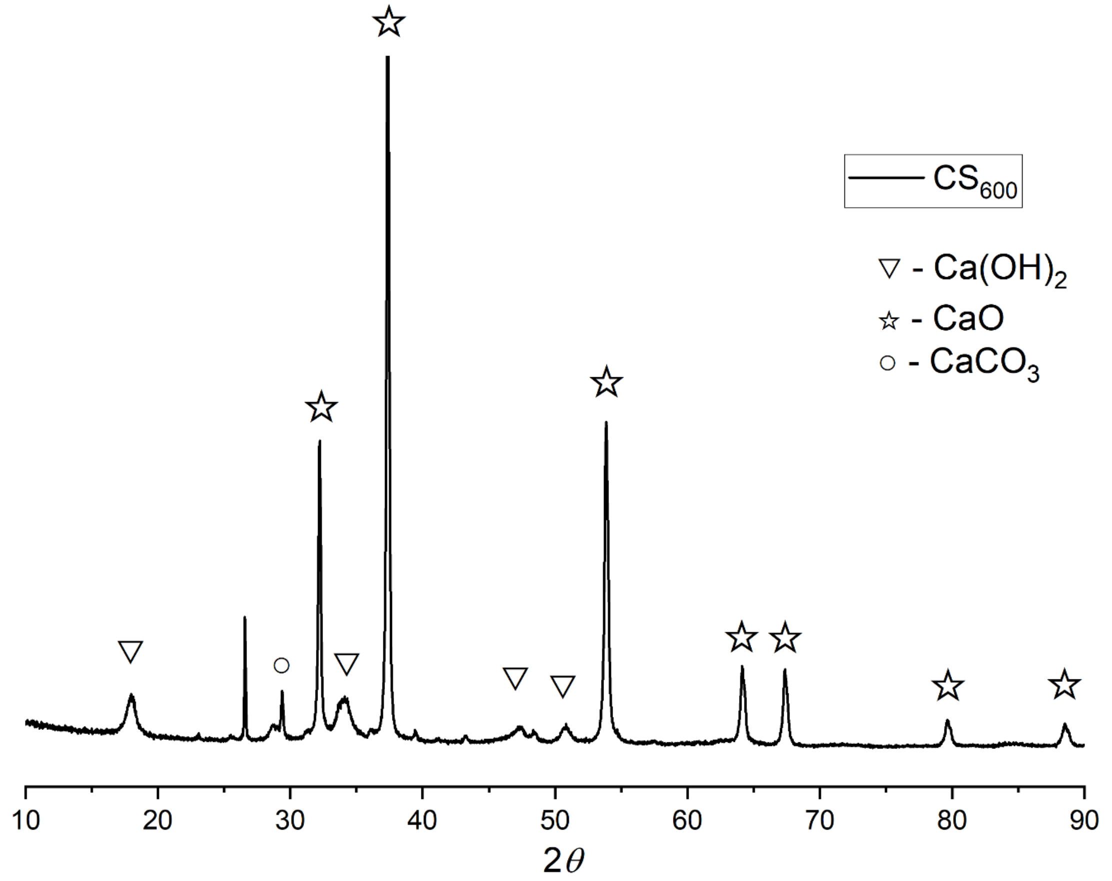

Figure S45. XRD pattern of CS_{600}.

Table S2. Qualitative analysis results of CS_{600}.

Phase name	Formula	Figure of merit	Phase reg. detail	DB card number
Lime, syn	CaO	0.426	ICDD (PDF-2 Release 2020 RDB)	01-077-9574
Portlandite, syn	$Ca(OH)_2$	0.657	ICDD (PDF-2 Release 2020 RDB)	01-076-0571
calcite	$CaCO_3$	0.981	ICDD (PDF-2 Release 2020 RDB)	01-083-4609
Graphite-2H, syn	C	0.848	ICDD (PDF-2 Release 2020 RDB)	00-056-0160
**Phase name**	**Formula**	**Space group**	**Phase reg. detail**	**DB card number**
Lime, syn	CaO	225 : Fm-3m	ICDD (PDF-2 Release 2020 RDB)	01-077-9574
Portlandite, syn	$Ca(OH)_2$	164 : P-3m1	ICDD (PDF-2 Release 2020 RDB)	01-076-0571
calcite	$CaCO_3$	167 : R-3c, hexagonal	ICDD (PDF-2 Release 2020 RDB)	01-083-4609
Graphite-2H, syn	C	194 : P63/mmc	ICDD (PDF-2 Release 2020 RDB)	00-056-0160

Table S3. Correlation of XRD peaks for CS_{600}.

No.	2-theta(deg)	d(ang.)	Chemical formula	Rel. int. I(a.u.)
1	18.012(16)	4.921(4)	$Ca(OH)_2$	4.60
2	23.098(8)	3.8475(14)	$CaCO_3$	0.65
3	26.578(2)	3.3512(2)	C	19.87
4	28.67(2)	3.111(3)	$Ca(OH)_2$	1.66
5	29.374(8)	3.0382(8)	$CaCO_3$	6.15
6	31.26(2)	2.859(2)	$CaCO_3$	0.68
7	32.190(3)	2.7786(2)	CaO	40.17
8	34.023(16)	2.6329(12)	$Ca(OH)_2$	5.01
9	36.071(13)	2.4880(9)	$CaCO_3$	0.82
10	37.344(3)	2.40607(19)	CaO	100.00
11	39.490(8)	2.2801(4)	$CaCO_3$	1.11
12	43.178(17)	2.0935(8)	$CaCO_3$, C	0.77
13	47.42(4)	1.9156(14)	$Ca(OH)_2$, $CaCO_3$	1.67
14	48.496(19)	1.8756(7)	$CaCO_3$	1.07
15	50.74(3)	1.7978(10)	$Ca(OH)_2$, C	1.87
16	53.844(3)	1.70128(10)	CaO	46.23
17	54.696(6)	1.67676(18)	C	1.17
18	57.50(2)	1.6015(6)	$CaCO_3$	0.34
19	62.58(7)	1.4831(15)	$Ca(OH)_2$, $CaCO_3$	0.38
20	64.125(6)	1.45108(12)	CaO, $Ca(OH)_2$	11.59
21	67.354(8)	1.38915(15)	CaO	10.46
22	71.7(3)	1.315(5)	$Ca(OH)_2$, $CaCO_3$, C	0.17
23	79.616(11)	1.20318(14)	CaO	4.00
24	84.69(11)	1.1435(12)	$Ca(OH)_2$, $CaCO_3$, C	0.30
25	88.511(19)	1.10381(19)	CaO	2.95

Table S4. Quantitative phase analysis of CS_{600} (wt %)

	CaO	$Ca(OH)_2$	$CaCO_3$	C	Rp (%)[a]
CS_{600}	68.9	24.9	5.2	1.0	7.5

[a] R_p is the coefficient of convergence of the calculated and experimental X-ray profiles.

Due to its high hygroscopicity, the sample quickly captured water molecules from the air and converted back to $Ca(OH)_2$ during the sampling and analysis stages, which indicates the presence of the $Ca(OH)_2$ phase in an amount of 24.9%

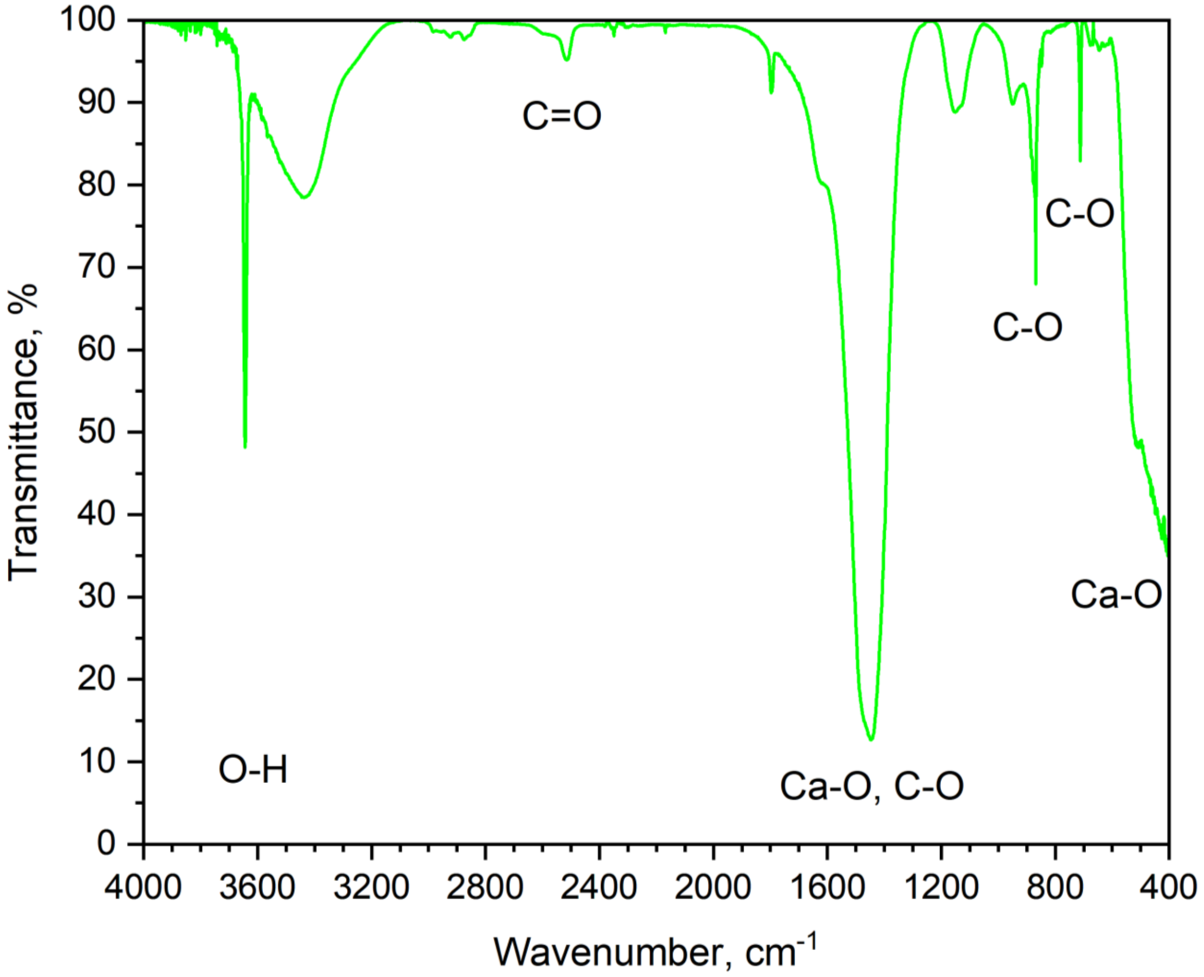

Figure S46. FTIR spectra of CS_{600}

5. References

1. Armarego, W. L. F.; Chai, C. L. L. *Purification of Laboratory Chemicals (Sixth Edition)*; Butterworth-Heinemann: Oxford, 2009.
2. Mitsudome, T.; Noujima, A.; Mizugaki, T.; Jitsukawa, K.; Kaneda, K. *Green Chem.* **2009**, *11*, 793-797. doi:10.1039/B900576E
3. Boulos Z.; Duceppe J.-S.; Penney C. METHOD FOR THE PREPARATION OF TRIGLYCERIDES OF MEDIUM-CHAIN LENGTH FATTY ACIDS. US Pat. Appl. US 9447016B2, Pat. US Patent 9447016B2, September 20, 2016.
4. Hu, Y.; Sang, R.; Vroemans, R.; Mollaert, G.; Razzaq, R.; Neumann, H.; Junge, H.; Franke, R.; Jackstell, R.; Maes, B. U. W.; Beller, M. *Angew. Chem. Int. Ed.* **2023**, *62*, e202214706. doi:10.1002/anie.202214706
5. Liu, C.; Wang, J.; Meng, L.; Deng, Y.; Li, Y.; Lei, A. *Angew. Chem. Int. Ed.* **2011**, *50*, 5144-5148. doi:10.1002/anie.201008073
6. Tran, U. P. N.; Oss, G.; Breugst, M.; Detmar, E.; Pace, D. P.; Liyanto, K.; Nguyen, T. V. *ACS Catal.* **2019**, *9*, 912-919. doi:10.1021/acscatal.8b03769
7. Cai, R.; Lu, M.; Aguilera, E. Y.; Xi, Y.; Akhmedov, N. G.; Petersen, J. L.; Chen, H.; Shi, X. *Angew. Chem. Int. Ed.* **2015**, *54*, 8772-8776. doi:10.1002/anie.201503546
8. Iwamoto, T.; Okuzono, C.; Adak, L.; Jin, M.; Nakamura, M. *Chem. Commun.* **2019**, *55*, 1128-1131. doi:10.1039/C8CC09523J
9. Reid, B. T.; Mailyan, A. K.; Zakarian, A. *J. Org. Chem.* **2018**, *83*, 9492-9496. doi:10.1021/acs.joc.8b01214
10. Jiang, X.; Zhang, J.; Zhao, D.; Li, Y. *Chem. Commun.* **2019**, *55*, 2797-2800. doi:10.1039/C8CC10315A
11. Zimmermann, F.; Meux, E.; Mieloszynski, J.-L.; Lecuire, J.-M.; Oget, N. *Tetrahedron Lett.* **2005**, *46*, 3201-3203. doi:10.1016/j.tetlet.2005.03.052
12. Dawar, P.; Bagavan, R. M.; and Ramakrishna, R. A. *Synth. Commun.* **2014**, *44*, 836-846. doi:10.1080/00397911.2013.837485
13. Xia, Q.; Liu, X.; Zhang, Y.; Chen, C.; Chen, W. *Org. Lett.* **2013**, *15*, 3326-3329. doi:10.1021/ol401362k
